# Bistability in Palladium Complexes with Two Different Redox‐Active Ligands of Orthogonal Charge Regimes

**DOI:** 10.1002/chem.202503160

**Published:** 2025-11-04

**Authors:** Franka Kreis, Andrei Poddelskii, Leon Hammermüller, Pascal Bootz, Franz Bodenmüller, Elisabeth Kaifer, Andreas Köhn, Hans‐Jörg Himmel

**Affiliations:** ^1^ Anorganisch‐Chemisches Institut Ruprecht‐Karls Universität Heidelberg Im Neuenheimer Feld 270 69120 Heidelberg Germany; ^2^ Institute for Theoretical Chemistry University of Stuttgart Pfaffenwaldring 55 70569 Stuttgart Germany

**Keywords:** bistability, guanidine, N ligands, palladium, redox isomerism

## Abstract

Electronically bistable molecular coordination compounds are obtained by a combination of two different redox‐active ligands with orthogonal charge regimes in square‐planar palladium complexes. Thereby, the first complexes are obtained in which two redox isomers are interconverted by interligand electron transfer between a catecholato/semiquinonato (dioxolene) ligand and a redox‐active guanidine (GFA) ligand. Experiments in alliance with quantum‐chemical calculations indicate that the barrier between the two states is relatively small. Due to the significantly different dipole moments of the two redox isomers, the intramolecular electron transfer can be triggered by variations in the solvent polarity. The energy difference between the two redox isomers and thus the ratio of their formation can be tuned by modifications at the dioxolene and GFA ligands.

## Graphical abstract

By assembling a redox‐active guanidine ligand together with a dioxolene ligand in cationic Pd complexes, bistable systems are formed in which the two redox isomers differ significantly in their dipole moment. The barrier for electron transfer is small, and the equilibrium can be shifted by change of the solvent polarity.

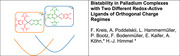

## 1 Introduction

**Figure 1:**
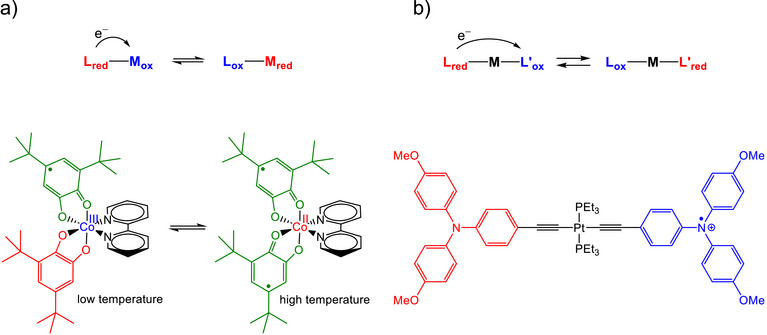
Bistability arising from ligand‐metal or interligand electron transfer. a) Archetypical example of ligand‐to‐metal charge transfer (also known as valence tautomerism).^[^ ^2^ ^]^ b) Symmetrically coordinated complex with observable ligand‐to‐ligand charge transfer.^[^ ^7^, ^8^ ^]^

Bistability of molecules, the coexistence of two stable and interconvertible states, is the basis for a variety of applications, where the response or function of molecules can be switched by external stimuli. These applications may range from photochromic molecules to molecular sensing or switchable molecular redox catalysis.^[^ ^1^ ^]^ Especially interesting cases for the design of switchable devices are molecules that contain two or more redox‐active units that exchange electrons (Figure 1). Here, intramolecular electron transfer leads to different states exhibiting distinct properties, for example characteristic electronic excitations in the visible or NIR region. The presence of both a redox‐active ligand and a redox‐active metal can give rise to valence tautomerism (Figure 1a).^[^ ^2^, ^3^, ^4^, ^5^, ^6^ ^]^ However, bistability could also arise from electron transfer between two different redox‐active ligands (Figure 1b). Most frequently, symmetric systems have been reported, which feature two distinct states with, however, indistinguishable properties. For instance, Marder and coworkers ^[^ ^7^ ^]^ reported a platinum compound with two ethinyl‐triphenylamine ligands in trans position (Figure 1b) and studied the intervalence charge‐transfer (IVCT) band that becomes visible in the NIR region. The work also pointed out similarities to an analogous organic mixed‐valence compound of Lambert and coworkers, ^[^ ^9^ ^]^ with the central platinum replaced by a phenyl bridge. In contrast to organic mixed‐valence compounds, ^[^ ^10^ ^]^ the compounds considered in this work feature a metal ion as a linker. Recently, an electrically conducting metal‐organic framework with Cd^II^ ions and functionalized radical monocationic TTF^·+^ linkers (TTF = tetrathiafulvalene) was reported.^[^ ^8^ ^]^ The inclusion of a metal ion allows a modular design with discrete redox units and offers additional opportunities, for example the participation of the metal in the electron transfer process.

**Figure 2:**
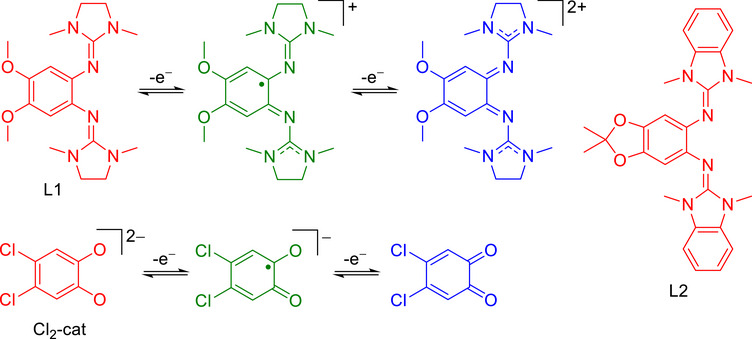
Redox states of diguanidines and dioxolenes highlighting their complementary charge regimes (0 to + 2 for diguanidines and ‐2 to 0 for dioxolenes). Reduced redox states in red, intermediate radical redox states in green, and oxidized redox states in blue.

Up to date, an unsymmetrically substituted bistable complex with distinguishable states that are interconverted by interligand electron transfer, has not been reported. Such a system requires the presence of two different redox‐active ligands, which are designed such that large charge accumulation is avoided. To this end, we have in a series of previous studies ^[^ ^11^, ^12^, ^13^ ^]^ developed guanidino‐functionalized aromatic compounds (GFAs) as a class of redox‐active ligands with a charge regime opposite to that of dioxolene (catecholato/semiquinonato/quinonato) ligands (Figure 2). Recently, we reported the synthesis and properties of neutral cobalt complexes with two dioxolene and one GFA ligands.^[^ ^14^, ^15^ ^]^ Intriguingly, the electron distribution in these complexes can be varied by modification of the dioxolene and GFA ligands.

In the present work, we explore the electron distributions in monocationic tetracoordinate palladium complexes with one GFA and one dioxolene ligand. The dioxolene ligand was varied from the electron‐rich 4‐tert‐butyl‐catecholato (*t*Bu‐cat) form to the less electron rich 4,5‐dichlorocatecholato (Cl_2_‐cat) and tetrachlorocatecholato (Cl_4_‐cat) ligands. As GFA ligands, we used L1 and a related diguanidine ligand L2 (Figure 2).^[^ ^16^ ^]^ These two ligands were chosen due to their relatively low redox potentials; in the cyclic voltammetry (CV) curves, recorded for dichloromethane (DCM) solutions, the *E*_1/2_ values for the first redox couple is ‐0.43 V for L1 and ‐0.20 V for L2 (with respect to the reference redox couple ferrocenium/ferrocene).

## 2 Results and Discussion

**Figure 3:**
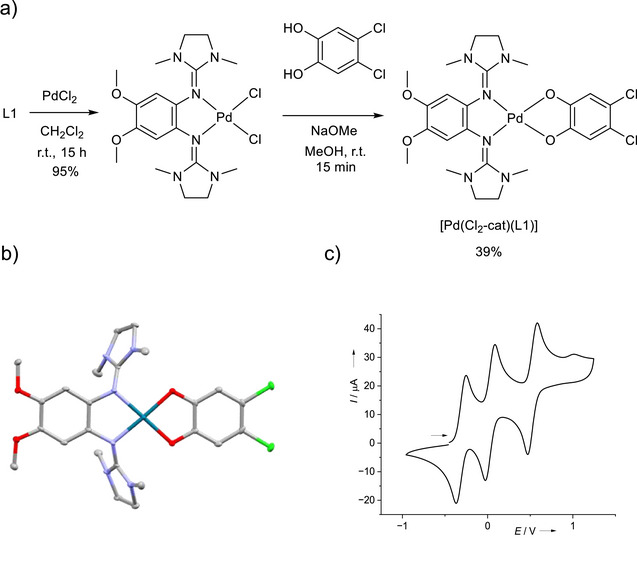
a) Synthesis of [Pd(Cl_2_‐cat)(L1)] in two steps. b) Illustration of its solid‐state structure. Displacement ellipsoids drawn at the 50% probability level. Hydrogen atoms are omitted. Colour code: C grey, O red, N light blue, Cl green, Pd blue. c) CV curve (*^n^* Bu_4_PF_6_ as supporting electrolyte, 100 mV s^−1^ scan speed) recorded for the complex in dichloromethane. Potentials given versus the reference redox couple ferrocenium/ferrocene (Fc^+^/Fc).

In order to design the first complex with two different redox‐active ligands that shows bistability due to ligand‐ligand electron transfer, palladium was chosen as metal, since the square‐planar geometry of its complexes warrants sufficient orbital overlap for electron‐transfer processes between the ligands, while the palladium center itself will be shown to preserve its oxidation state in the course of intramolecular electron transfer. GFA L1 was reacted with PdCl_2_ to give the complex [PdCl_2_(L1)] in 95% yield (Figure 3a). Subsequently, this complex was reacted with one of the catechols in the presence of a strong base (NaOMe) in methanol solution. In this way, the new complex [Pd(Cl_2_‐cat)(L1)], exhibiting a GFA as well as a dioxolene ligand bridged by palladium, was isolated as green solid in a yield of 39% from the dichloropalladium precursor. The complex was crystallized and structurally characterized by SC‐XRD (Figure 3b and Table 1). As expected, the complex has a square‐planar geometry. The metrical oxidation state (MOS) of the dioxolane ligand, estimated from selected structural parameters by the algorithm of Brown,^[^ ^17^ ^]^ clearly argues for the presence of a catecholato ligand (MOS = ‐1.8). Moreover, the structural parameters of the GFA ligand are in line with a neutral, reduced redox state. In the CV curve (Figure 3c), the new complex [Pd(Cl_2_‐cat)(L1)] shows three quasi‐reversible one‐electron redox steps, at *E*_1/2_ = ‐0.31 V (*E*_ox_ = ‐0.25 V), 0.03 V (0.09 V) and 0.53 V (0.58 V) versus the reference redox couple ferrocenium/ferrocene.

**Table 1:** Selected bond lengths (in Å) for the neutral and monocationic Pd complex from SC‐XRD measurements, as well as the metrical oxidation states (MOS)^[^ ^17^ ^]^ of the dioxolene ligands. For comparison, we also show here the calculated structure parameters at the LC‐ωPBE‐D3/def2‐SVP, COSMO(ε=∝) level of theory.

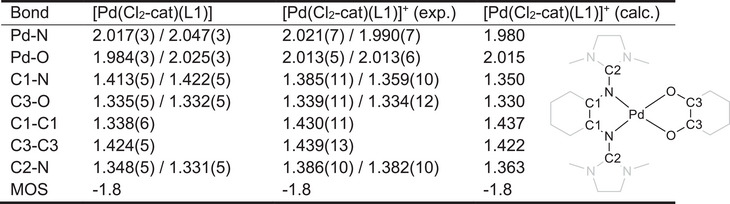

**Figure 4:**
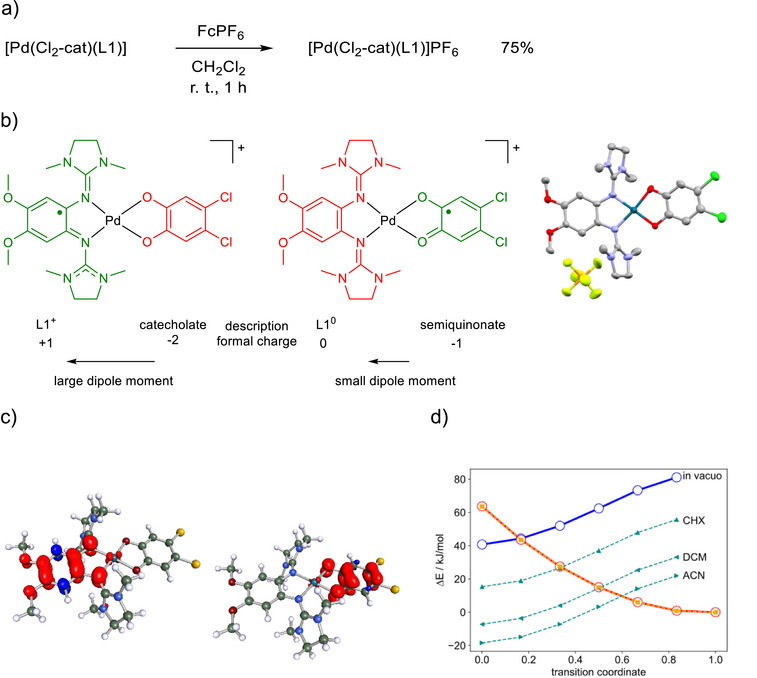
a) Reaction scheme of the chemical oxidation. b) Redox isomers of a cationic palladium complex with one guanidine and one dioxolene ligand, that are interconverted by interligand electron transfer. The crystal structure is displayed on the right side. c) Computed spin‐density of the guanidino radical (left) and the dioxolene radical (right) redox isomers (LC‐wPBE‐D3/def2‐SVP). d) Computed energies of the guanidino radical (blue) and the dioxolene radical (red) along the linear synchronous transit path (0: guanidino radical structure; 1: semiquinonato structure). The open points linked by a solid line are computed energies (PNO‐CCSD(T)‐F12 using DFT structures) for the isolated systems (blue: guanidino radical state, red: dioxolene radical state). The effect of solvation is simulated by shifting the curves by the computed solvent stabilisation energies (dCOSMO‐RS) for the respective equilibrium states (broken lines; CHX = cyclohexane, DCM = dichloromethane, ACN = acetonitrile). The zero point of the energy scale is always set to the semiquinonato state.

Subsequently, we chemically oxidized the neutral complex to the radical monocation using one equivalent of ferrocenium hexafluorophosphate. The salt [Pd(Cl_2_‐cat)(L1)]PF_6_ was obtained in 75% isolated yield (Figure 4a). The purity was evidenced by elemental analysis. From the analytical data (especially SC‐XRD and EPR), the monocation clearly exhibits a next to square‐planar coordinated Pd^II^ (d^8^, no unpaired electron). This implies that there is one unpaired electron on one of the ligands. As shown in Figure 4b, the radical monocation can be present in two different redox‐isomeric forms. An assignment of these forms to the experimentally obtained compounds requires a detailed analysis. The salt [Pd(Cl_2_‐cat)(L1)]PF_6_ crystallized and was structurally characterized by SD‐XRD (Figure 4b, some structural parameters can be found in Table 1). The complex remains nearly square planar and the MOS value (‐1.8) indicates the presence of a catecholato unit. We can conclude that in the solid state the GFA ligand is oxidized to the radical monocationic redox state. However, we will see in the following that the electron distribution can be different in solution.

In order to shed more light on the electronic structure, ab initio computations were carried out, using density functional theory (DFT) and a range‐separated functional (LC‐wPBE‐D3/def2‐SVP),^[^ ^18^, ^19^ ^]^ accounting for solvent effects by a continuum solvation model (COSMO^[^ ^20^ ^]^ and dCOSMO‐RS^[^ ^21^ ^]^). High‐level energy corrections were added at the coupled‐cluster level (PNO‐LCCSD(T)‐F12/cc‐pVDZ‐F12),^[^ ^22^, ^23^, ^24^ ^]^ see Supporting Information Section S1.3 for all details. As seen from Table 1, the calculated and experimental structural parameters are in good agreement. The computations allow to identify two distinct ground state minima, one corresponding to a complex with an oxidized GFA and a catecholate ligand and the other to a complex with a reduced, neutral GFA and a semiquinonato (SQ) ligand (Figure 4b). The corresponding spin density distribution is plotted in Figure 4c. Note that both states feature a nonvanishing spin‐density at the Pd atom. The presence of two ground state minima in the computations clearly indicates a bistability of the system. We also find a strong change of the dipole moment of approximately 18 Debye units (note that the absolute value of the dipole moments is not well‐defined for charged molecules). This strong change in charge distribution also leads to a significant influence of the solvent polarity on the relative stability of the redox isomers, as indicated in Figure 4d. The figure shows the run of the energy of both states as a function of a linear synchronous transition coordinate between the two obtained minima, shifted by the relative solvation stabilization energy. With increasing solvent polarity, the energy of the guanidine radical state (blue curve) decreases, due to its large dipole moment. The computations indicate a small electronic coupling of the two states, but a not too large barrier of less than 20 kJ mol^−1^ in the limit of equal free energy for both redox isomers.

The computed relative energies of the two redox isomers of [Pd(Cl_2_‐cat)(L1)]^+^ are listed in Table 2 along with variants of the complex using modified ligands (see discussion below and Supporting Information). These numbers highlight the strong solvent effect. Without consideration of solvent effects, the structure with a neutral GFA and a SQ ligand is most stable in all cases. This picture also largely holds for nonpolar solvents, while the isomer with a GFA radical and a catecholato ligand is favored with increasing relative solvent permittivity.

**Table 2:** Computed and experimentally estimated energy differences (∆*G* (298.15 K) in kJ mol^−1^) between the guanidino radical and the dioxolene radical form of the Pd complexes. Negative sign indicates more stable guanidino radical form.

	[Pd(Cl_4_‐cat)(L1)]^+^	[Pd(Cl_2_‐cat)(L1)]^+^	[Pd(*t*Bu‐cat)(L1)]^+^	[Pd(Cl_4_‐cat)(L2)]^+^	[Pd(Cl_2_‐cat)(L2)]^+^	[Pd(*t*Bu‐cat)(L2)]^+^
∆*G*(calc)^[^ ^a^ ^]^	13.5	35.0	58.4	20.2	39.0	63.6
∆*G*(calc, CHX)^[^ ^b^ ^]^	−6.0	10.2	32.9	−0.3	18.1	40.4
∆*G*(calc, DCM)^[^ ^b^ ^]^	−26.3	−12.4	12.5	−24.3	−7.9	16.4
∆*G*(calc, ACN)^[^ ^b^ ^]^	−43.1	−23.7	2.9	−38.7	−17.9	10.2
∆*G* (exp, DCM)^[^ ^c^ ^]^		−5.2	+1.5			+0.7

^[a]^ Computed using PNO‐LCCSD(T)‐F12a/cc‐pVDZ‐F12 electronic energies based on DFT/LC‐ωPBE‐D3/def2‐SVP geometries, including zero‐point vibrational energy and chemical potential (298.15 K) contributions at DFT/LC‐ωPBE‐D3/def2‐SVP level;

^[b]^ Energies computed as before with additional solvation energy contributions at DFT/LC‐ωPBE‐D3(BJ)/def2‐SVP, dCOSMO‐RS level using parameter sets for cyclohexane (CHX), dichloromethane (DCM), and acetonitrile (ACN)

^[c]^ Values estimated from fits of EPR spectra.

**Figure 5:**
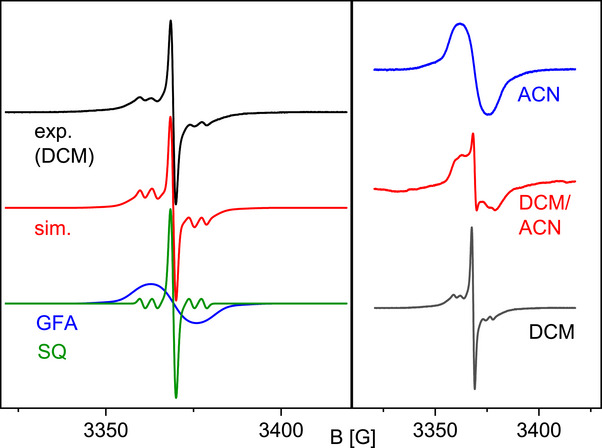
X‐band EPR spectra of [Pd(Cl_2_‐cat)(L1)]PF_6_. Left: Experimental spectrum and simulation with contributions from both redox isomers. Right: EPR spectra in different solvents. In ACN, the complex is exclusively present as redox isomer with the unpaired electron on the GFA (broad signal), and in DCM the redox isomer with SQ ligand is present. In the solvent mixture, DCM and CAN are mixed in equal parts.

The X‐band EPR spectrum of the oxidized complex in CH_2_Cl_2_ solutions (Figure 5) contains a sharp signal and hyperfine coupling to ^105^Pd, pointing to some participation of the Pd orbitals in the magnetic orbitals. A simulation (using Easyspin 6.0.2,^[^ ^25^ ^]^ see Supporting Information Section S5.2 for details) indicates the presence of both redox isomers in solution. The g values obtained for both components are in line with organic radicals (2.0039 (GFA) and 2.0038 (SQ)). The isomer with a SQ ligand, [Pd(Cl_2_‐sq)(L1)]^+^, is responsible for the sharp signal in the center, while the broadened signal belongs to the isomer with the unpaired electron on the radical monocationic diguanidine ligand, [Pd(Cl_2_‐cat)(L1**·**)]^+^, due to the spin density on the nitrogen atoms. The semiquinonate redox isomer shows well‐resolved hyperfine coupling on ^105^Pd (nat. abund. 22.3%, *I *= 5/2, A = 3.5 G). The GFA radical shows hyperfine coupling of 4.7, 3.8 and 1.3 G for ^105^Pd, two N atoms and additional four N atoms. These values are also in line with ab initio values (see Supporting Information Section S7, Table S5 for details) which give g values of 2.0058 (GFA, anisotropy 0.05) and 2.0019 (SQ, anisotropy 0.02). In particular, they support a nonvanishing spin density at the Pd atom, giving hyperfine couplings of 4.9 G (GFA) and 3.0 G (SQ) and the presence of N hyperfine interactions (2.9 G for two N and 1.1 G for four N).

Based on the fit of the EPR spectrum, the quantity of semiquinone‐based radical amounts to approximately 11% for [Pd(Cl_2_‐cat)(L1)]^+^. X‐band EPR measurements at variable temperature (200 K–300 K) in CH_2_Cl_2_ show no significant change in the EPR signal for [Pd(Cl_2_‐cat)(L1)]PF_6_. From $\Delta G=\mathrm{RT}\ln(\frac{0.89}{0.11})$ we can estimate an energy difference of ‐5.2 kJ mol^−1^ at 300 K (a minus sign indicates a more stable GFA radical). Our ab initio computed best estimate is ‐12.4 kJ mol^−1^ at 298.15 K.

The influence of the polarity of the environment was also investigated by changing the solvent from CH_2_Cl_2_ to CH_3_CN to stabilise the redox isomer with the larger dipole moment in a polar environment. Figure 5 shows the X‐band EPR spectra of [Pd(Cl_2_‐cat)(L1)]PF_6_ in CH_2_Cl_2_ and CH_3_CN. A broad signal is observed for [Pd(Cl_2_‐cat)(L1)]PF_6_ in ACN, which indicates a GFA centered radical. In the first derivative of the signal, no asymmetry (indicating an overlay of two signals) can be observed. This is additionally confirmed by comparison with the X‐band EPR signal of the respective onefold oxidized palladium chloride complex ([PdCl_2_(L1)]PF_6_, see Supporting Information, Section S5.3, Figure S59). Here, the unpaired electron is clearly located on the GFA, being the only redox‐active ligand. In the EPR spectrum, a broad signal similar to that observed for [Pf(Cl_2_‐cat)(L1)]PF_6_ in ACN appeared due to unresolved hyperfine coupling to hydrogen and nitrogen (see Supporting Information, p. 35, Figure S42). Figure 5 also shows the EPR spectrum of [Pd(Cl_2_‐cat)(L1)]PF_6_ in a 1:1 mixture of CH_2_Cl_2_ and CH_3_CN. The simulation of this spectrum indicates that 3% of the complexes exhibit a semiquinone‐based radical. The higher sensitivity of the oxidized complex towards polarity of the environment, compared to its sensitivity with respect to a change of temperature, is in line with our approach to generate redox isomers with strongly different dipole moments. In solid‐state X‐band EPR only a broad signal indicating a guanidine‐based radical is observed, matching the observation from the solid‐state structure.

**Figure 6:**
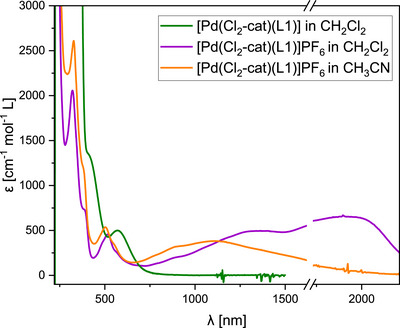
UV‐vis spectra of [Pd(Cl_2_‐cat)(L1)] and [Pd(Cl_2_‐cat)(L1)]PF_6_, measured in CH_2_Cl_2_ or CH_3_CN solutions.

UV‐vis spectra of the oxidized, monocationic complex (Figure 6) show broad, intense absorptions in the NIR region, that are assigned to intervalence (ligand‐ligand) charge‐transfer (IVCT) transitions. The respective onefold oxidized palladium chloride complex [PdCl_2_(L1)]PF_6_ has no absorption in this region. For [Pd(Cl_2_‐cat)(L1)], bands at 1940 nm (ε= 0.65·10^3 ^M^−1^ cm^−1^) and 1365 nm (0.50·10^3 ^M^−1^ cm^−1^) were measured in DCM. In ACN, the IVCT bands change noticeably: Only one markedly blue‐shifted band is observed at 1118 nm (0.38·10^3 ^M^−1^ cm^−1^). This is in accordance with the EPR experiment in ACN, which indicates that only the GFA centered radical is found in [Pd(Cl_2_‐cat)(L1)]PF_6_ with highly polar solvents. This clearly corroborates the assignment of the bands as IVCT transitions and fits to the energy range suggested by the computations (compare Figure 4d and Table 2). In fact, the positions and the solvent shifts of the bands fit well to a simple Marcus‐Hush like picture ^[^ ^26^, ^27^, ^28^ ^]^ (see Supporting Information, Section S6.3 for details). The 1365 nm band corresponds to the IVCT excitation of the GFA centered radical and the stabilization of this state by ACN is expected to induce a blue shift, as observed. This can be interpreted as a stabilization of the diguanidine‐centered radical by approximately 19 kJ mol^−1^ (the stabilization predicted by the dCOSMO‐RS model is 11 kJ mol^−1^, see Table 2). The 1940 nm band is due to the semiquinonate and vanishes for ACN solvent, as the state becomes destabilized and its thermal occupation drops significantly. The spectra of the redox isomers differ primarily in the NIR region; therefore, the difference in color is not very large.

**Figure 7:**
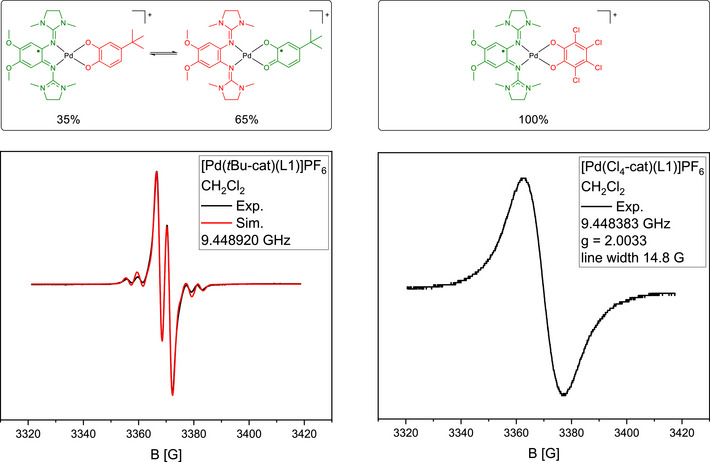
X‐band EPR spectra for [Pd(*t*Bu‐cat)(L1)]PF_6_ (left) and [Pd(Cl_4_‐cat)(L1)]PF_6_ (right) in CH_2_Cl_2_ solutions (room temperature). The result of a simulation (in red) is shown for [Pd(*t*Bu‐cat)(L1)]PF_6_.

To test the bistability further, we synthesized additional complexes with catecholates of different redox potential and two different GFA ligands (see Supporting Information for details). The structural characterization in the solid state and X‐band EPR spectroscopy in the solid state showed that the redox isomer with a catecholate and a radical monocationic GFA ligand dominates in the solid state. The MOS values of the oxidized complexes are all within the range (‐1.8)–(‐1.9), in line with the presence of a catecholate ligand. In solution, the ratio between the two redox isomers depends on the nature of the dioxolene ligand and the environment. Figure 7 compares the X‐band EPR spectra recorded for [Pd(Cl_4_‐cat)(L1)]PF_6_ and [Pd(*t*Bu‐cat)(L1)]PF_6_. The hyperfine splitting in the EPR spectrum of [Pd(*t*Bu‐cat)(L1)]PF_6_ clearly points to a significant amount of the SQ form. The proton in the asymmetric (*t*Bu‐cat) backbone leads to additional hyperfine coupling. A simulation gave 65% SQ isomer. With $\Delta G=\mathrm{RT}\ln(\frac{0.35}{0.65})$, the estimated energy difference is 1.5 kJ mol^−1^ at 300 K in favor of the SQ form. This preference of the semiquinonate radical is in line with ab initio computations, although the observed trend is weaker than expected (computed ∆*G* = 12.5 kJ mol^−1^ at 298.15 K). On the other hand, the spectrum of [Pd(Cl_4_‐cat)(L1)]PF_6_ indicates 100% of the catecholate isomer.

Variation of the GFA ligand, that is, replacing L1 by L2, leads to smaller differences, as expected from the relatively similar redox potentials of the neutral complexes (see Supporting Information).

For all investigated cationic complexes, intense IVCT bands are visible in the UV‐vis spectra. For (*t*Bu‐cat) a slight red‐shift in the IVCT region is observed upon increase of the solvent polarity, while a significant blue‐shift is observed for (Cl_4_‐cat). All these results demonstrate the possibility to vary the position of the equilibrium between the two redox isomers by modifying the redox‐active ligands and by environmental (solvent) effects.

## 3 Conclusions

By combination of two ligands with orthogonal charge regimes in a single square‐planar palladium complex, the first example for electronic bistability of mononuclear complexes with two distinctly different electron distributions in the two states is obtained, in which the two redox isomers are interconverted by interligand electron transfer. Due to the different charge accumulations, the two redox isomers differ significantly in their dipole moment, allowing to switch between the two states by the solvent polarity. The ratio of the two redox isomers in solution was evaluated from simulations of the X‐band EPR spectra. The presence of large Pd hyperfine splitting highlights the role of the metal atom as a linker that mediates electron transfer between the redox‐active ligands in a modular system. Moreover, each redox isomer exhibits a characteristic IVCT band in the electronic excitation spectra. Quantum‐chemical calculations confirm the presence of two stable redox isomers that are interconverted by a relatively low barrier, and the strong environmental (solvent) effect on the position of the equilibrium between both stable states. The results of this work demonstrate that switchable molecules could be built by the concept of combination of two redox‐active ligands with orthogonal charge regimes in one complex. In ongoing work, we tune the barrier between the two states by changing the metal that connects the two redox‐active ligands, to achieve optimal switching properties, thereby preparing these unprecedented systems for novel applications in switching devices.

## 4 Experimental Details

The synthesis details and analytical data for all compounds and information about the quantum‐chemical calculations are included in the Supporting Information. Deposition Numbers 2 482 872 for [PdCl_2_(L1)], 2 482 875 for [PdCl_2_(L1)]PF_6_, 2 482 877 for [Pd(Cl_2_‐cat)(L1)], 2 482 873 for [Pd(Cl_2_‐cat)(L1)]PF_6_, 2 482 874 for [Pd(Cl_4_‐cat)(L1)], 2 482 880 for [PdCl_2_(L2)], 2 482 876 for [Pd(*t*Bu‐cat)(L2)](PF_6_)_2_, 2 482 881 for [Pd(Cl_2_‐cat)(L2)], 2 482 878 for [Pd(Cl_2_‐cat)(L2)]PF_6_, 2 482 879 for [Pd(Cl_4_‐cat)(L2)], 2 482 933 for [Pd(Cl_4_‐cat)(L2)]PF_6_ contain the supplementary crystallographic data for this paper. These data are provided free of charge by the joint Cambridge Crystallographic Data Centre and Fachinformationszentrum Karlsruhe Access Structures service.

## Supporting Information

The authors have cited additional references within the Supporting Information.^[^ ^29^, ^30^, ^31^, ^32^, ^33^, ^34^, ^35^, ^36^, ^37^, ^38^, ^39^, ^40^, ^41^, ^42^, ^43^, ^44^, ^45^, ^46^, ^47^, ^48^, ^49^, ^50^, ^51^, ^52^, ^53^, ^54^, ^55^, ^56^, ^57^, ^58^, ^59^, ^60^, ^61^, ^62^ ^]^

## Conflicts of Interest

The authors declare no conflict of interest.

## Supporting information



Supporting Information

Supporting Information

## Data Availability

The data that support the findings of this study are available in the supplementary material of this article.
