# Supplementary material for: Bistability in Palladium Complexes with Two Different Redox‐Active Ligands of Orthogonal Charge Regimes
Source: Chemistry. 2025 Nov 4;31(69):e03160. doi: 10.1002/chem.202503160 (PMC12699171; doi:10.1002/chem.202503160)
Supplement: Supplementary file 1 — Supporting Information [file CHEM-31-e03160-s001.pdf]

Supporting Information

Bistability in Palladium Complexes with Two Different Redox-Active Ligands of Orthogonal Charge Regimes

Franka Kreis, Andrei Poddelskii, Leon Hammermüller, Pascal Bootz, Franz Bodenmüller, Elisabeth Kaifer, Andreas Köhn,* Hans-Jörg Himmel*

S1 General Information 2
S1.1 Experimental details 2
S1.2 X-ray crystallography 2
S1.3 Ab initio calculations 3
S2 Synthetic protocols 5
S2.1 Compounds with L1 5
S2.2 Compounds with L2 12
S3 NMR spectroscopy 20
S4 Cyclic voltammetry 30
S5 X-Band EPR spectroscopy 35
S5.1 Spectra in CH_2Cl_2 solution at room temperature 35
S5.2 Simulations with EasySpin 38
S5.3 Additional EPR spectra 43
S5.4 Solid state spectra 48
S6 UV-vis spectroscopy 51
S6.1 Spectra in nm 51
S6.2 Tabular summary 59
S6.3 Interpretation of IVCT bands by Marcus-Hush model 60
S7 Ab initio calculations 62
S8 X-ray crystallography 64
S8.1 Comparision of structural data 64
S8.2 Crystallographic data 66
S9 Literature 70

* F. Kreis, A. Poddelskii, E. Kaifer, H.-J. Himmel
Anorganisch-Chemisches Institut, Ruprecht-Karls Universität Heidelberg, Im Neuenheimer Feld 270, 69120 Heidelberg, Germany
A. Köhn
Institute for Theoretical Chemistry, University of Stuttgart, Pfaffenwaldring 55, 70569 Stuttgart, Germany
Corresponding authors: Email: koehn@theochem.uni-stuttgart.de, hans-jorg.himmel@aci.uni-heidelberg.de

S1 General Information

S1.1 Experimental details

All reactions were carried out under a dry argon atmosphere by using standard Schlenk technique. The solvents acetonitrile, dichloromethane, diethyl ether and tetrahydrofuran were dried with a MBraun Solvent Purification System and stored over molecular sieves (4 Å) after being degassed. Other solvents were purchased from Acros Organics and degassed and stored similarly. The following chemicals were purchased and used as delivered: Palladium chloride, sodium methanolate, 3,4,5,6-Tetrachlorobenzene-1,2-diol, 4,5-Dichlorobenzene-1.2-diol, 4-tert-Butylpyrocatechol. The ligands L1 and L2[1], as well as ferrocenium hexafluorophosphate[2] were prepared according to literature know procedures.

The analytical data was plotted using OriginPro 2023b (64-bit) SR1. UV-vis spectra were measured on a Varian Cary 5000 spectrophotometer. NMR spectra were recorded with BRUKER Avance II 400, BRUKER Avance III 600 and Bruker 600 Ultrashield systems at 298 K. Solvent resonances were taken as references for all 1H NMR spectra. Analyses of the spectra were performed using MestReNova. Elemental analysis were performed at the Microanalytical Laboratory of Heidelberg University using the vario EL and varioMICRO cube devices from Elementar Analysensysteme GmbH. Mass spectrometric measurements were made on a BRUKER ApexQe hybrid 9.4 T FT-ICR maschine (ESI) or a Finnigan LCQ quadrupole ion trap (ESI) or a timsTOF flex device (MALDI). CV measurements were carried out with a Metrohm Autolab PGSTAT 204 potentiostate/galvanostate and an Ag/AgCl reference electrode, Pt rod counter electrode and glassy carbon working electrode. All voltammograms were recorded at room temperature. CH_2Cl_2 was used as solvent for the individual compounds (c = 10^{-3} M), whereas nBu$_4$N(PF$_6$) (electrochemical grade (≥99.0%), Fluka) was employed as supporting electrolyte (c = 0.1 M). The potentials are given vs. the redox reference pair ferrocenium/ferrocene (Fc^+/Fc), measured at $E_{1/2}$ = 0.456 V in CH_2Cl_2.

X-band EPR spectra were measured at a magnettech MiniScope MS400 spectrometer (room temperature measurements, ~9.44 GHz) or a BRUKER Elexsys E500 EPR (low-temperature measurements, ~9.63 GHz) with an ER 4116DM CW dual mode resonator. The samples were measured either in NMR (room temperature) or EPR (low temperature) tubes. For the low temperature measurements the sample solution was filled into a glass capillary inside the EPR tube, while the EPR tube was filled with pentane (for better heat conduction). Room temperature samples in ACN were also measured in a glass capillary inside an empty NMR tube. If not stated otherwise, the samples were measured at room temperature. The simulation of EPR spectra was performed with the MATLAB tool EasySpin 6.0.2.[3]

S1.2 X-ray crystallography

Suitable crystals for single-crystal structure determination were taken directly from the mother liquor, taken up in per-fluorinated polyether oil and fixed on a cryo loop. Full shells of intensity data were collected at low temperature with a Bruker D8 Venture, dual source (Mo-K□ radiation, microfocus X-ray tube, Photon III detector). Data were processed with the standard Bruker (SAINT, APEX3/4) software package.[4] Multiscan absorption correction was applied using the SADABS program.[5] The structures were solved by intrinsic phasing[6] and refined using the SHELXTL software package (Version 2018/3).[7a,7b,8,7c] Graphical handling of the structural data during solution and refinement were performed with OLEX2.[9] All non-hydrogen atoms were given anisotropic displacement parameters. Hydrogen atoms bound to carbon were input at calculated positions and refined with a riding model. Hydrogen atoms bound to nitrogen were located in difference Fourier syntheses and refined, either fully or with appropriate distance and/or symmetry. Split atom models were used to refine disordered groups and/or solvent molecules. When found necessary, suitable geometry and adp

restraints were applied.[10] Due to severe disorder and fractional occupancy, electron density attributed to the solvent of crystallization was removed from some of the structures with the BYPASS procedure,[11] as implemented in PLATON (squeeze/hybrid).[12]

Deposition Numbers 2482872 for $[PdCl_2(L1)]$, 2482875 for $[PdCl_2(L1)]PF_6$, 2482877 for $[Pd(Cl_2\text{-cat})(L1)]$, 2482873 for $[Pd(Cl_2\text{-cat})(L1)]PF_6$, 2482874 for $[Pd(Cl_4\text{-cat})(L1)]$, 2482880 for $[PdCl_2(L2)]$, 2482876 for $[Pd(tBu\text{-cat})(L2)](PF_6)_2$, 2482881 for $[Pd(Cl_2\text{-cat})(L2)]$, 2482878 for $[Pd(Cl_2\text{-cat})(L2)]PF_6$, 2482879 for $[Pd(Cl_4\text{-cat})(L2)]$, 2482933 for $[Pd(Cl_4\text{-cat})(L2)]PF_6$ contain the supplementary crystallographic data for this paper. These data are provided free of charge by the joint Cambridge Crystallographic Data Centre and Fachinformationszentrum Karlsruhe Access Structure service.

S1.3 Ab initio calculations

Molecular structures were optimised at the density functional theory (DFT) level, using the long-range corrected LC-ωPBE functional[13] and the D3 dispersion correction with Becke-Johnson damping.[14] All DFT computations employed the Turbomole program package (version 7.9)[15] with the density function implementation provided by libXC.[16] The def2-SVP basis set series[17] along with appropriate auxiliary basis sets for density fitting were used,[18] the exact exchange was computed by a semi-numerical approach.[19] Scalar relativistic effects of Pd were taken into account through a small-core ECP.[20] For all equilibrium structures second derivatives were computed analytically. Solvent effects were treated at the COSMO (conductor-like screening model) level of theory,[21] where for geometry optimisations two values for the relative permittivity were used, $\epsilon = 2$ for emulating a merely non-polar solvent, and $\epsilon = \infty$ for a strongly polar solvent. To enhance the convergence of geometry optimisation, an implementation based on the Gaussian charge model was employed.[22] More accurate estimates of the solvation effects were obtained by the dCOSMO-RS (direct COSMO for real solvents) method,[23] using parameter sets for cyclohexane, dichloromethane, and acetonitrile (for 298.15 K). Temperature effects were computed using the rigid-rotor-harmonic-oscillator model (translation and rotation cancel nearly completely for relative energies in the present study). To avoid artifacts, low-frequency vibrations were set to at least 50 cm^{-1} for computing the vibrational partition function (for the zero-point energy the actual values were used).[24]

Hyperfine coupling tensors at nitrogen centres were computed at the same level of theory,[25] using larger basis sets (def2-TZVPP), which were decontracted for nitrogen. For computing Pd hyperfine coupling tensors, we shifted to a scalar relativistic treatment (with collinear spins) using the X2C approach.[26] The x2c-TZVPPall-s basis sets[27] were used, again decontracted for the centre of interest (Pd). The g tensors were also computed at the latter level of theory. The spin-density was visualised by the TmoleX program (version 25).[28]

More accurate estimates of the relative energies were computed at the coupled-cluster level, using the Molpro program package.[29] We used the pair-natural-orbital based local coupled-cluster method with single and double excitations and perturbative non-iterative triple excitations, augmented by explicitly electron-pair-distance dependent geminal factors, in short PNO-LCCSD(T)-F12.[30] The implementation uses a partially spin-adapted approach. As basis set, the cc-pVDZ-F12 basis was used (which actually derives from a triple-zeta type basis, aug-cc-pVTZ, but uses at most d functions for main row elements),[8] for Pd, however, the aug-cc-pVTZ-PP basis[31] was used along with the ECP28MDF relativistic pseudopotential.[20] The corresponding optimised auxiliary basis sets for density fitting of the Fock operator ('JKFIT') and the correlation contributions ('MP2FIT') were used, the 'JKFIT' basis was also used as complementary auxiliars basis set (CABS) for approximating certain integrals within the F12 approach. The CABS is also used to compute a basis-set incompleteness correction for the Hartree-Fock reference energy.

The transition path between the two radical forms was explored by a linear synchronous transit path,[32] using the LC-wPBE-D3(BJ)/def2-SVP structures (COSMO, $\epsilon = 2$). To this end, the two structures were aligned such that the Eckart conditions [33] were fulfilled.

S2 Synthetic protocols

S2.1 Compounds with L1

<u>Synthesis of [PdCl$_2$(L1)]</u>

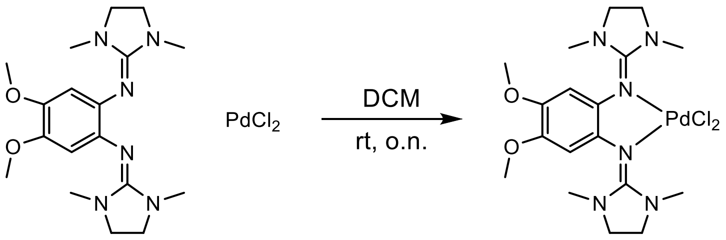

PdCl$_2$ (118 mg, 0.66 mmol, 1 eq.) and L1 (250 mg, 0.69 mmol, 1.05 eq.) were stirred in dichloromethane (40 mL) overnight. The solvent was removed under reduced pressure and the solid residue was washed with diethyl ether (40 mL). The product was dried under reduced pressure to yield a blue-green solid (95 %, 336 mg, 0.63 mmol).

^{1}H-NMR (600 MHz, CD$_2$Cl$_2$): δ = 5.94 (s, 2H, ^{Ar}H), 3.71 – 3.68 (m, 8H, CH$_2$), 3.67 (s, 6H, ^{O}CH$_3$), 3.08 (s, 12H, ^{N}CH$_3$).

^{13}C{^{1}H}-NMR (151 MHz, CD$_2$Cl$_2$): δ = 167.5 (2C, ^{G}C$_q$), 142.7 (2C, ^{OAr}C$_q$), 138.0 (2C, ^{NAr}C$_q$), 102.1 (2C, ^{Ar}CH), 57.5 (2C, ^{O}CH$_3$), 47.5 (2C, ^{G}CH$_2$), 34.7 (2C, ^{N}CH$_3$).

Elemental analysis calcd. (%) for $C_{18}H_{28}Cl_2N_6O_2Pd$ (537.78 g/mol): C 40.20, H 5.25, N 15.63; found 40.57, H 5.19, N 15.78.

MS (ESI+): m/z = calcd. for $[C_{18}H_{28}Cl_2N_6O_2Pd]^+$ 538.07, found 538.07.

CV (CH$_2$Cl$_2$, Ag/AgCl, ^{n}Bu$_4$NPF$_6$, scan rate 0.1 V/s, vs. Fc/Fc$^+$): $E_{1/2}$ = -0.36 (rev., -0.27/ -0.44), 0.21 (rev., 0.28/ 0.13) V.

UV-vis (CH$_2$Cl$_2$): λ (ε in M^{-1} cm^{-1}) =642 (871), 335 (8954), 300 (14456), 234 (45588) nm.

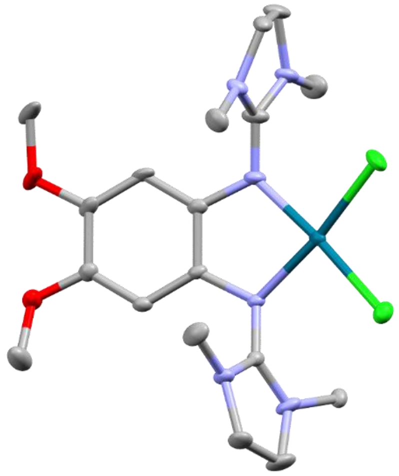

Figure S1: Solid-state structure of [PdCl$_2$(L1)]. Displacement ellipsoids drawn at the 50% probability level. Hydrogen atoms and solvent molecules are omitted. Colour code: grey: Carbon, red: Oxygen, light blue: Nitrogen, green: Chloride, blue: Palladium.

Synthesis of $[PdCl_2(L1)]PF_6$

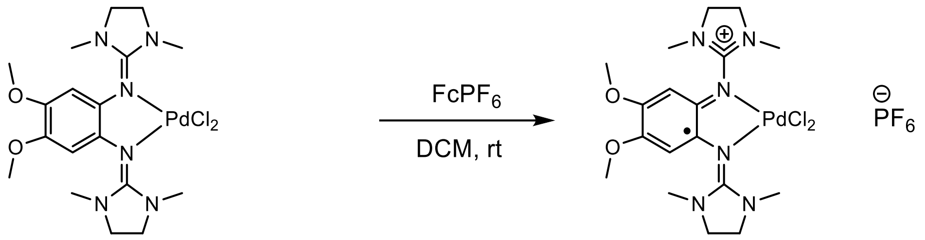

$[PdCl_2(L1)]$ (19.0 mg, 35.3 µmol, 1 eq.) and ferrocenium hexafluorophosphate (11.7 mg, 35.3 µmol, 1 eq.) were dissolved in DCM (3 mL). The mixture was stirred at room temperature for 1 h while turning from green-blue to a violet colour. After removal of the solvent under reduced pressure, the solid residue was washed with diethyl ether, until the ether remained colorless. After drying the solid *in vacuo,* the product was obtained as a violet solid (90%, 21.6 mg, 31.6 µmol).

Elemental analysis calcd. (%) for $C_{18}H_{28}Cl_2F_6N_6O_2PPd$ (682.75 g/mol): C 31.67, H 4.13, N 12.31; found 31.82, H 4.14, N 12.44.

MS (ESI+): m/z = calcd. for $[C_{18}H_{28}Cl_2N_6O_2Pd]^+$-$PF_6$ 538.07, found 538.0669.

UV-vis (CH_2Cl_2): λ (ε in M^{-1} cm^{-1}) =848 (325), 757 (378), 561 (6428), 525 (4502), 385 (4229), 336 (9554) nm.

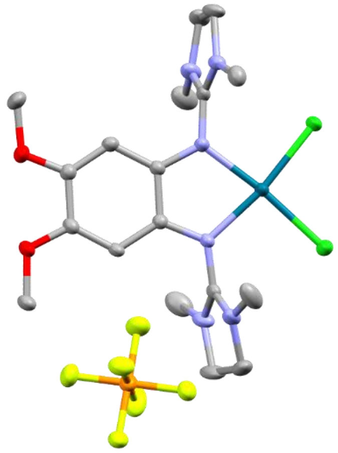

Figure S2: Solid-state structure of $[PdCl_2(L1)]PF_6$. Displacement ellipsoids drawn at the 50% probability level. Hydrogen atoms, solvent molecules and a second molecule are omitted. Colour code: grey: Carbon, red: Oxygen, light blue: Nitrogen, green: Chloride, blue: Palladium, orange: Phosphorus, yellow: Fluorine.

Synthesis of [Pd(*t*Bu-cat)(L1)]

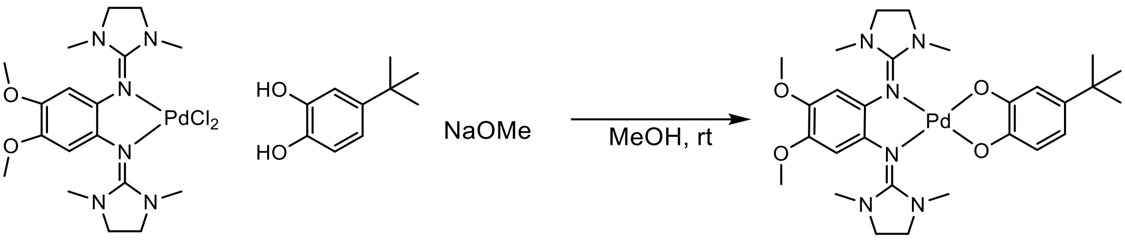

A catecholate solution was prepared by addition of NaOMe solution in MeOH (0.25 M, 2 eq.) to solid *t*Bu-cat (1 eq.) and used instantly in the following reaction.

[$PdCl_2$(L1)] (30 mg, 0.06 mmol, 1 eq.) was suspended in MeOH (3 mL) and the catecholate solution (0.45 mL, 0.25 M, 1 eq.) was added dropwise. After stirring for 15 minutes at room temperature, the solvent was removed under reduced pressure. The solid residue was dissolved in DCM (10 mL) and the solution was filtrated via a syringe filter. About 90% o the solvent was removed under reduced pressure and the product was precipitated by addition of pentane (15 mL). After filtration and drying the solid *in vacuo* the product was obtained as a greenish powder (38 %, 14 mg, 0.02 mmol).

^{1}H-NMR (600 MHz, CD_2Cl_2): δ = 6.20 (d, J = 2.3 Hz, 1H, ^{Cat}H), 6.18 - 6.14 (m, 2H, ^{Ar}H), 6.11 ((dd, J = 8.1 Hz, 2.3 Hz, 1H, ^{Cat}H), 6.04 (d, J = 8.1 Hz, 1H, ^{Cat}H), 3.77 – 3.64 (m, 8H CH_2), 3.70 (s, 6H, $^{O}CH_3$), 3.06 – 3.03 (m, 12H, $^{N}CH_3$), 1.19 (s, 9H, $^{tBu}CH_3$).

^{13}C{^{1}H}-NMR (151 MHz, CD_2Cl_2): δ = 165.1 ($^{G}C_q$), 164.1 ($^{CatO}C_q$), 162.3 ($^{CatO}C_q$), 143.1 ($^{OAr}C_q$), 137.4 ($^{CatC}C_q$), 137.0 ($^{NAr}C_q$), 111.8 (^{Cat}CH), 111.3 (^{Cat}CH), 110.06 (^{Cat}CH), 103.9 (^{Ar}CH), 57.2 ($^{O}CH_3$), 48.2 ($^{G}CH_2$), 35.7 ($^{N}CH_3$), 33.7 ($^{tBu}C_q$), 32.2 ($^{tBu}CH_3$).

Elemental analysis calcd. (%) for $C_{28}H_{40}N_6O_4Pd \cdot 1/2\ CH_2Cl_2$ (631.09 g/mol): C 50.82, H 6.14, N 12.48; found 50.95, H 7.03, N 12.15.

MS (ESI+): m/z = calcd. for $[C_{28}H_{40}N_6O_4Pd]^+$ 630.21, found 630.21.

CV (CH_2Cl_2, Ag/AgCl, $^{n}Bu_4NPF_6$, scan rate 0.1 V/s, vs. Fc/Fc^+): $E_{1/2}$ = -0.55 (rev., -0.49/ -0.60), -0.14 (rev., -0.09/ -0.19), 0.43 (rev., 0.48/ 0.37) V.

UV-vis (CH_2Cl_2): λ (ε in M^{-1} cm^{-1}) = 360 (8570), 320 (17900) nm.

Synthesis of [Pd(*t*Bu-cat)(L1)]PF_6

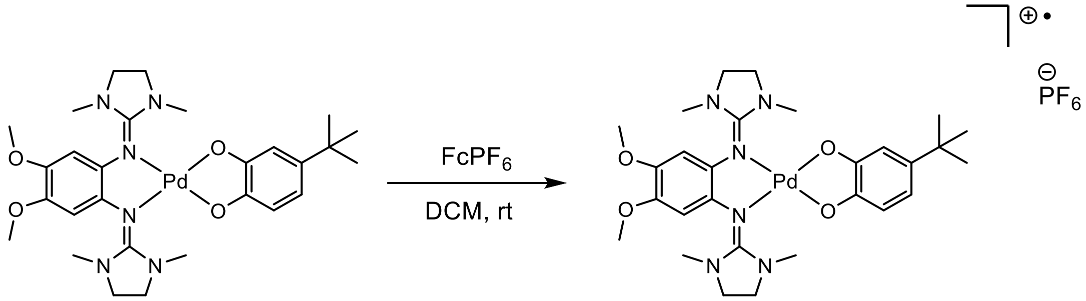

[Pd(*t*Bu-cat)(L1)] (10.0 mg, 15.9 µmol, 1 eq.) and ferrocenium hexafluorophosphate (5.0 mg, 15.1 µmol, 1 eq.) were dissolved in DCM (10 mL). The green mixture was stirred at room temperature for 1 h. After removal of the solvent under reduced pressure, the solid residue was washed with diethyl ether, until the ether remained colorless. After drying the solid *in vacuo,* the product was obtained as a green solid (98%, 12.0 mg, 15.5 µmol).

Elemental analysis calcd. (%) for $C_{28}H_{40}F_6N_6O_4PPd\cdot$ 1/2 CH_2Cl_2 (776.05 g/mol): C 41.82, H 5.05, N 10.27; found 42.29, H 5.09, N 9.62.

MS (MALDI+): m/z = calcd. for $[C_{28}H_{40}N_6O_4Pd]^+$-PF_6 630.21, found 630.2137.

UV-vis (CH_2Cl_2): λ (ε in M^{-1} cm^{-1}) = 1110 (2760), 570 (1539), 445 (2832), 342 (13300), 297 (21900) nm.

UV-vis (CH_3CN): λ (ε in M^{-1} cm^{-1}) = 1169 (4499), 584 (2316), 443 (4441), 335 (30311), 288 (43925) nm.

Synthesis of [Pd(Cl_2-cat)(L1)]

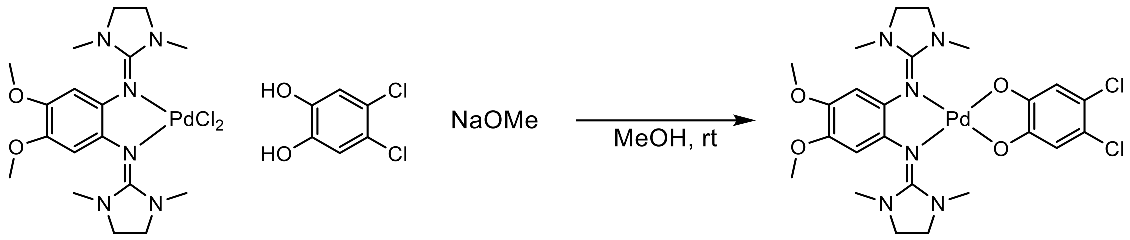

A catecholate solution was prepared by addition of NaOMe solution in MeOH (0.54 M, 2 eq.) to solid Cl_2-cat (1 eq.) and used instantly in the following reaction.

[$PdCl_2$(L1)] (71 mg, 0.13 mmol, 1 eq.) was suspended in MeOH (10 mL) and the catecholate solution (0.50 mL, 0.54 M, 1 eq.) was added dropwise. After stirring for 1 h at room temperature, the solvent was removed under reduced pressure. The solid residue was dissolved in DCM (5 mL) and the solution was filtrated via a syringe filter. The solvent was removed under reduced pressure and the solid washed with diethyl ether (10 mL). After drying *in vacuo* the product was obtained as a greenish powder (39 %, 33 mg, 0.05 mmol).

^{1}H-NMR (600 MHz, CD_2Cl_2): δ = 6.22 (s, 2H, ^{Ar}H), 6.13 (s, 2H, ^{Ar}H), 3.69 (s, 6H, $^{O}CH_3$), 3.68 – 3.64 (m, 8H, CH_2), 3.00 (s, 12H, $^{N}CH_3$).

^{13}C{^{1}H}-NMR (151 MHz, CD_2Cl_2): δ = 165.04 (2C, $^{CatO}C_q$), 164.83 (2C, $^{G}C_q$), 143.28 (2C, $^{OAr}C_q$), 136.75 (2C, $^{NAr}C_q$), 114.95 (2C, $^{CatCl}C_q$), 113.11 (2C, ^{Ar}CH), 103.69 (2C, ^{Ar}CH), 57.17 (2C, $^{O}CH_3$), 48.15 (2C, $^{G}CH_2$), 35.58 (2C, $^{N}CH_3$). One quartiary carbon of the catecholate is not observed.

Elemental analysis calcd. (%) for $C_{24}H_{30}Cl_2N_6O_4Pd \cdot 1/2\ CH_2Cl_2$ (686.33 g/mol): C 42.88, H 4.55, N 12.25; found 42.74, H 4.74, N 12.33.

MS (ESI+): m/z = calcd. for $[C_{24}H_{30}Cl_2N_6O_4Pd]^+$ 644.07, found 644.07.

CV (CH_2Cl_2, Ag/AgCl, $^{n}Bu_4NPF_6$, scan rate 0.1 V/s, vs. Fc/Fc$^+$): $E_{1/2}$ = -0.31 (rev., -0.25/ -0.37), 0.03 (rev., 0.09/ -0.03), 0.53 (rev., 0.58/ 0.48) V.

UV-vis (CH_2Cl_2): λ (ε in M^{-1} cm^{-1}) =573 (3570), 406 (4430), 337 (18700), 230 (74900) nm.

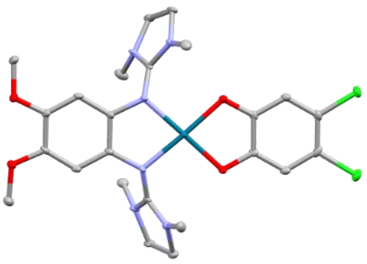

Figure S3: Solid-state structure of [Pd(Cl_2-cat)(L1)]. Displacement ellipsoids drawn at the 50% probability level. Hydrogen atoms, solvent molecules and a second molecule are omitted. The two molecules in the asymmetric unit do not differ significantly in their structural parameters. Colour code: grey: Carbon, red: Oxygen, light blue: Nitrogen, green: Chloride, blue: Palladium.

Synthesis of [Pd(Cl$_2$-cat)(L1)]PF$_6$

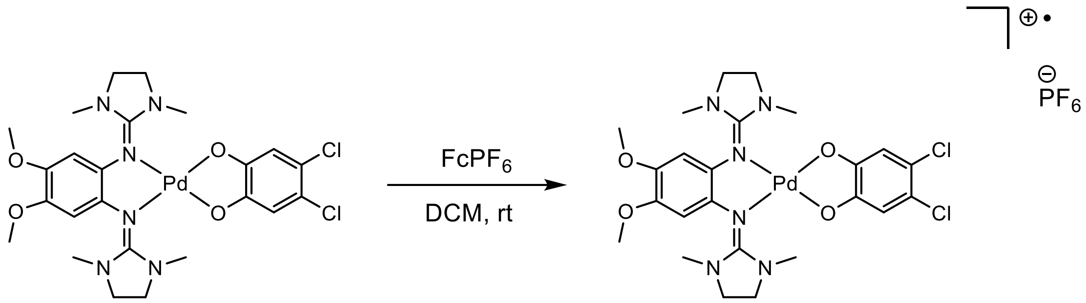

[Pd(Cl$_2$-cat)(L1)] (10.0 mg, 15.5 µmol, 1 eq.) and ferrocenium hexafluorophosphate (5.1 mg, 15.5 µmol, 1 eq.) were dissolved in DCM (3 mL). The mixture was stirred at room temperature for 1 h while turning from green to violet. After removal of the solvent under reduced pressure, the solid residue was washed with diethyl ether, until the ether remained colorless. After drying the solid *in vacuo,* the product was obtained as a purple solid (75%, 9.2 mg, 11.7 µmol).

Elemental analysis calcd. (%) for $C_{24}H_{30}F_6Cl_2N_6O_4PPd \cdot 1/3\ CH_2Cl_2$ (788.83 g/mol): C 35.77, H 3.78, N 10.28; found 35.8, H 4.29, N 10.91.

MS (MALDI+): m/z = calcd. for $[C_{24}H_{30}Cl_2N_6O_4Pd]^+$ 644.07, found 644.0721.

UV-vis (CH_2Cl_2): λ (ε in M^{-1} cm^{-1}) = 1940 (652), 1365 (493), 513 (454), 393 (703), 318 (2047) nm.

UV-vis (CH_3CN): λ (ε in M^{-1} cm^{-1}) = 1118 (382), 499 (533), 384 (1164), 328 (2608) nm.

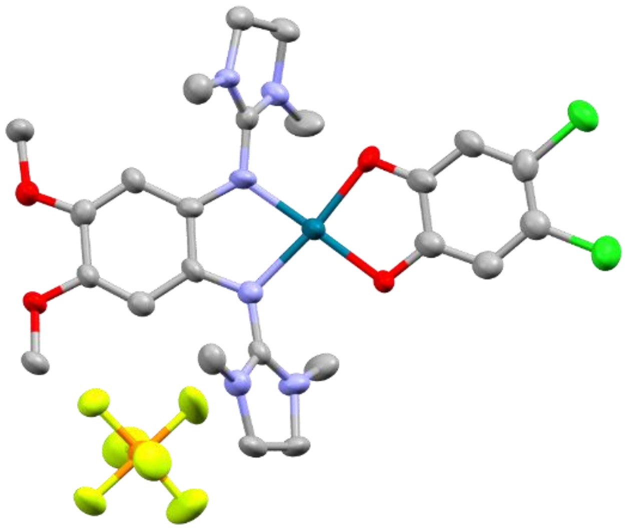

Figure S4: Solid-state structure of [Pd(Cl$_2$-cat)(L1)]PF$_6$. Displacement ellipsoids drawn at the 50% probability level. Hydrogen atoms and solvent molecules are omitted. Colour code: grey: Carbon, red: Oxygen, light blue: Nitrogen, green: Chloride, blue: Palladium, orange: Phosphorus, yellow: Fluorine.

Synthesis of [Pd(Cl_4-cat)(L1)]PF_6

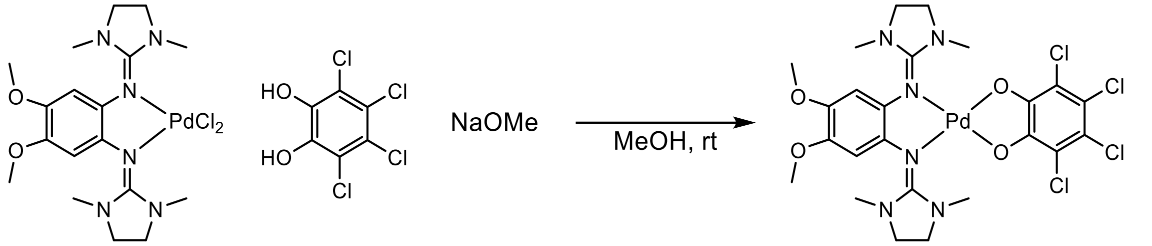

A catecholate solution was prepared by addition of NaOMe solution in MeOH (0.54 M, 2 eq.) to solid Cl_4-cat (1 eq.) and used instantly in the following reaction.

[$PdCl_2$(L1)] (45 mg, 0.08 mmol, 1 eq.) was suspended in MeOH (5 mL) and the catecholate solution (0.31 mL, 0.54 M, 1 eq.) was added dropwise. After stirring for 1 h at room temperature, the solvent was removed under reduced pressure. The solid residue was dissolved in DCM (5 mL) and the solution was filtrated via a syringe filter. The solvent was removed under reduced pressure and the solid washed with diethyl ether (10 mL). After drying *in vacuo* the product was obtained as a greenish powder (38 %, 23 mg, 0.03 mmol).

^{1}H-NMR (600 MHz, CD_2Cl_2): δ = 6.14 (s, 2H, ^{Ar}H), 3.70 (s, 6H, $^{O}CH_3$), 3.72 – 3.69 (m, 8H, CH_2), 3.04 (s, 12H, $^{N}CH_3$).

^{13}C{^{1}H}-NMR (151 MHz, CD_2Cl_2): δ = 165.56 (2C, $^{G}C_q$), 160.43 (2C, $^{CatO}C_q$), 143.51 (2C, $^{OAr/NAr}C_q$), 136.92 (2C, $^{Nar/OAr}C_q$), 115.06 (2C, $^{CatCl}C_q$), 114.94 (2C, $^{CatCl}C_q$), 103.75 (2C, ^{Ar}CH), 57.22 (2C, $^{O}CH_3$), 48.19 (2C, $^{G}CH_2$), 35.61 (2C, $^{N}CH_3$).

Elemental analysis calcd. (%) for $C_{24}H_{28}Cl_4N_6O_4Pd\cdot 1.5\ C_5H_{12}$ (712.75 g/mol): C 46.09, H 5.65, N 10.24; found 46.32, H 6.45, N 10.41.

MS (ESI+): m/z = calcd. for $[C_{24}H_{28}Cl_4N_6O_4Pd]^+$ 713.99, found 713.99.

CV (CH_2Cl_2, Ag/AgCl, $^{n}Bu_4NPF_6$, scan rate 0.1 V/s, vs. Fc/Fc^+): $E_{1/2}$ = -0.26 (rev., -0.16/ -0.36), 0.24 (rev., 0.28/ 0.20), 0.70 (rev., 0.73/ 0.67) V.

UV-vis (CH_2Cl_2): λ (ε in M^{-1} cm^{-1}) =565 (536), 311 (12800), 272 (13800), 234 (57500) nm.

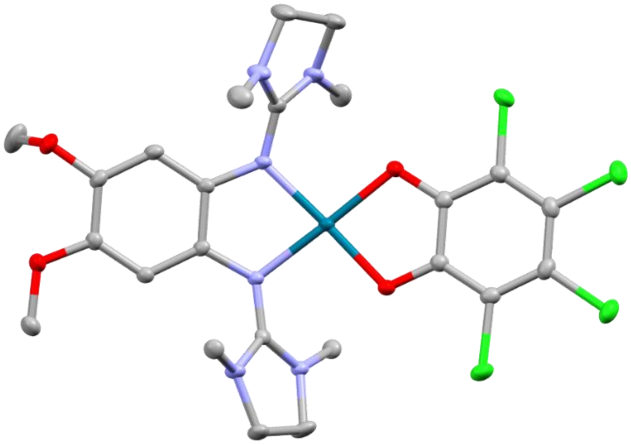

Figure S5: Solid-state structure of [Pd(Cl_4-cat)(L1)]. Displacement ellipsoids drawn at the 50% probability level. Hydrogen atoms and solvent molecules are omitted. Colour code: grey: Carbon, red: Oxygen, light blue: Nitrogen, green: Chloride, blue: Palladium.

S2.2 Compounds with L2

Synthesis of [$PdCl_2$(L2)]

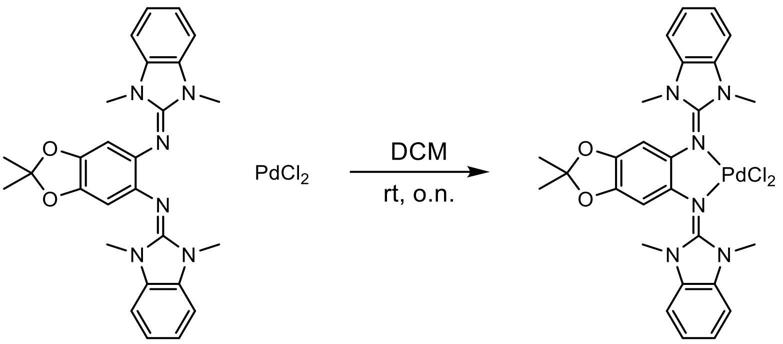

$PdCl_2$ (52 mg, 0.29 mmol, 1 eq.) and L2 (145 mg, 0.31 mmol, 1.05 eq.) were stirred in dichloromethane (8 mL) overnight. The solvent was removed under reduced pressure and the solid residue was washed with diethyl ether (10 mL). The product was dried under reduced pressure to yield a blue-green solid (70 %, 134 mg, 0.21 mmol).

^{1}H-NMR (600 MHz, CD_2Cl_2): δ = 7.46 (s, 8H, ^{Ar}CH), 5.69 (s, 2H, ^{Ar}H), 3.87 (s, 12H, $^{N}CH_3$), 1.54 (s, 6H, $^{C}CH_3$).

^{13}C{^{1}H}-NMR (151 MHz, CD_2Cl_2): δ = 156.2 (2C, $^{G}C_q$), 141.0 (2C, $^{OAr}C_q$), 138.5 (2C, $^{NAr}C_q$), 130.9 (4C, $^{NAr}C_q$), 124.9 (4C, ^{Ar}CH), 118.1 (1C, $^{CH3C}C_q$), 111.1 (4C, ^{Ar}CH), 96.2 (2C, ^{Ar}CH), 31.7 (4C, $^{N}CH_3$), 25.7 (2C, $^{C}CH_3$).

Elemental analysis calcd. (%) for $C_{27}H_{28}Cl_2N_6O_2Pd \cdot 1/3\ CH_2Cl_2$ (645.88 g/mol): C 48.70, H 4.29, N 12.47; found 48.89, H 4.53, N 13.18.

MS (MALDI+): m/z = calcd. for $[C_{27}H_{28}Cl_2N_6O_2Pd]^+$ 646.07, found 646.07.

CV (CH_2Cl_2, Ag/AgCl, $^{n}Bu_4NPF_6$, scan rate 0.1 V/s, vs. Fc/Fc^+): $E_{1/2}$ = -0.30 (rev., -0.26/ -0.34), 0.25 (rev., 0.29/ 0.20) V.

UV-vis (CH_2Cl_2): λ (ε in M^{-1} cm^{-1}) =656 (873), 388 (4688), 336 (8267), 292 (10296), 265 (8917), 228 (23381) nm.

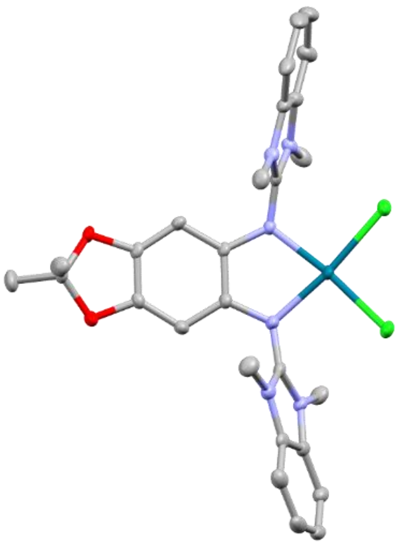

Figure S 6: Solid-state structure of [$PdCl_2$(L2)]. Displacement ellipsoids drawn at the 50% probability level. Hydrogen atoms and solvent molecules are omitted. Colour code: grey: Carbon, red: Oxygen, light blue: Nitrogen, green: Chloride, blue: Palladium.

Synthesis of [Pd(*t*Bu-cat)(L2)]

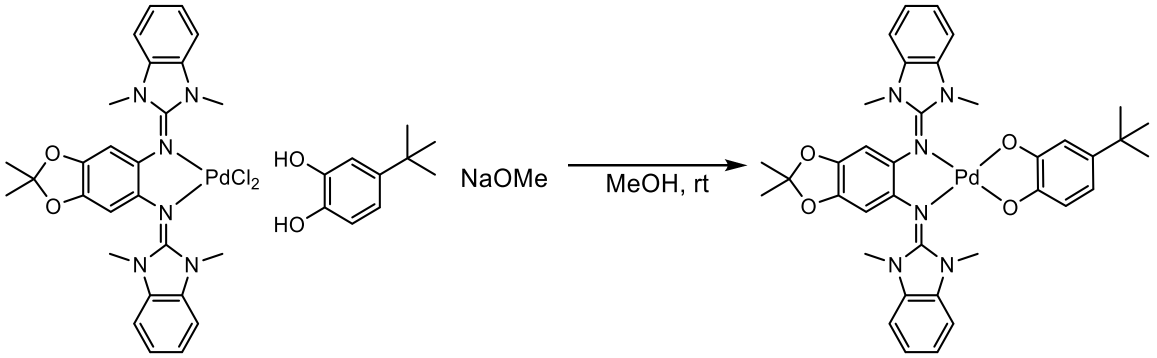

A catecholate solution was prepared by addition of NaOMe solution in MeOH (0.25 M, 2 eq.) to solid *t*Bu-cat (1 eq.) and used instantly in the following reaction.

[$PdCl_2$(L2)] (36 mg, 0.06 mmol, 1 eq.) was suspended in MeOH (3 mL) and the catecholate solution (0.45 mL, 0.25 M, 1 eq.) was added dropwise. After stirring for 15 minutes at room temperature, the solvent was removed under reduced pressure. The solid residue was dissolved in DCM (10 mL) and the solution was filtrated via a syringe filter. About 90% o the solvent was removed under reduced pressure and the product was precipitated by addition of pentane (15 mL). After filtration and drying the solid *in vacuo* the product was obtained as a greenish powder (37 %, 15 mg, 0.02 mmol).

^{1}H-NMR (600 MHz, CD_2Cl_2): δ = 7.44 – 7.34 (m, 8H, ^{Ar}H), 6.08 (d, J = 2.3 Hz, 1H, ^{Cat}H), 6.04 (dd, J = 8.1 Hz, 2.3 Hz, 1H, ^{Cat}H), 6.00 – 5.94 (m, 2H, ^{Ar}H), 5.90 (d, J = 8.0 Hz, 1H, ^{Cat}H), 3.84 – 3.79 (m, 12H, $^{N}CH_3$), 1.58 (s, 6H, $^{C}CH_3$), 1.11 (s, 9H, $^{tBu}CH_3$).

^{13}C{^{1}H}-NMR (151 MHz, CD_2Cl_2): δ = 163.4 ($^{CatO}C_q$), 161.5 ($^{CatO}C_q$), 154.7 ($^{G}C_q$), 141.6 ($^{OAr}C_q$), 137.3 ($^{NAr}C_q$), 137.2 ($^{CatC}C_q$), 131.8 ($^{NAr}C_q$), 124.2 (^{Ar}CH), 111.8 (^{Cat}CH), 111.3 (^{Cat}CH), 110.6 (^{Cat}CH), 110.5 (^{Ar}CH), 97.9 (^{Ar}CH), 33.4 ($^{tBu}C_q$), 32.3 ($^{N}CH_3$), 31.7 ($^{tBu}CH_3$), 25.7 ($^{C}CH_3$).

Elemental analysis calcd. (%) for $C_{37}H_{40}N_6O_4Pd \cdot 1/2\ CH_2Cl_2$ (739.19 g/mol): C 57.62, H 5.29, N 10.75; found 57.65, H 5.77, N 10.32.

MS (ESI+): m/z = calcd. for $[C_{37}H_{40}N_6O_4Pd]^+$ 738.2146, found 738.2149.

CV (CH_2Cl_2, Ag/AgCl, $^{n}Bu_4NPF_6$, scan rate 0.1 V/s, vs. Fc/Fc$^+$): $E_{1/2}$ = -0.51 (rev., -0.45/ -0.58), -0.07 (rev., 0.00/ -0.14), 0.52 (rev., 0.57/ 0.46) V.

UV-vis (CH_2Cl_2): λ (ε in M^{-1} cm^{-1}) = 401 (11400), 349 (19700), 303 (25400) nm.

Synthesis of [Pd(*t*Bu-cat)(L2)]PF_6

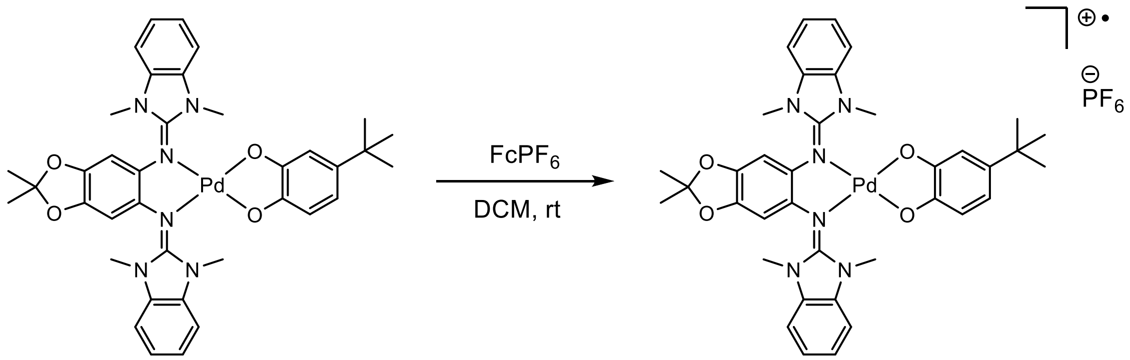

[Pd(*t*Bu-cat)(L2)] (3.0 mg, 4.1 µmol, 1 eq.) and ferrocenium hexafluorophosphate (1.3 mg, 3.9 µmol, 1 eq.) were dissolved in DCM (5 mL). The green mixture was stirred at room temperature for 1 h. After removal of the solvent under reduced pressure, the solid residue was washed with diethyl ether, until the ether remained colorless. After drying the solid *in vacuo,* the product was obtained as a green solid (96%, 3.0 mg, 4.1 µmol).

Elemental analysis calcd. (%) for $C_{37}H_{40}F_6N_6O_4PPd \cdot 2\ CH_2Cl_2$ (884.15 g/mol): C 44.44, H 4.21, N 7.97; found 44.68, H 4.41, N 7.69.

MS (ESI+): m/z = calcd. for $[C_{37}H_{40}N_6O_4Pd]^+$-PF_6: 738.2146, found 738.2148.

UV-vis (CH_2Cl_2): λ (ε in M^{-1} cm^{-1}) = 1200 (1181), 597 (1506), 384 (7710), 320 (15600) nm.

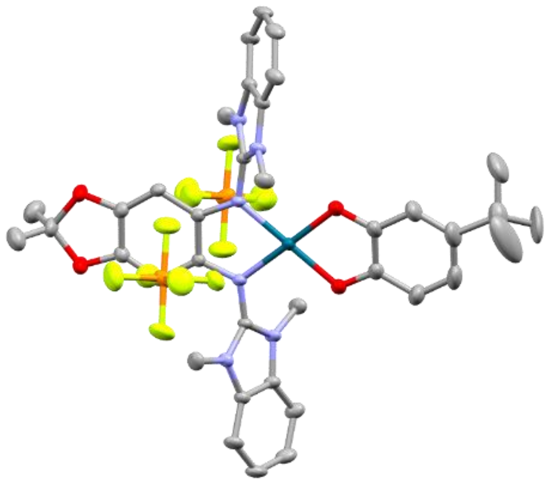

Figure S7: Solid-state structure of the twofold oxidised complex [Pd(*t*Bu-cat)(L2)]$(PF_6)_2$. The complex crystallized from a separate experiment using $FcPF_6$ in excess. Displacement ellipsoids drawn at the 50% probability level. Hydrogen atoms and solvent molecules are omitted. Colour code: grey: Carbon, red: Oxygen, light blue: Nitrogen, green: Chloride, blue: Palladium, orange: Phosphorus, yellow: Fluorine.

Synthesis of [Pd(Cl_2-cat)(L2)]

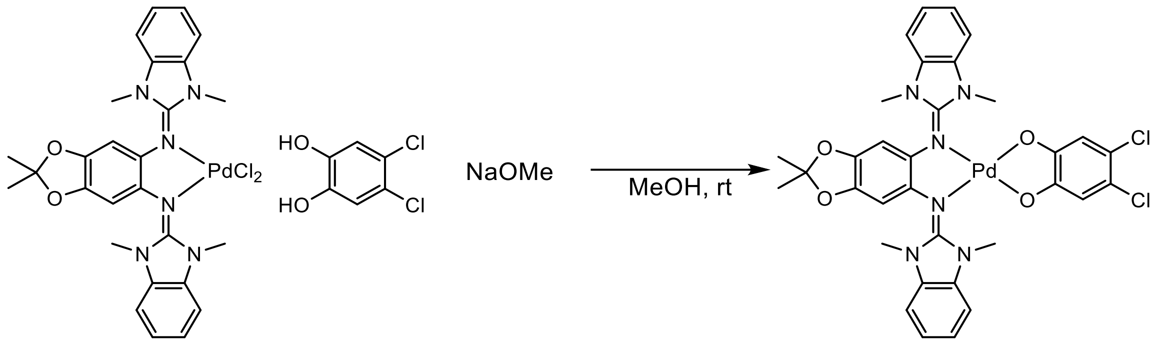

A catecholate solution was prepared by addition of NaOMe solution in MeOH (0.25 M, 2 eq.) to solid Cl_2-cat (1 eq.) and used instantly in the following reaction.

[$PdCl_2$(L2)] (65 mg, 0.10 mmol, 1 eq.) was suspended in MeOH (10 mL) and the catecholate solution (0.89 mL, 0.25 M, 1.1 eq.) was added dropwise. After stirring for 1h at room temperature, the solvent was removed under reduced pressure. The solid residue was dissolved in DCM (15 mL) and the solution was filtrated via a syringe filter. About 90% of the solvent was removed under reduced pressure and the product was precipitated by addition of pentane (15 mL). After filtration and drying the solid *in vacuo* the product was obtained as a greenish powder (75 %, 57 mg, 0.08 mmol).

^{1}H-NMR (600 MHz, CD_2Cl_2): δ = 7.43 – 7.38 (m, 8H, ^{Ar}H), 6.04 (s, 2H, ^{Cat}H), 5.97 (s, 2H, ^{Ar}H), 3.79 (s, 12H, $^{N}CH_3$), 1.58 (s, 6H, $^{C}CH_3$).

^{13}C{^{1}H}-NMR (151 MHz, CD_2Cl_2): δ = 164.8 ($^{CatO}C_q$), 154.5 ($^{G}C_q$), 141.8 ($^{OAr}C_q$), 137.2 ($^{NAr}C_q$), 131.7 ($^{NAr}C_q$), 124.5 (^{Ar}CH), 118.4 ($^{C}C_q$), 114.9 ($^{Cl}C_q$), 113.2 (^{Ar}CH), 110.6 (^{Ar}CH), 97.9 (^{Ar}CH), 32.3 ($^{N}CH_3$), 25.7 ($^{C}CH_3$).

Elemental analysis calcd. (%) for $C_{33}H_{30}Cl_2N_6O_4Pd$ · Et_2O · MeOH (751.96 g/mol): C 53.19, H 5.17, N 9.79; found 53.15, H 5.40, N 9.06.

MS (MALDI+): m/z = calcd. for $[C_{33}H_{30}Cl_2N_6O_4Pd]^+$ 752.07, found 752.0721.

CV (CH_2Cl_2, Ag/AgCl, $^{n}Bu_4NPF_6$, scan rate 0.1 V/s, vs. Fc/Fc$^+$): $E_{1/2}$ = -0.25 (rev., -0.18/ -0.32), 0.06 (rev., 0.14/ -0.02), 0.61 (rev., 0.69/ 0.54) V.

UV-vis (CH_2Cl_2): λ (ε in M^{-1} cm^{-1}) = 597 (1190), 385 (8950), 338 (14300), 298 (11100), 228 (52300) nm.

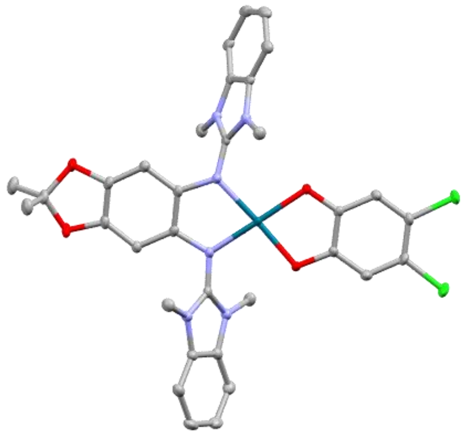

Figure S8: Solid-state structure of [Pd(Cl_2-cat)(L2)]. Displacement ellipsoids drawn at the 50% probability level. Hydrogen atoms, cocrystallized catechole, the second molecule of [Pd(Cl_2-cat)(L2)] and solvent molecules are omitted. Colour code: grey: Carbon, red: Oxygen, light blue: Nitrogen, green: Chloride, blue: Palladium.

Synthesis of [Pd(Cl_2-cat)(L2)]PF_6

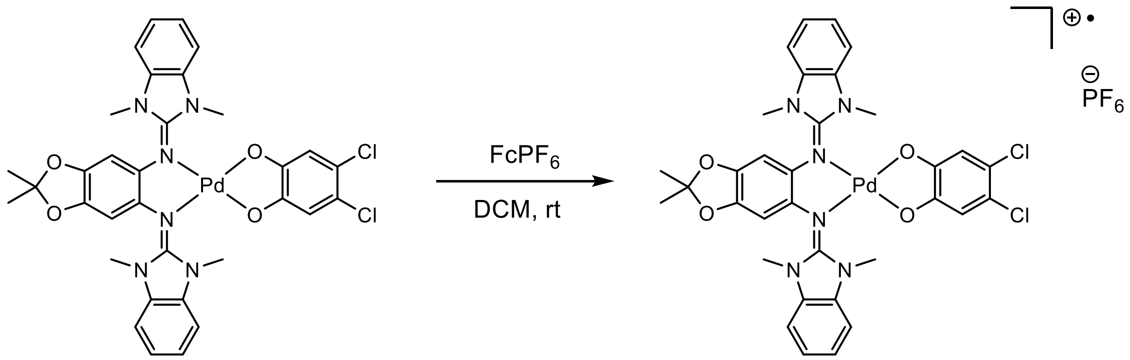

[Pd(Cl_2-cat)(L2)] (28.5 mg, 37.9 µmol, 1 eq.) and ferrocenium hexafluorophosphate (12.5 mg, 39.7 µmol, 1 eq.) were dissolved in DCM (10 mL). The mixture was stirred at room temperature for 1 h while turning violet. After removal of the solvent under reduced pressure, the solid residue was washed with diethyl ether, until the ether remained colorless. After drying the solid *in vacuo,* the product was obtained as a purple solid (79%, 28.0 mg, 29.8 µmol).

Elemental analysis calcd. (%) for $C_{33}H_{30}Cl_2F_6N_6O_4PdP \cdot 1/2\ CH_2Cl_2$ (896.93 g/mol): C 41.59, H 3.29, N 11.61; found 41.53, H 3.43, N 8.01.

MS (MALDI+): m/z = calcd. for $[C_{33}H_{30}Cl_2N_6O_4Pd]^+$-$PF_6$ 752.07, found 752.0738.

UV-vis (CH_2Cl_2): λ (ε in M^{-1} cm^{-1}) = 2018 (7256), 1381 (3288), 897 (1613), 551 (3874), 409 (8416), 325 (18751), 288 (18701) nm.

UV-vis (CH_3CN): λ (ε in M^{-1} cm^{-1}) = 1175 (2470), 882 (2344), 773 (1749), 695 (1301), 542 (3773), 388 (9317) nm.

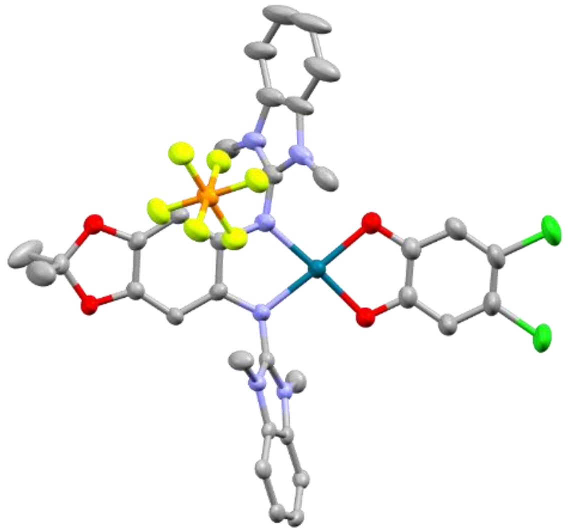

Figure S9: Solid-state structure of [Pd(Cl_2-cat)(L2)]PF_6. Displacement ellipsoids drawn at the 50% probability level. Hydrogen atoms, the second molecule of [Pd(Cl_2-cat)(L2)] and solvent molecules are omitted. Colour code: grey: Carbon, red: Oxygen, light blue: Nitrogen, green: Chloride, blue: Palladium, orange: Phosphorus, yellow: Fluorine.

Synthesis of [Pd(Cl_4-cat)(L2)]

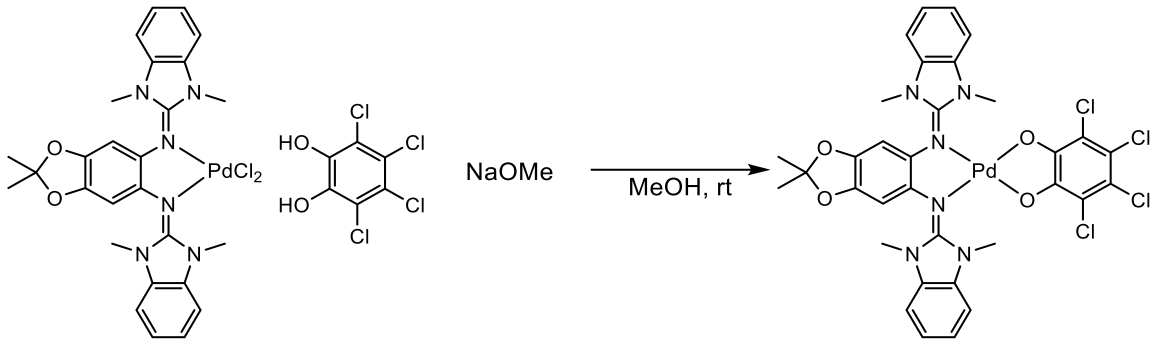

A catecholate solution was prepared by addition of NaOMe solution in MeOH (0.54 M, 2 eq.) to solid Cl_4-cat (1 eq.) and used instantly in the following reaction.

[$PdCl_2$(L2)] (68 mg, 0.11 mmol, 1 eq.) was suspended in MeOH (10 mL) and the catecholate solution (0.39 mL, 0.54 M, 1 eq.) was added dropwise. After stirring for 1h at room temperature, the solvent was removed under reduced pressure. The solid residue was dissolved in DCM (15 mL) and the solution was filtrated via a syringe filter. About 90% of the solvent was removed under reduced pressure and the product was precipitated by addition of pentane (15 mL). After filtration and drying the solid *in vacuo* the product was obtained as a greenish powder (62 %, 54 mg, 0.07 mmol).

^{1}H-NMR (600 MHz, CD_2Cl_2): δ = 7.42 – 7.41 (m, 8H, ^{Ar}H), 6.02 (s, 2H, ^{Ar}H), 3.82 (s, 12H, $^{N}CH_3$), 1.60 (s, 6H, $^{C}CH_3$).

^{13}C{^{1}H}-NMR (151 MHz, CD_2Cl_2): δ = 160.0 ($^{CatO}C_q$), 154.4 ($^{G}C_q$), 142.0 ($^{OAr}C_q$), 137.3 ($^{NAr}C_q$), 131.8 ($^{NAr}C_q$), 124.7 (^{Ar}CH), 118.6 (^{C}Cq), 115.2 ($^{Cl}C_q$), 115.1 ($^{Cl}C_q$), 110.7 (^{Ar}CH), 97.3 (^{Ar}CH), 32.6 ($^{N}CH_3$), 25.7 ($^{C}CH_3$).

Elemental analysis calcd. (%) for $C_{33}H_{28}Cl_4N_6O_4Pd \cdot Et_2O$ (820.85 g/mol): C 49.66, H 4.28, N 9.39; found 50.27, H 4.65, N 9.58.

MS (MALDI+): m/z = calcd. for $[C_{33}H_{28}Cl_4N_6O_4Pd]^+$ 821.99, found 819.9949.

CV (CH_2Cl_2, Ag/AgCl, $^{n}Bu_4NPF_6$, scan rate 0.1 V/s, vs. Fc/Fc^+): $E_{1/2}$ = -0.21 (rev., -0.15/ -0.17), 0.20 (rev., 0.25/ 0.15), 0.68 (rev., 0.73/ 0.63) V.

UV-vis (CH_2Cl_2): λ (ε in M^{-1} cm^{-1}) = 601 (1420), 330 (19400), 229 (64000) nm.

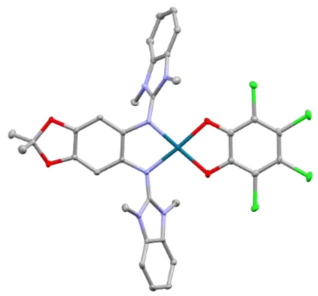

Figure S10: Solid-state structure of [Pd(Cl_4-cat)(L2)]. Displacement ellipsoids drawn at the 50% probability level. Hydrogen atoms and solvent molecules are omitted. Colour code: grey: Carbon, red: Oxygen, light blue: Nitrogen, green: Chloride, blue: Palladium.

Synthesis of [Pd(Cl_4-cat)(L2)]PF_6

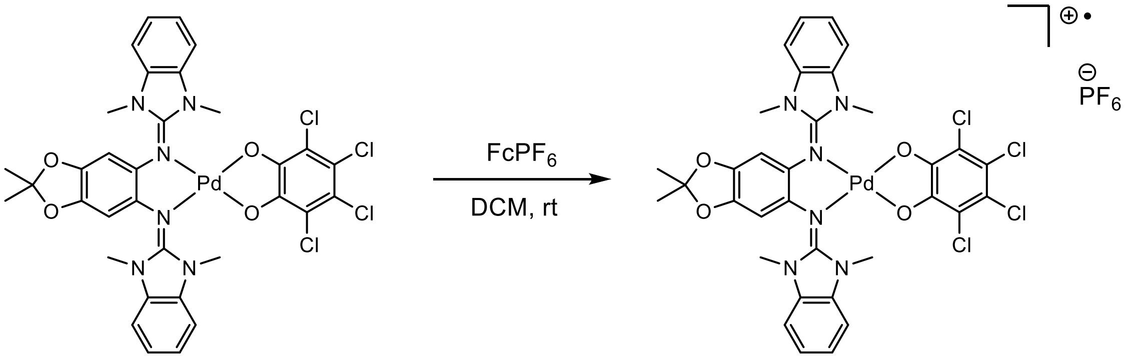

[Pd(Cl_4-cat)(L2)] (14.0 mg, 17.1 µmol, 1 eq.) and ferrocenium hexafluorophosphate (5.6 mg, 17.1 µmol, 1 eq.) were dissolved in DCM (3 mL). The mixture turned from green to purple while stirring at room temperature for 15 min. After removal of the solvent under reduced pressure, the solid residue was washed with diethyl ether, until the ether remained colorless. After drying the solid *in vacuo,* the purple product was obtained (72%, 11.9 mg, 12.3 µmol).

Elemental analysis calcd. (%) for $C_{33}H_{28}F_6Cl_4N_6O_4PPd$ (965.81 g/mol): C 41.04, H 2.92, N 8.70; found C 40.83, H 3.25, N 8.15.

MS (ESI+): m/z = calcd. for $[C_{33}H_{28}Cl_4N_6O_4Pd]^+$ -PF_6 819.99, found 819.9966.

UV-vis (CH_2Cl_2): λ (ε in M^{-1} cm^{-1}) = (1594 (3549), shoulder), 1253 (4142), 895 (3200), 547 (5193), 406 (12827) nm.

UV-vis (CH_3CN): λ (ε in M^{-1} cm^{-1}) = 1069 (3402), 882 (4483), 775 (3257), 529 (5100), 390 (15731) nm.

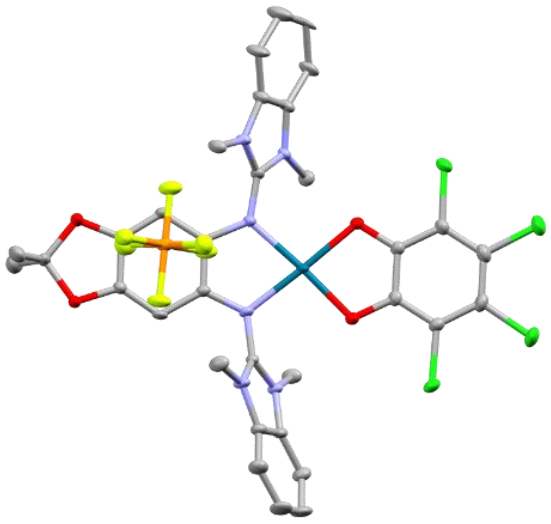

Figure S11: Solid-state structure of [Pd(Cl_4-cat)(L2)]PF_6. Displacement ellipsoids drawn at the 50% probability level. Hydrogen atoms and solvent molecules are omitted. Colour code: grey: Carbon, red: Oxygen, light blue: Nitrogen, green: Chloride, blue: Palladium, orange: Phosphorus, yellow: Fluorine.

S3 NMR spectroscopy

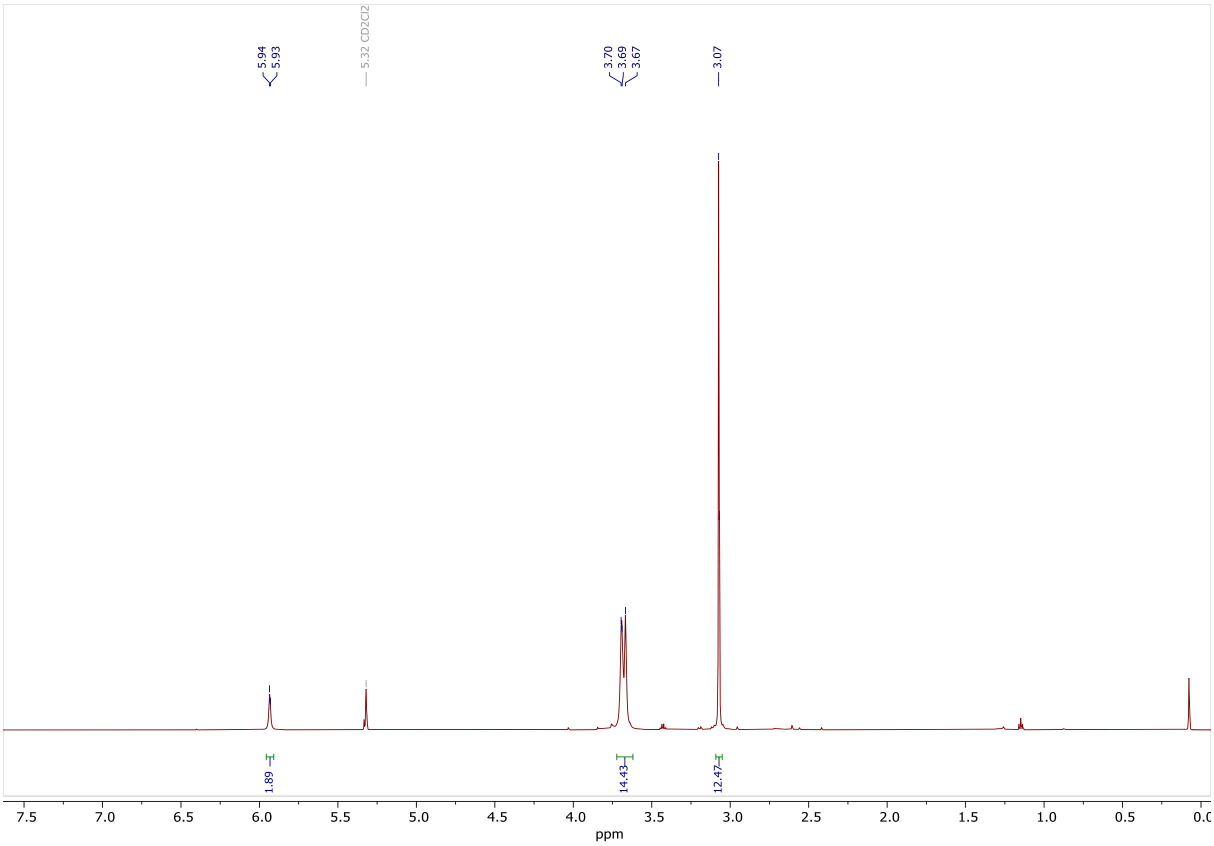

Figure S12: ^{1}H NMR spectrum (600 MHz, CD_2Cl_2) of $[PdCl_2(L1)]$.

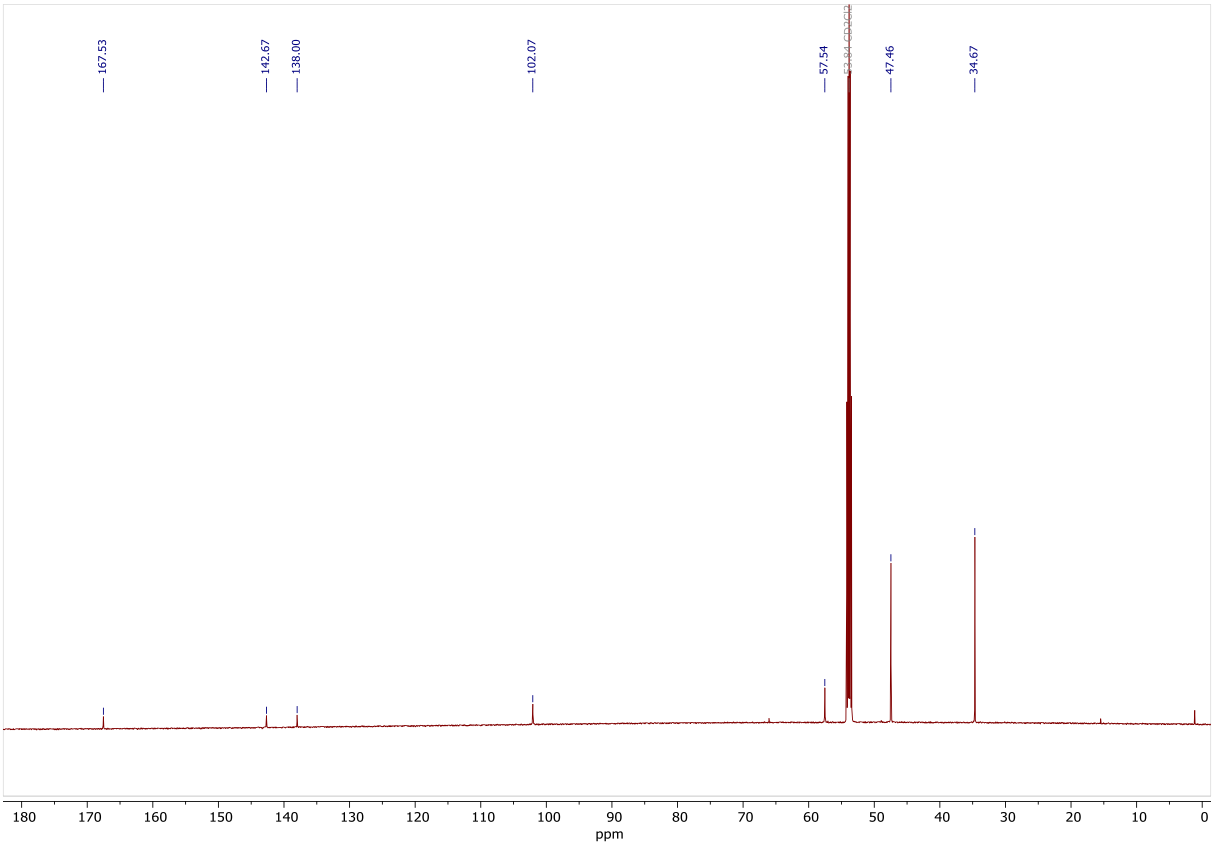

Figure S13: $^{13}C\{^{1}H\}$ NMR spectrum (151 MHz, CD_2Cl_2) of $[PdCl_2(L1)]$.

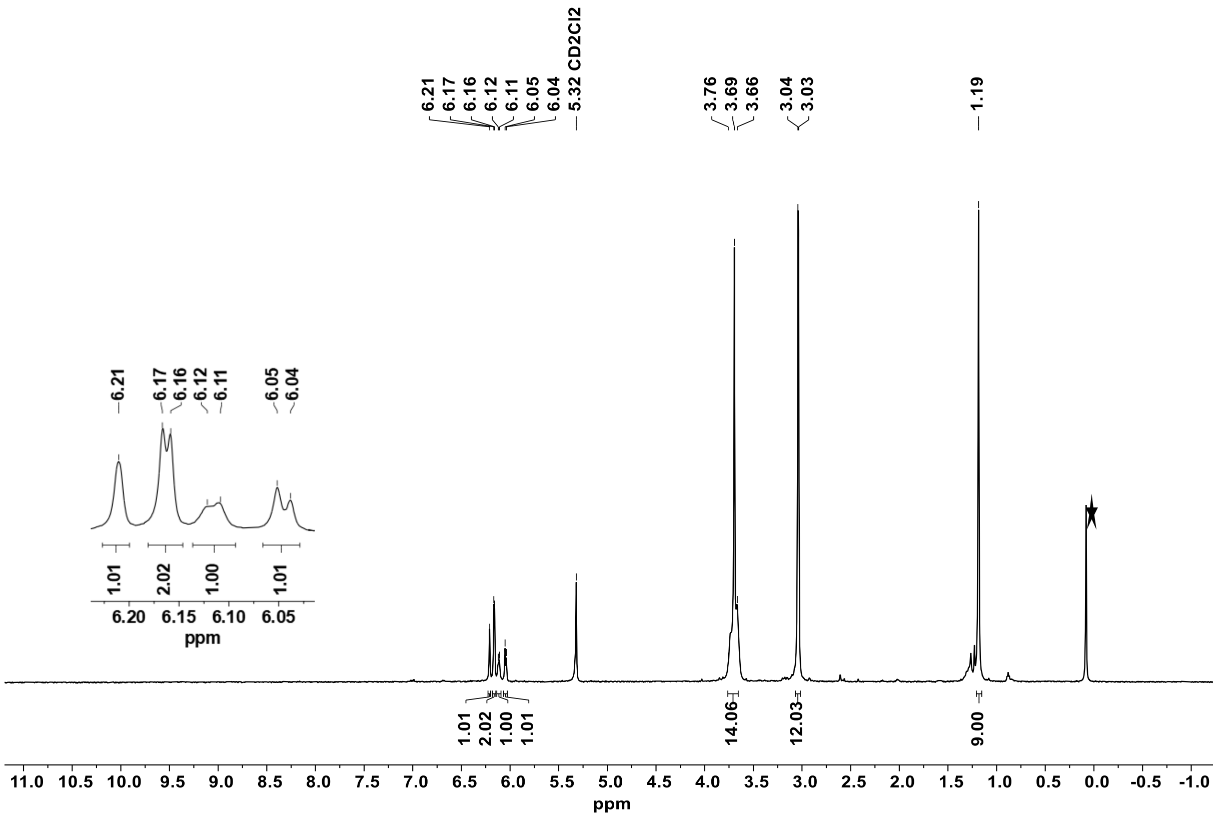

Figure S14: ^{1}H NMR spectrum (600 MHz, CD_2Cl_2) of [Pd(*t*Bu-cat)(L1)].

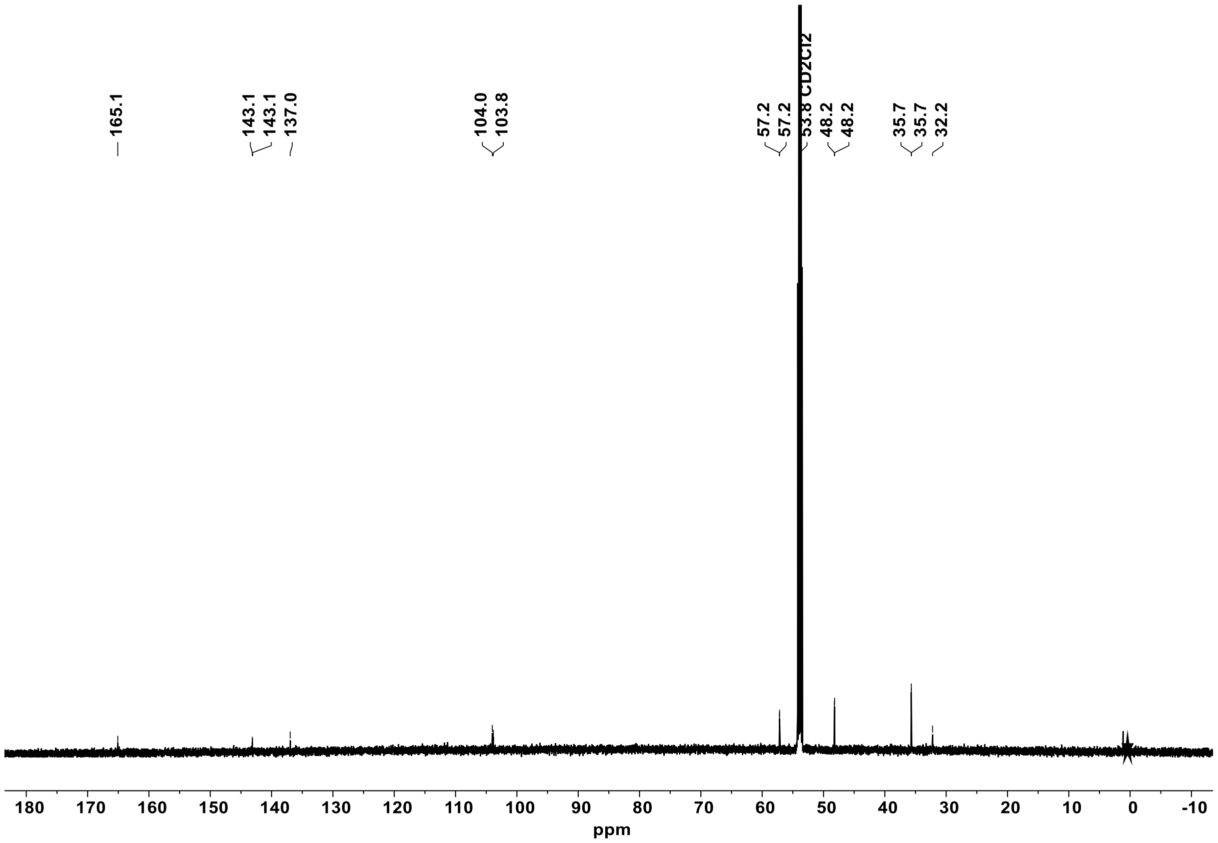

Figure S15: ^{13}C{^{1}H} NMR spectrum (151 MHz, CD_2Cl_2) of [Pd(tBu-cat)(L1)].

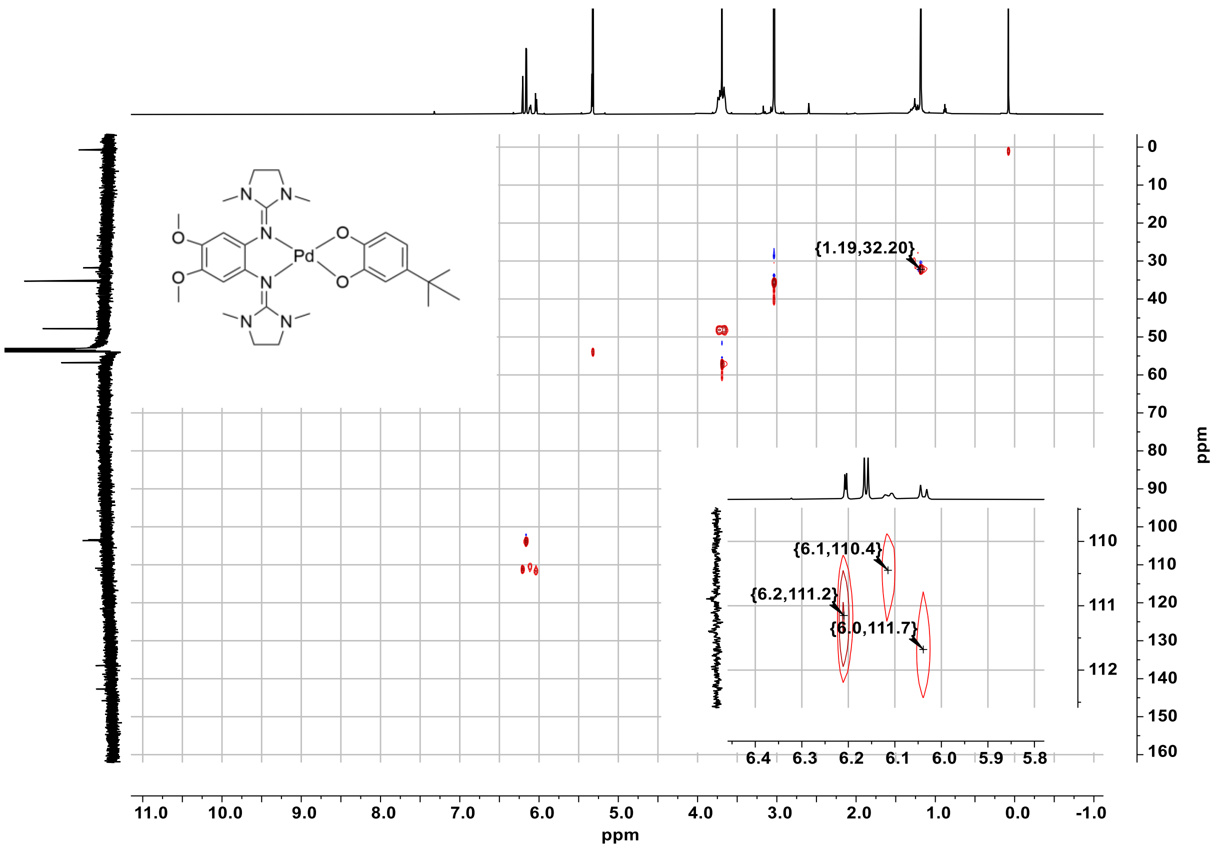

Figure S16: $^{1}H,^{13}C$ HSQC spectrum (CD_2Cl_2) of [Pd(tBu-cat)(L1)].

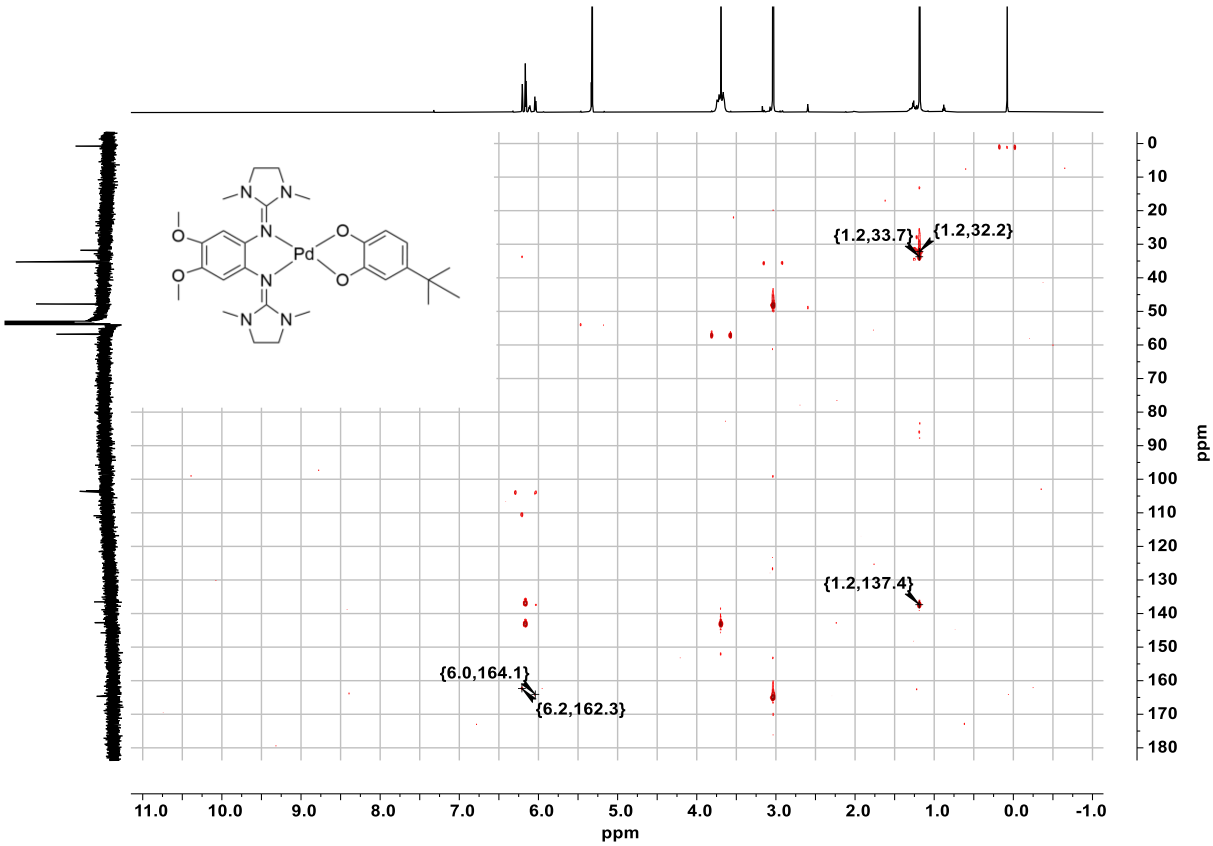

Figure S17: $^{1}H,^{13}C$ HMBC spectrum (CD_2Cl_2) of [Pd(tBu-cat)(L1)].

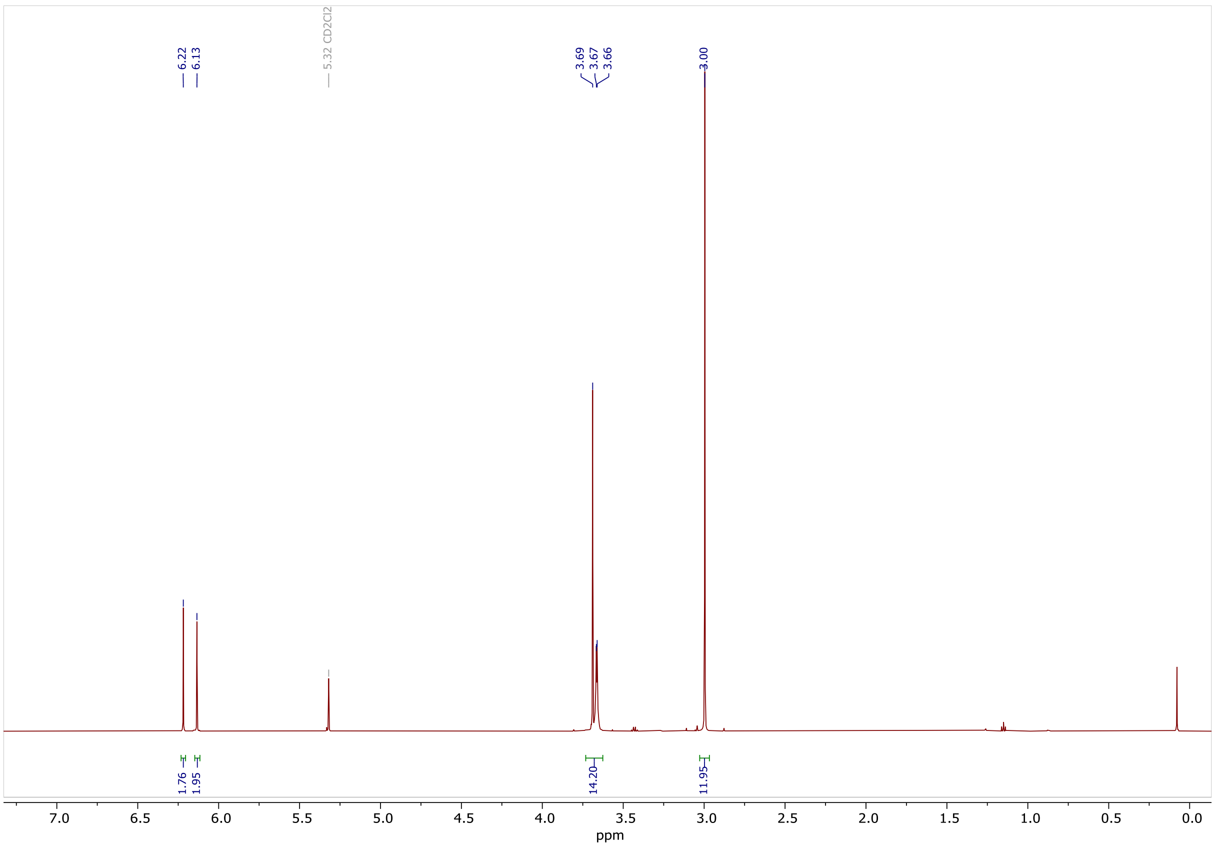

Figure S18: ^{1}H NMR spectrum (600 MHz, CD_2Cl_2) of [Pd(Cl_2-cat)(L1)].

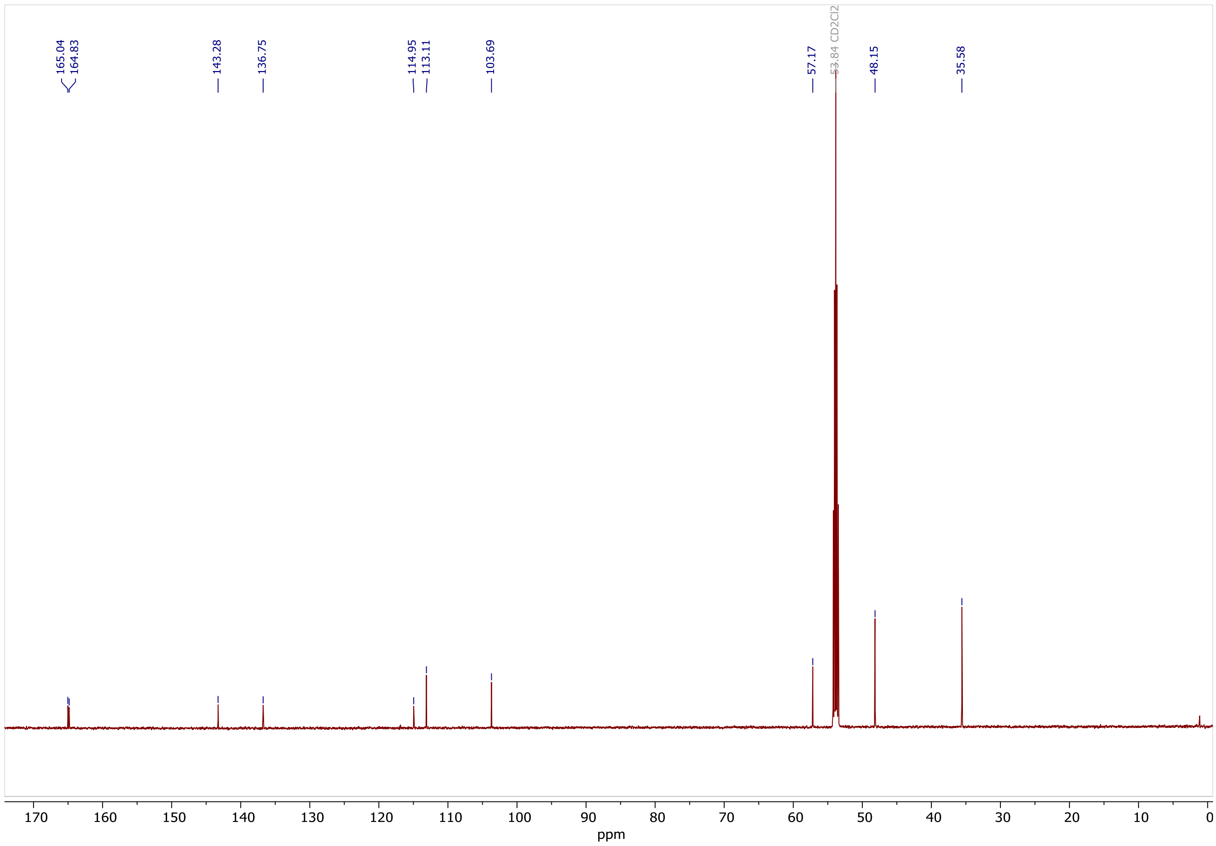

Figure S19: $^{13}C\{^{1}H\}$ NMR spectrum (151 MHz, CD_2Cl_2) of [Pd(Cl_2-cat)(L1)].

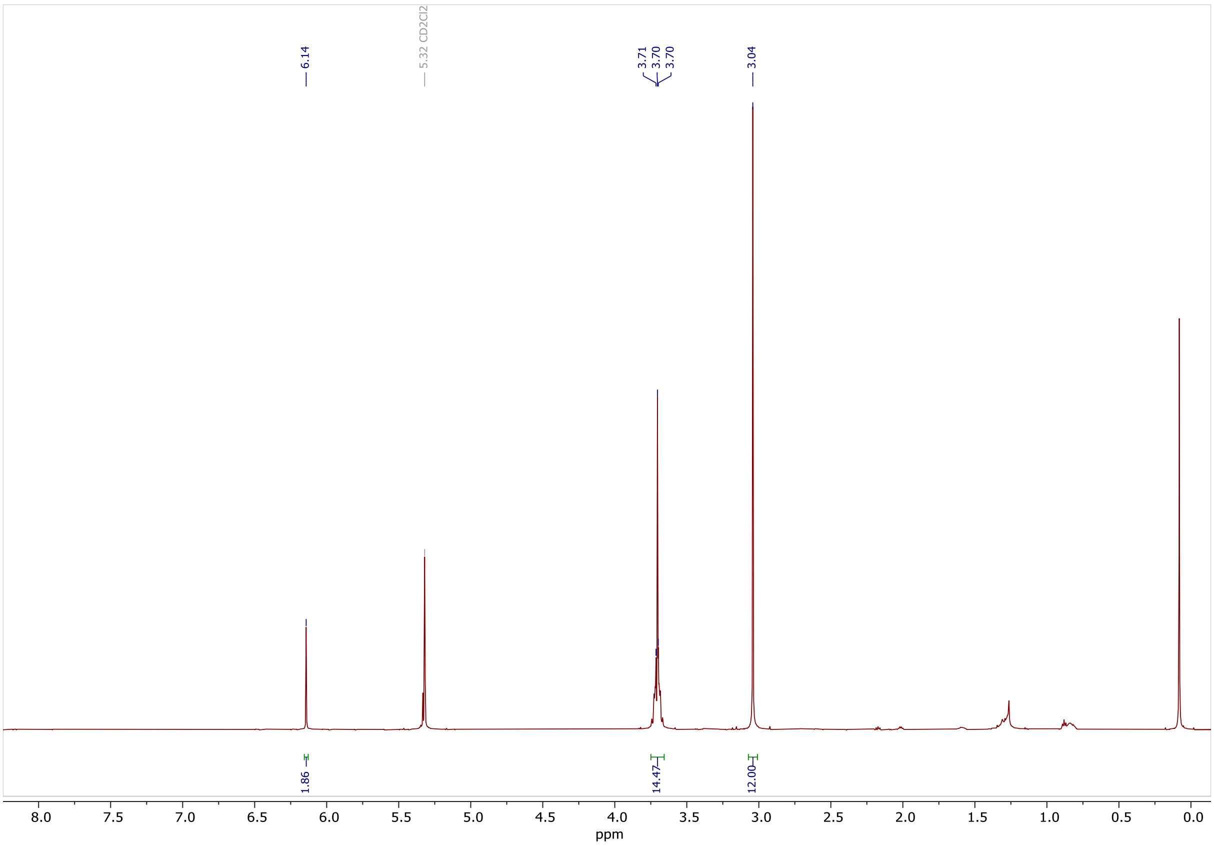

Figure S20: ^{1}H NMR spectrum (600 MHz, CD_2Cl_2) of [Pd(Cl_4-cat)(L1)].

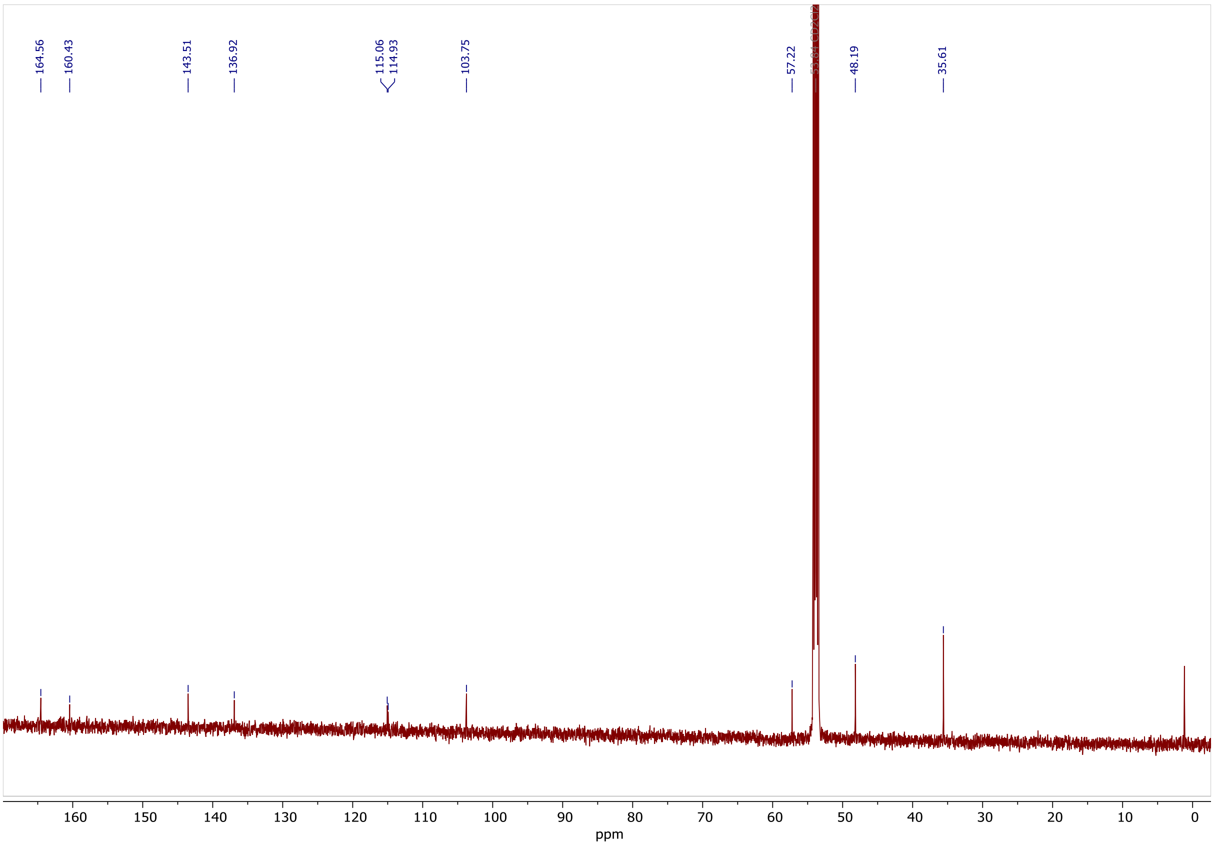

Figure S21: $^{13}C\{^{1}H\}$ NMR spectrum (151 MHz, CD_2Cl_2) of [Pd(Cl_4-cat)(L1)].

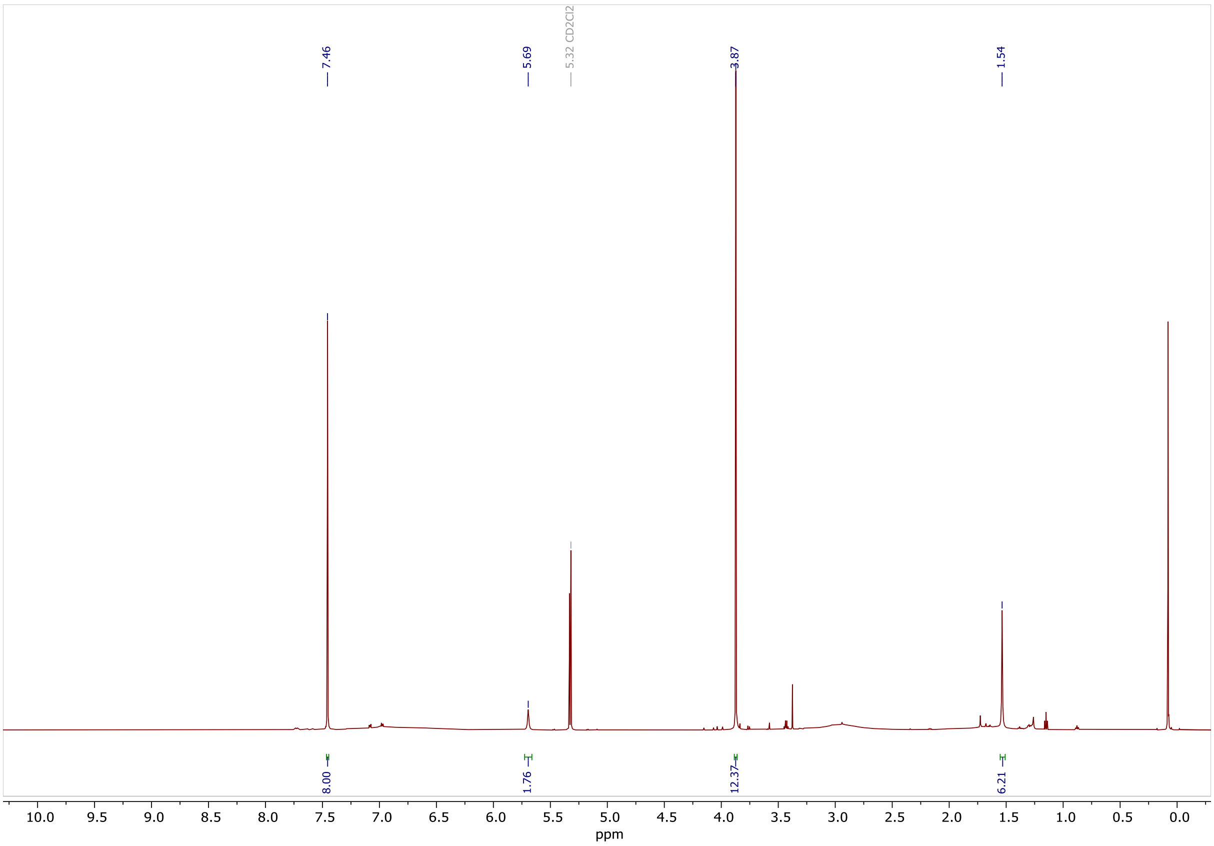

Figure S22: ^{1}H NMR spectrum (600 MHz, CD_2Cl_2) of [$PdCl_2$(L2)].

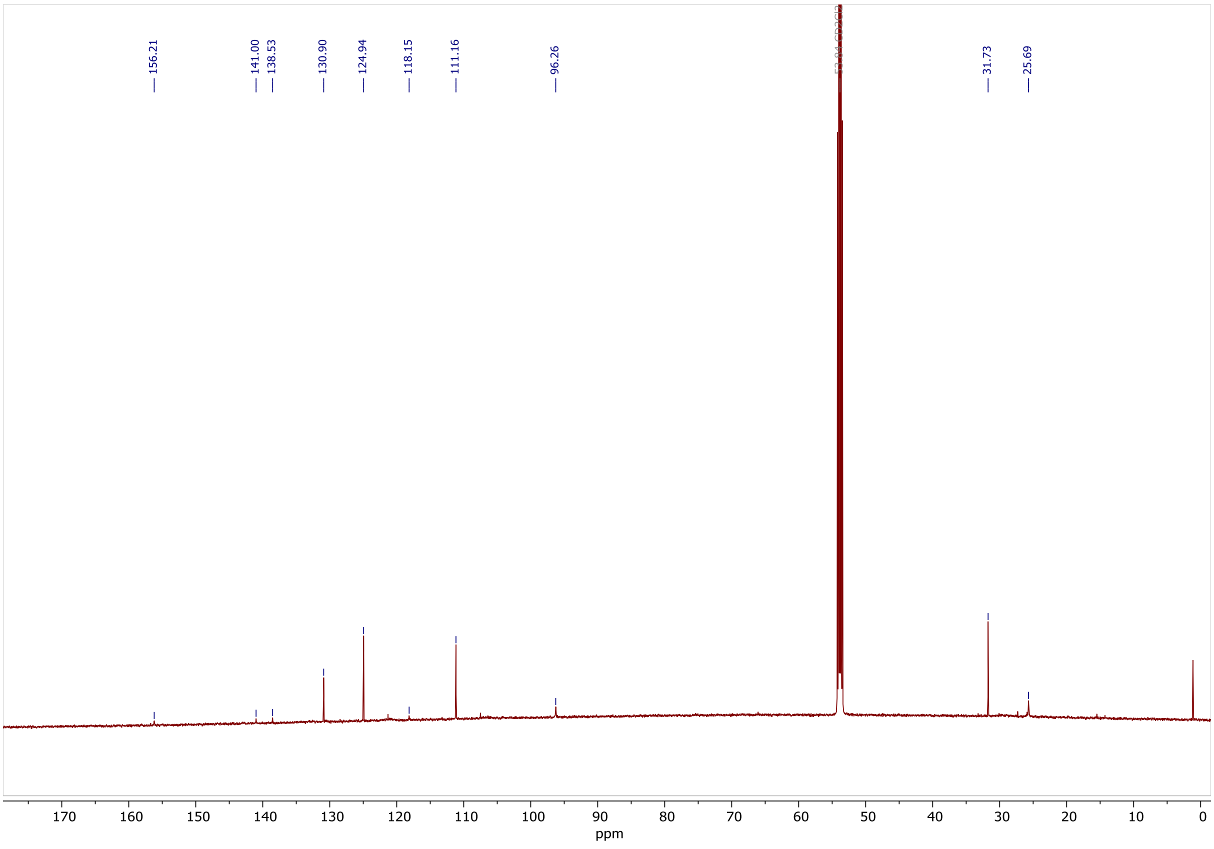

Figure S23: $^{13}C\{^{1}H\}$ NMR spectrum (151 MHz, CD_2Cl_2) of [$PdCl_2$(L2)].

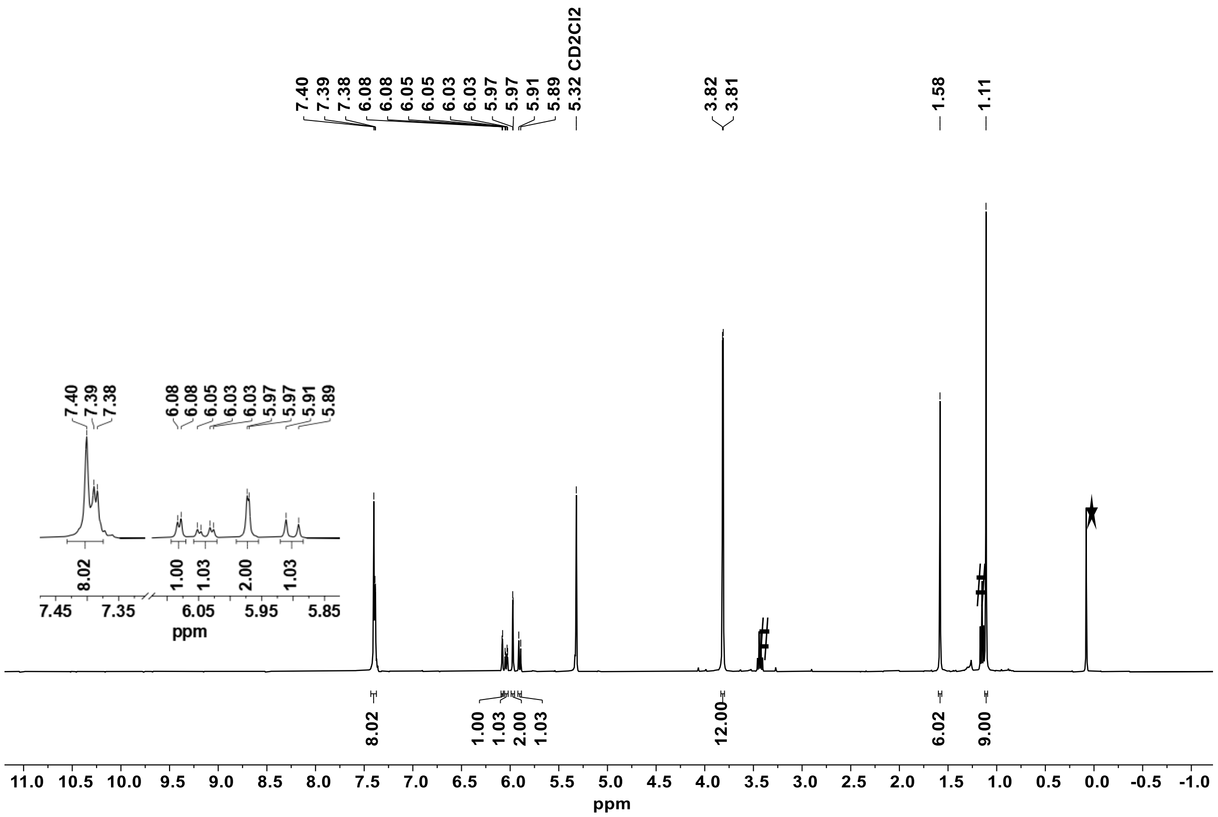

Figure S24: ^{1}H NMR spectrum (400 MHz, CD_2Cl_2) of [Pd(*t*Bu-cat)(L2)].

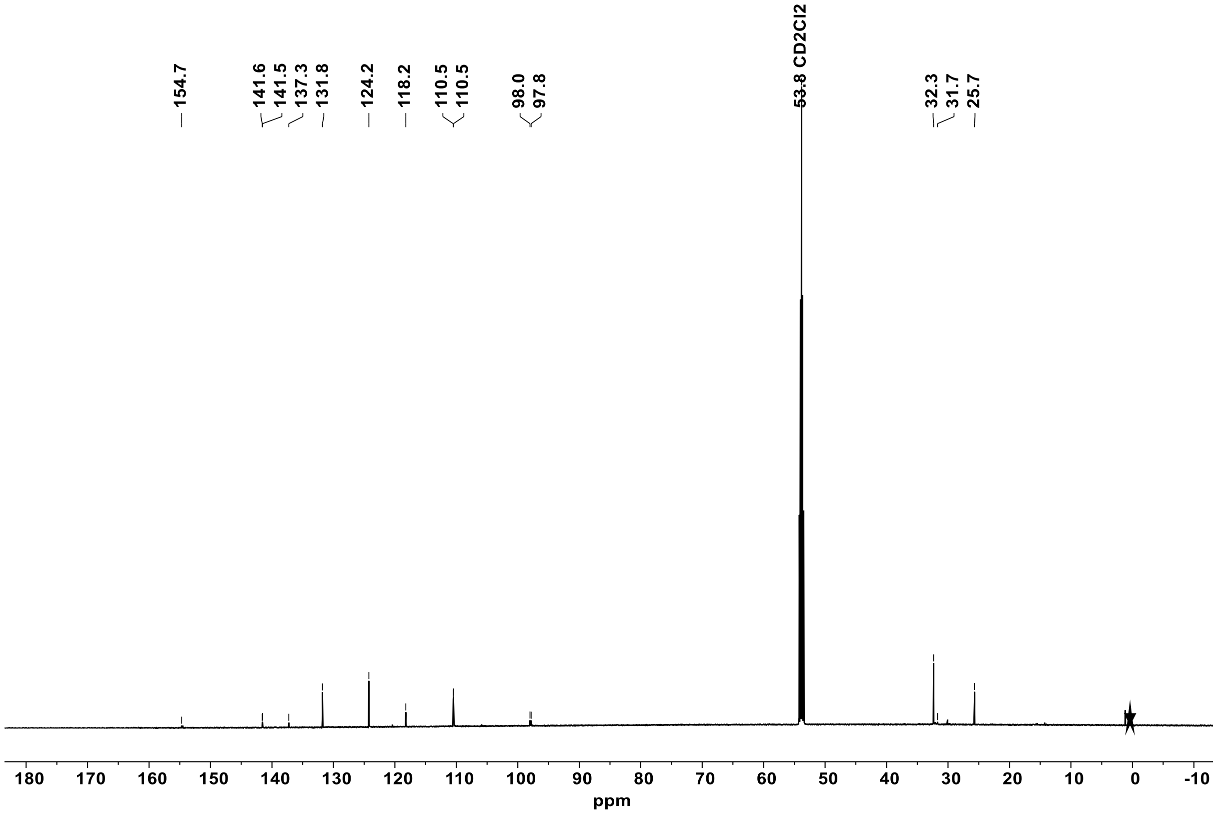

Figure S25: ^{13}C{^{1}H} NMR spectrum (151 MHz, CD_2Cl_2) of [Pd(tBu-cat)(L2)].

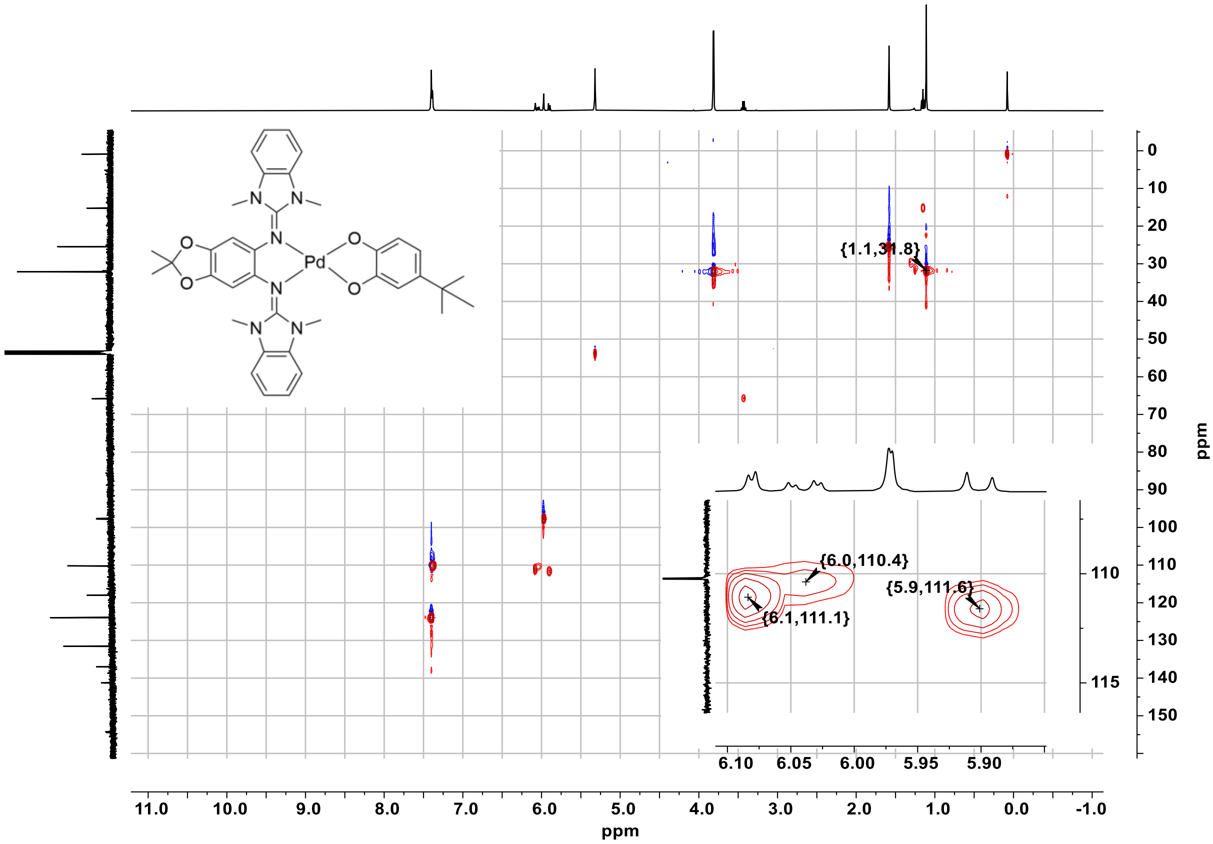

Figure S26: $^{1}H,^{13}C$ HSQC spectrum (CD_2Cl_2) of [Pd(tBu-cat)(L2)].

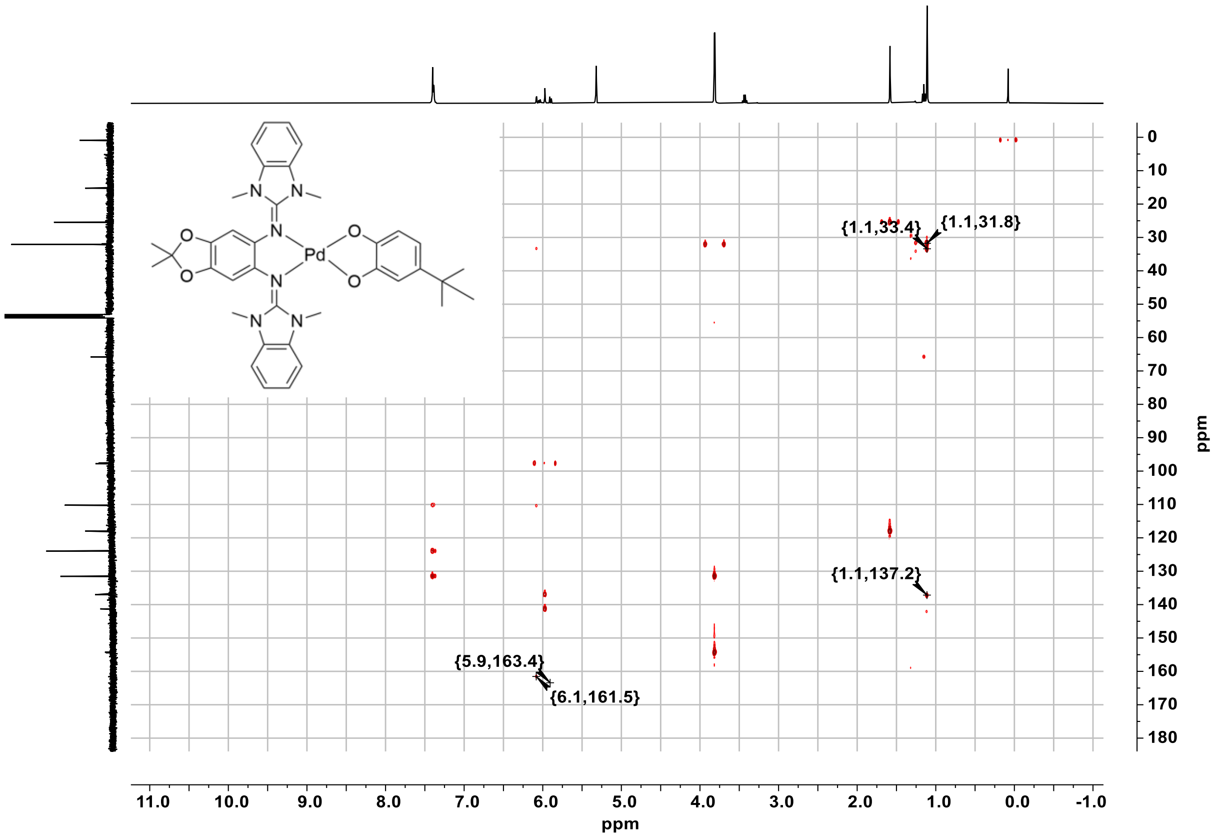

Figure S27: $^{1}H,^{13}C$ HMBC spectrum (CD_2Cl_2) of [Pd(tBu-cat)(L2)].

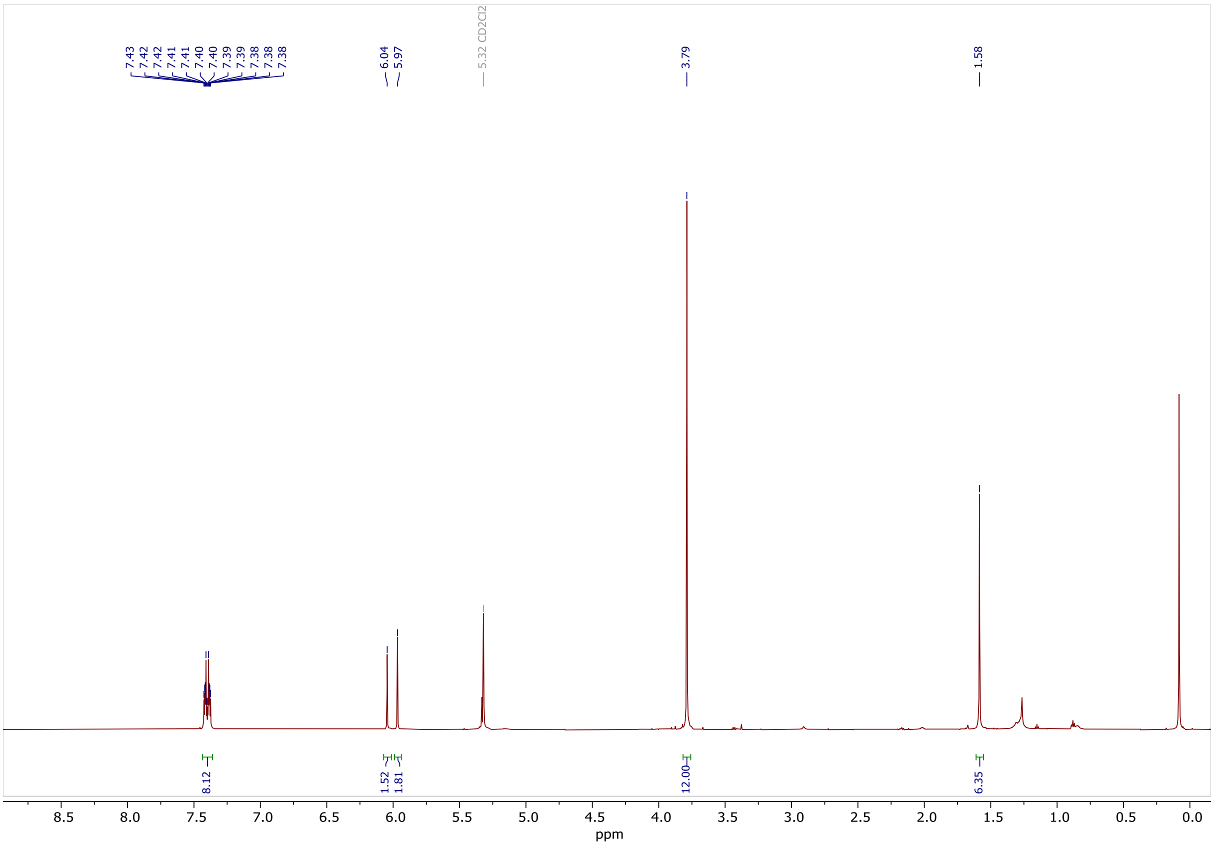

Figure S28: ^{1}H NMR spectrum (600 MHz, CD_2Cl_2) of [Pd(Cl_2-cat)(L2)].

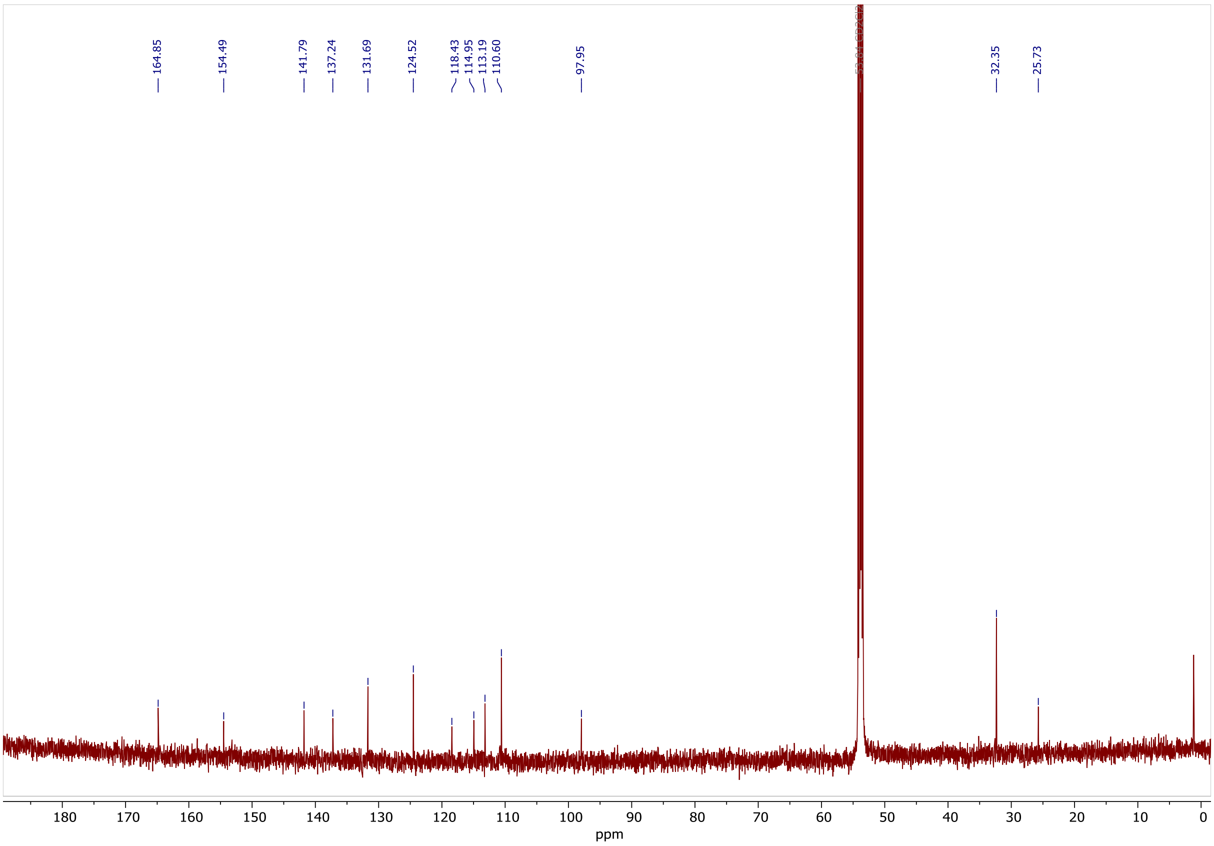

Figure S29: $^{3}C\{^{1}H\}$ NMR spectrum (151 MHz, CD_2Cl_2) of [Pd(Cl_2-cat)(L2)].

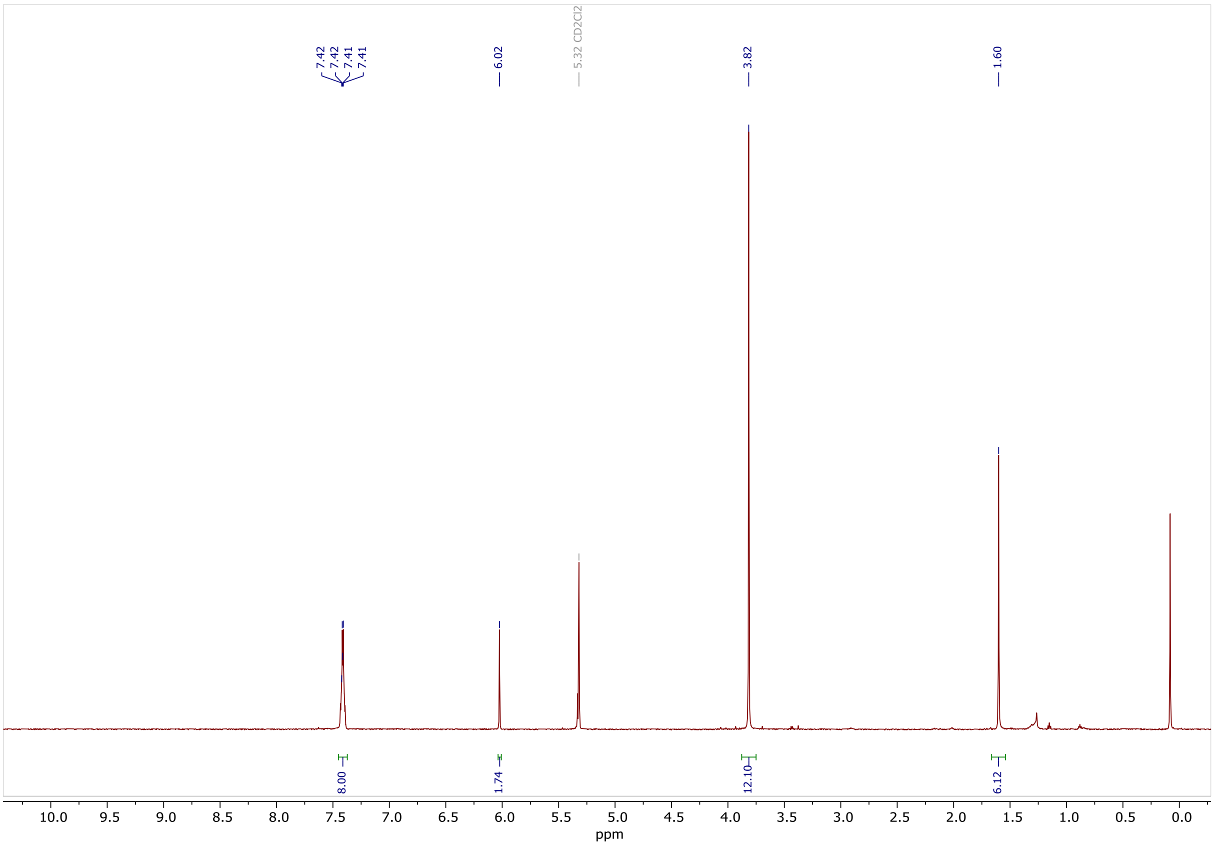

Figure S30: ^{1}H NMR spectrum (600 MHz, CD_2Cl_2) of [Pd(Cl_4-cat)(L2)].

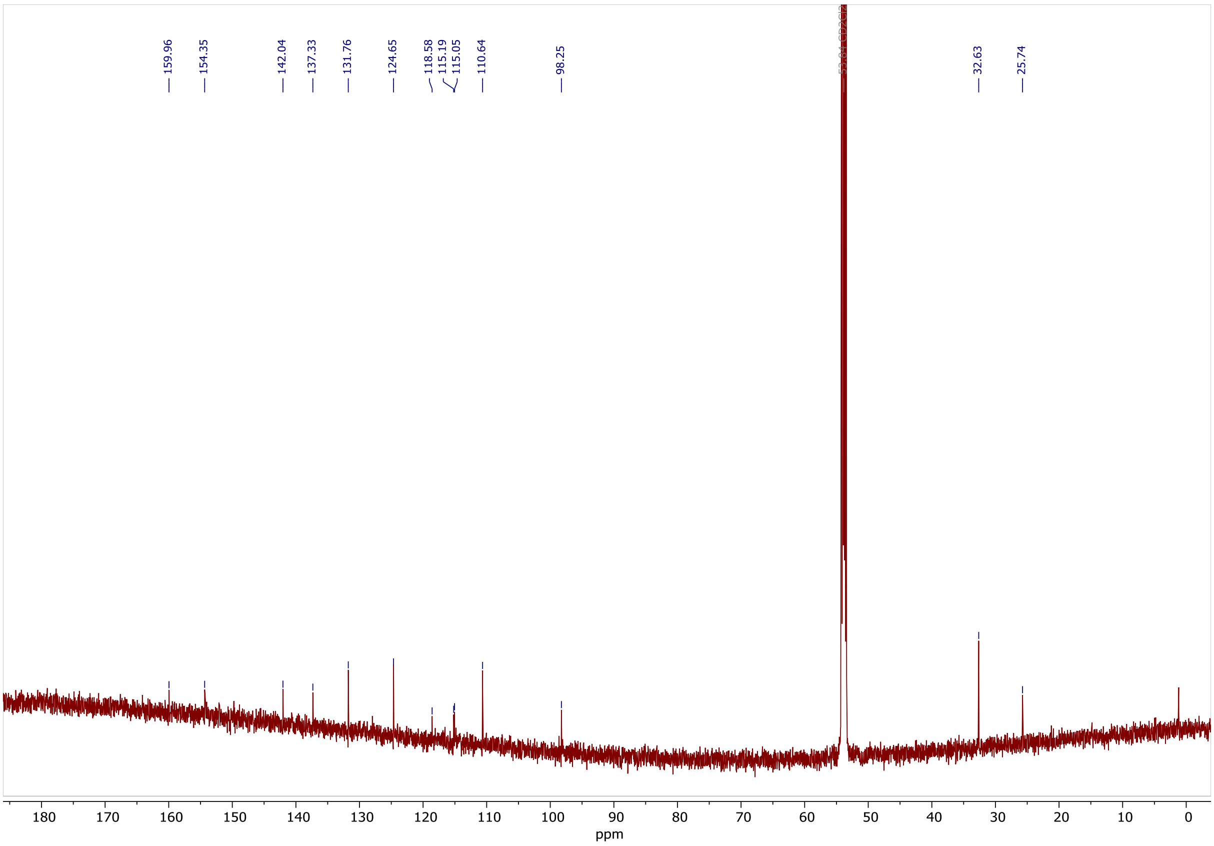

Figure S31: $^{13}C\{^{1}H\}$ NMR spectrum (151 MHz, CD_2Cl_2) of [Pd(Cl_4-cat)(L2)].

S4 Cyclic voltammetry

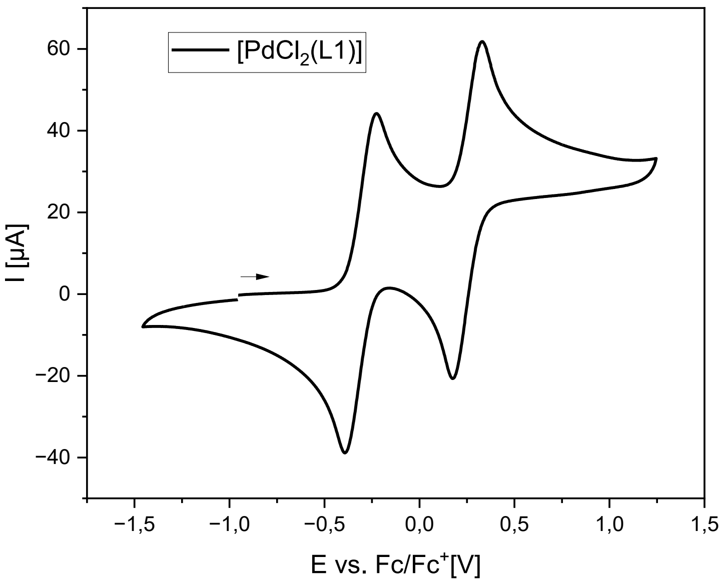

Figure S32: Cyclic voltammetry curve (CH_2Cl_2, Ag/AgCl, nBu_4NPF_6, scan rate 0.1 V/s, vs. Fc/Fc^+) of $[PdCl_2(L1)]$.

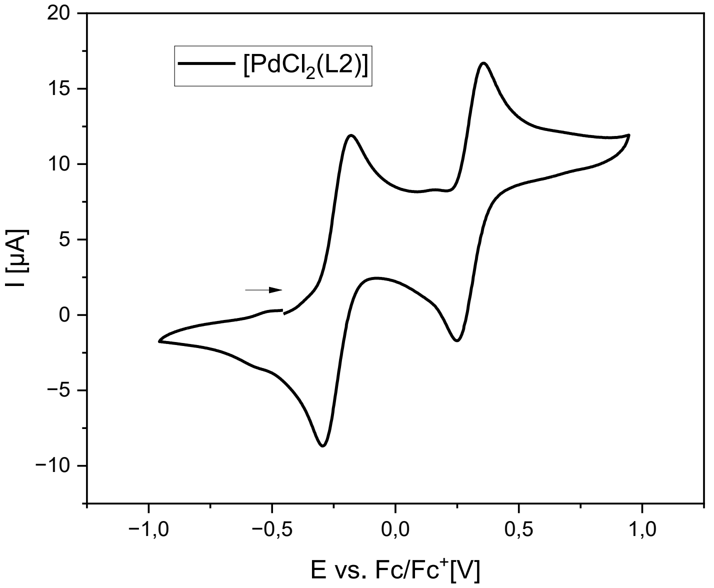

Figure S33: Cyclic voltammetry curve (CH_2Cl_2, Ag/AgCl, nBu_4NPF_6, scan rate 0.1 V/s, vs. Fc/Fc^+) of $[PdCl_2(L2)]$.

Table S1: Redox potentials vs. Fc/Fc^+($E_{1/2}$ values, with E_{ox} values in parentheses, both in V) for the six new palladium complexes (containing a GFA and a catecholate ligand) from CV measurements in dichloromethane.

Compound	1st redox	2nd redox	3rd redox
[Pd(*t*Bu-cat)(L1)]	-0.51 (-0.45)	-0.11 (-0.05)	0.46 (0.52)
[Pd(Cl_2-cat)(L1)]	-0.31 (-0.25)	0.03 (0.09)	0.53 (0.58)
[Pd(Cl_4-cat)(L1)]	-0.26 (-0.16)	0.24 (0.28)	0.70 (0.73)
[Pd(*t*Bu-cat)(L2)]	-0.47 (-0.38)	-0.04 (0.04)	0.55 (0.61)
[Pd(Cl_2-cat)(L2)]	-0.25 (-0.18)	0.06 (0.14)	0.61 (0.69)
[Pd(Cl_4-cat)(L2)]	-0.21 (-0.15)	0.20 (0.25)	0.68 (0.73)

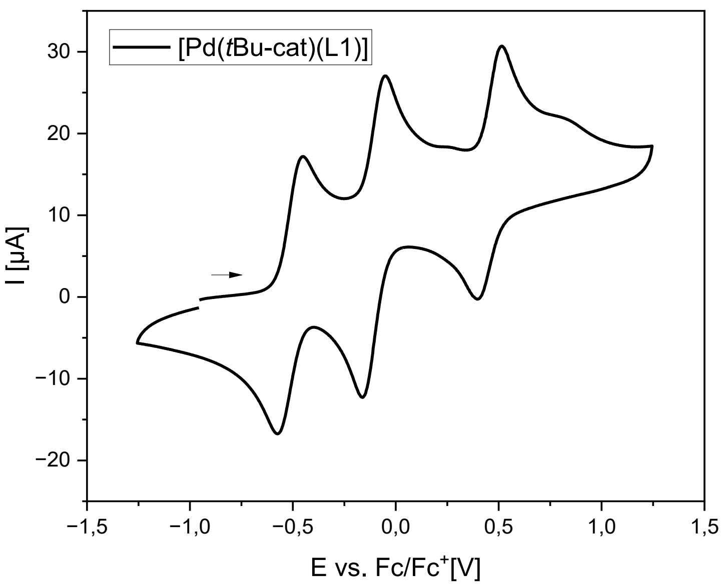

Figure S34: Cyclic voltammetry curve (CH_2Cl_2, Ag/AgCl, nBu_4NPF_6, scan rate 0.1 V/s, vs. Fc/Fc^+) of [Pd(tBu-cat)(L1)].

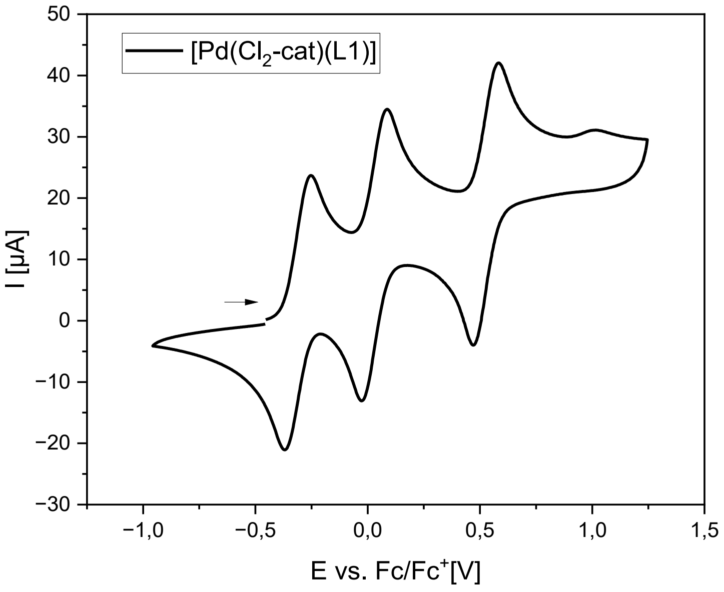

Figure S35: Cyclic voltammetry curve (CH_2Cl_2, Ag/AgCl, nBu_4NPF_6, scan rate 0.1 V/s, vs. Fc/Fc^+) of [Pd(Cl_2-cat)(L1)].

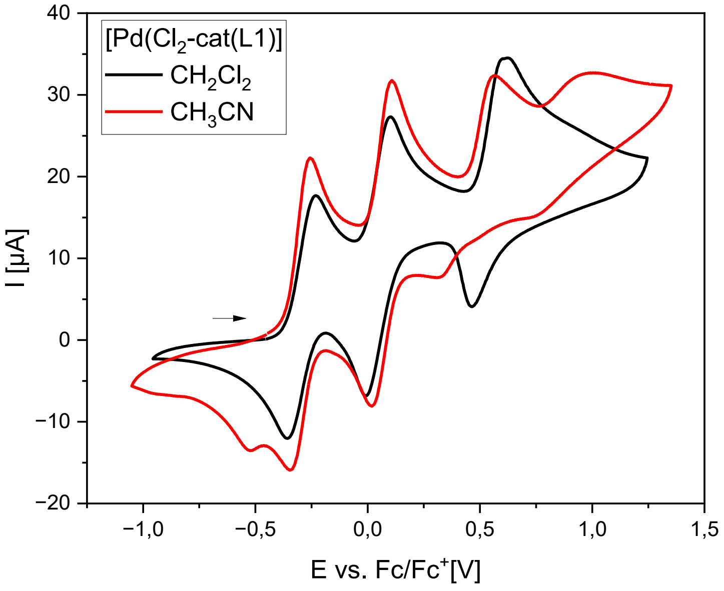

Figure S36: Comparison of the cyclic voltammetry curves (Ag/AgCl, nBu_4NPF_6, scan rate 0.1 V/s, vs. Fc/Fc^+) of [Pd(Cl_2-cat)(L1)] in DCM and ACN. The decrease in reversibility observed in CH_3CN is most likely due to decomposition of the highly oxidised species in presence of a coordinating solvent.

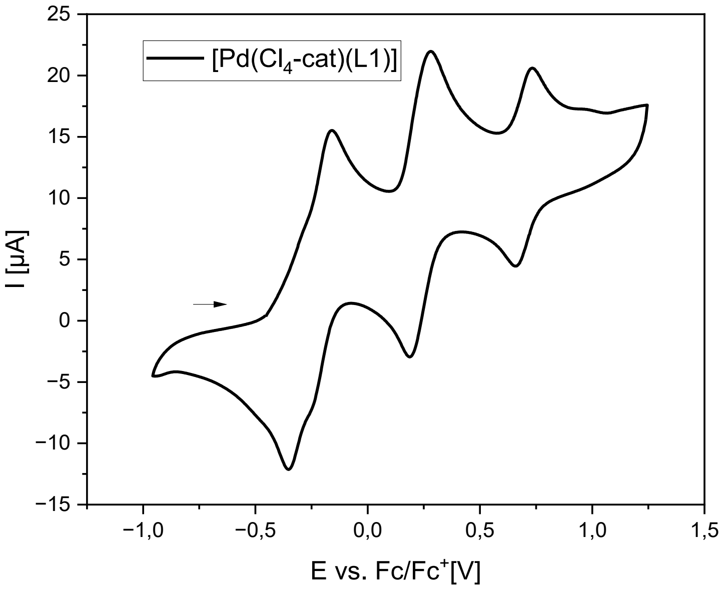

Figure S37: Cyclic voltammetry curve (CH_2Cl_2, Ag/AgCl, nBu_4NPF_6, scan rate 0.1 V/s, vs. Fc/Fc^+) of [Pd(Cl_4-cat)(L1)].

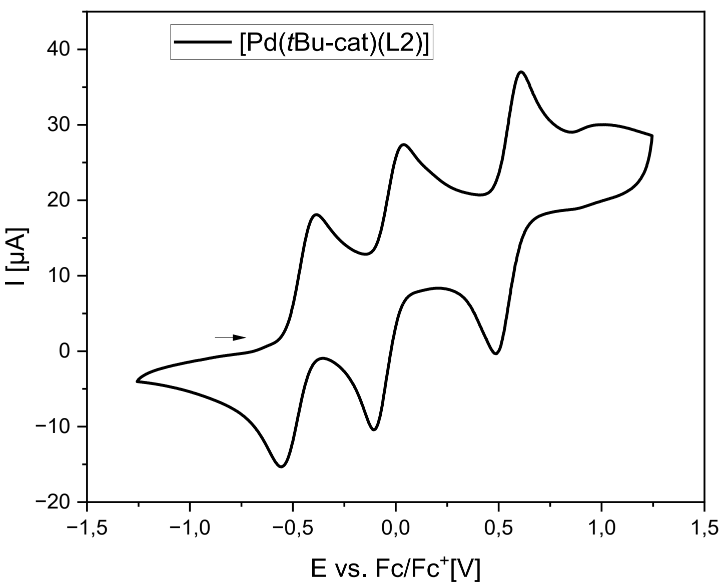

Figure S38: Cyclic voltammetry curve (CH_2Cl_2, Ag/AgCl, nBu_4NPF_6, scan rate 0.1 V/s, vs. Fc/Fc^+) of [Pd(tBu-cat)(L2)].

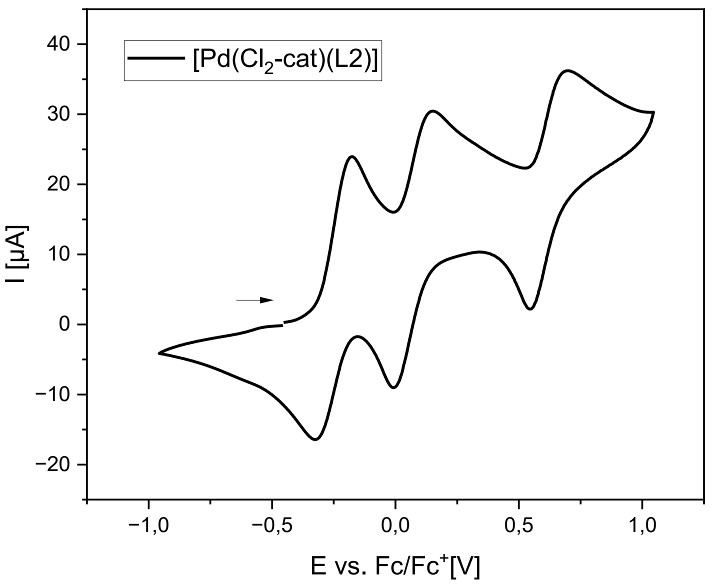

Figure S39: Figure 1: Cyclic voltammetry curve (CH_2Cl_2, Ag/AgCl, nBu_4NPF_6, scan rate 0.1 V/s, vs. Fc/Fc^+) of [Pd(Cl_2-cat)(L2)].

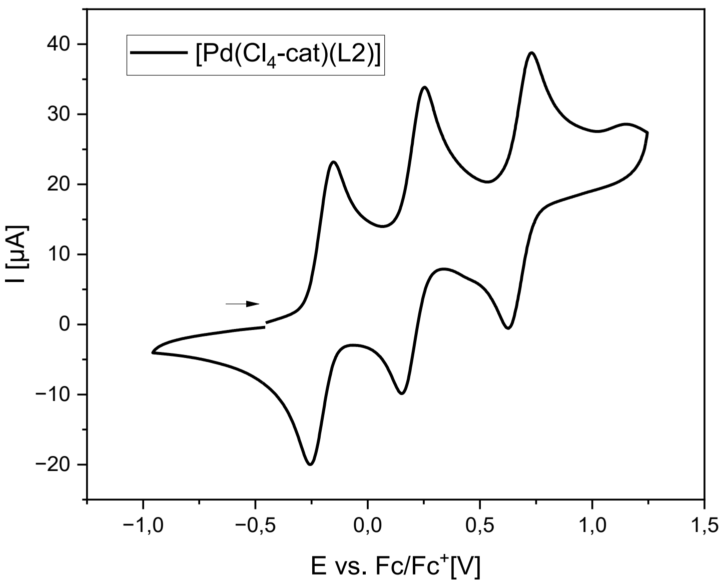

Figure S40: Cyclic voltammetry curve (CH_2Cl_2, Ag/AgCl, nBu_4NPF_6, scan rate 0.1 V/s, vs. Fc/Fc^+) of [Pd(Cl_4-cat)(L2)].

S5 X-Band EPR spectroscopy

S5.1 Spectra in CH_2Cl_2 solution at room temperature

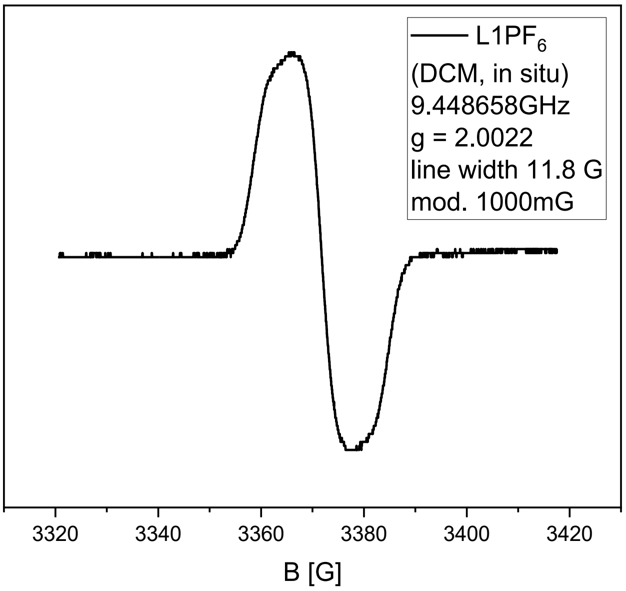

Figure S41: X-band EPR spectrum (CH_2Cl_2) of *in situ* generated L1PF_6 (0.5 eq. Ferrocenium hexafluorophosphate).

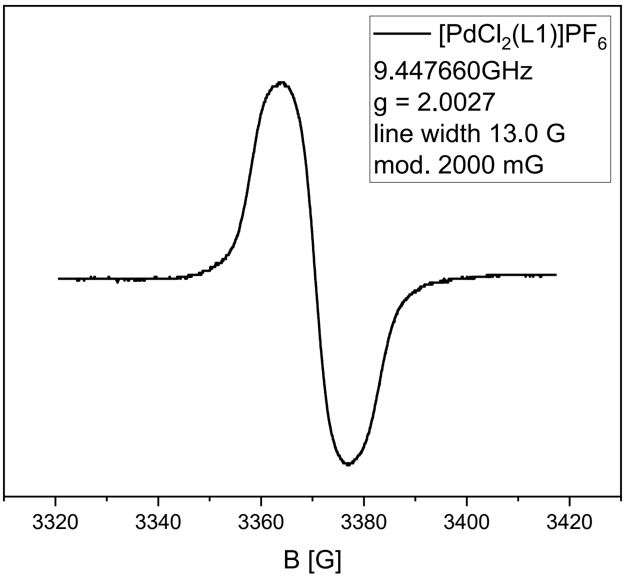

Figure S42: X-band EPR spectrum (CH_2Cl_2) of $[PdCl_2(L1)]PF_6$.

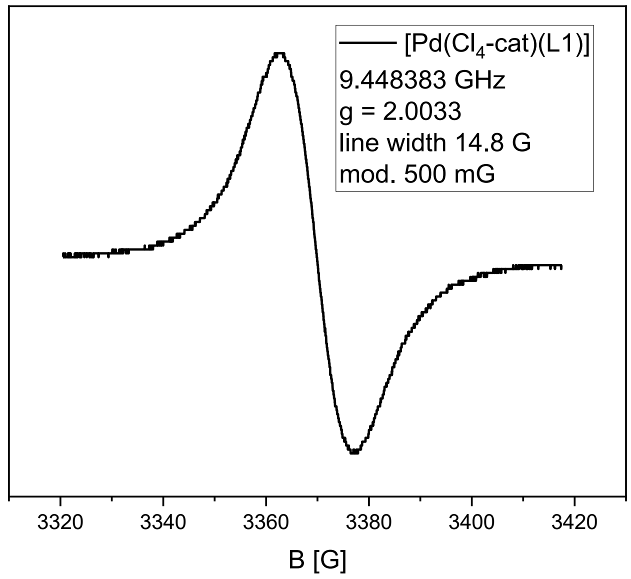

Figure S43: X-band EPR spectrum (CH_2Cl_2) of the *in situ* oxidation of [Pd(Cl_4-cat)(L1)] with 0.5 eq. ferrocenium hexfluorophosphate.

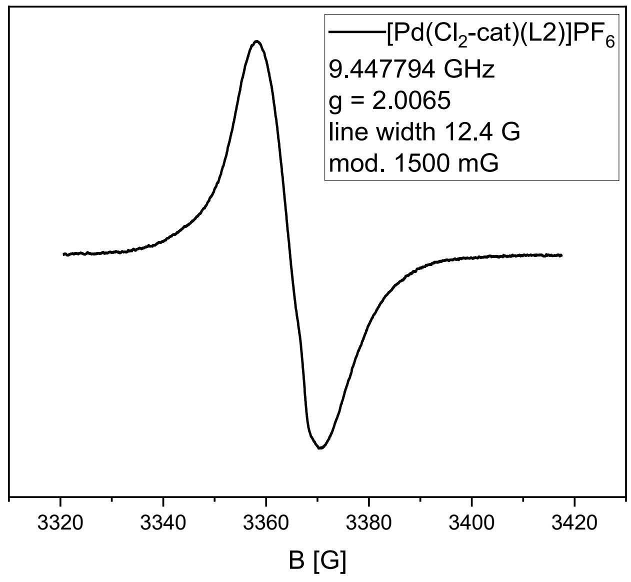

Figure S44: X-band EPR spectrum (CH_2Cl_2) of [Pd(Cl_2-cat)(L2)]PF_6. The g value and line width is given for the broad signal (not considering the slight shoulder on the inside of the signal).

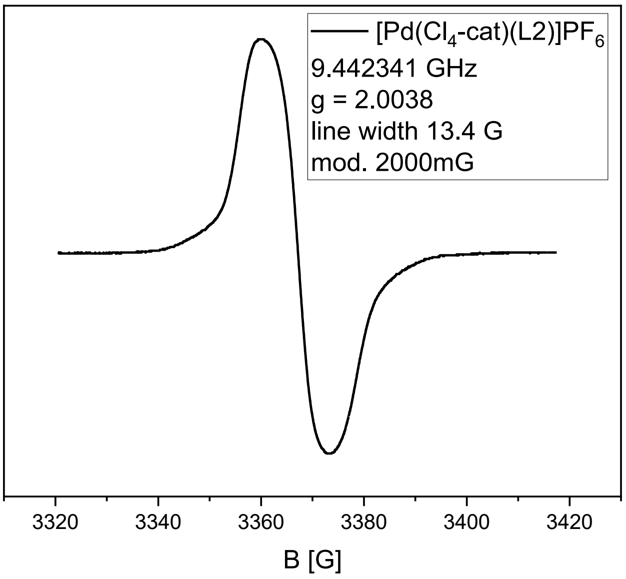

Figure S45: X-band EPR spectrum (CH_2Cl_2) of $[Pd(Cl_4\text{-cat})(L2)]PF_6$.

S5.2 Simulations with EasySpin

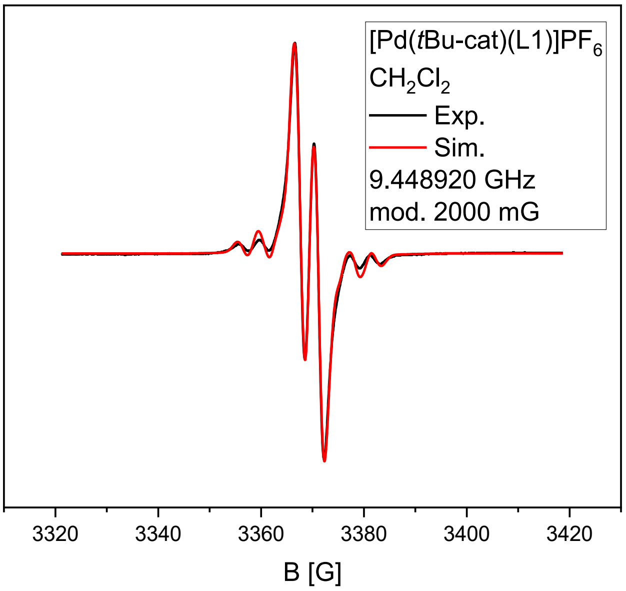

Figure S46: X-band EPR spectrum (CH_2Cl_2) of [Pd(tBu-cat)(L1)]PF_6. Simulation parameters (GFA): 34.6%, g=2.0039, a(Pd)=4.43G, a(2N)=3.52G, a(4N)=1.45G; lwpp 0.168. Simulation parameters (SQ): 65.4%, g=2.0042;A(Pd)=4.40G; a(1H)=3.50G; lwpp 0.241. (rsmd 226.27).

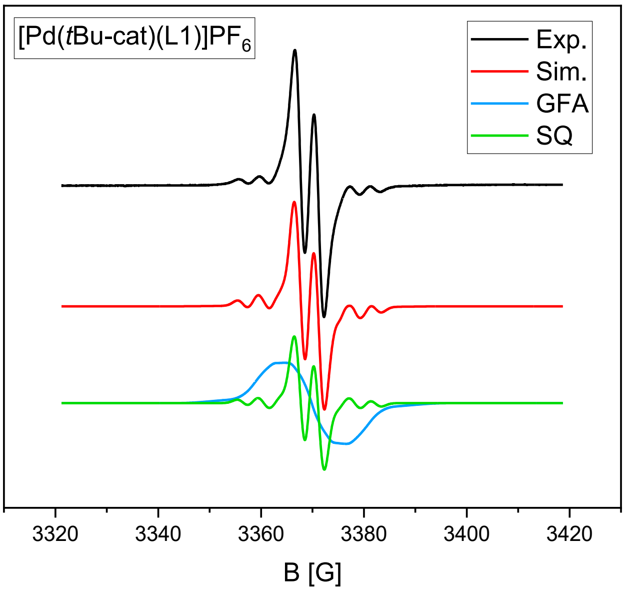

Figure S47: Contributions of the GFA and SQ components to the EPR simulation ([Pd(tBu-cat)(L1)]PF_6).

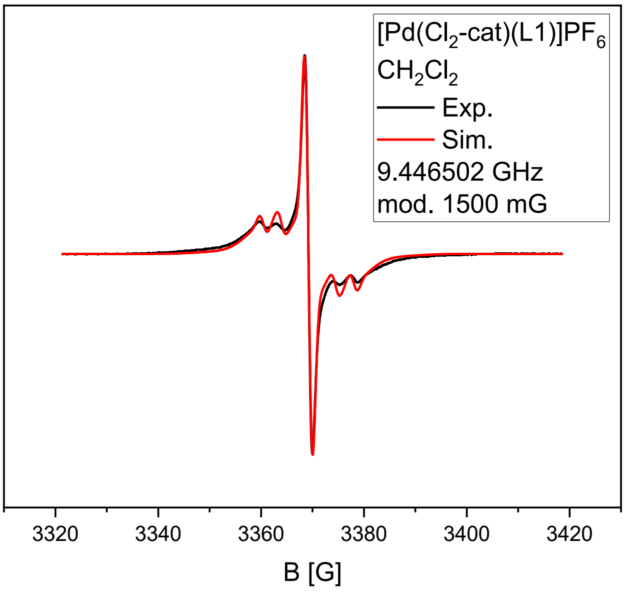

Figure S48: X-band EPR spectrum (CH_2Cl_2) of [Pd(Cl_2-cat)(L1)]PF_6. Simulation parameters (GFA): 89%, g=2.0039, a(Pd)=4.67, a(2N)=3.84, a(4N)=1.33, lwpp=0.468. Simulation parameters (SQ): 11%, g=2.00377, a(Pd)=3.50 G, lwpp=0.159. (rsmd 270.454).

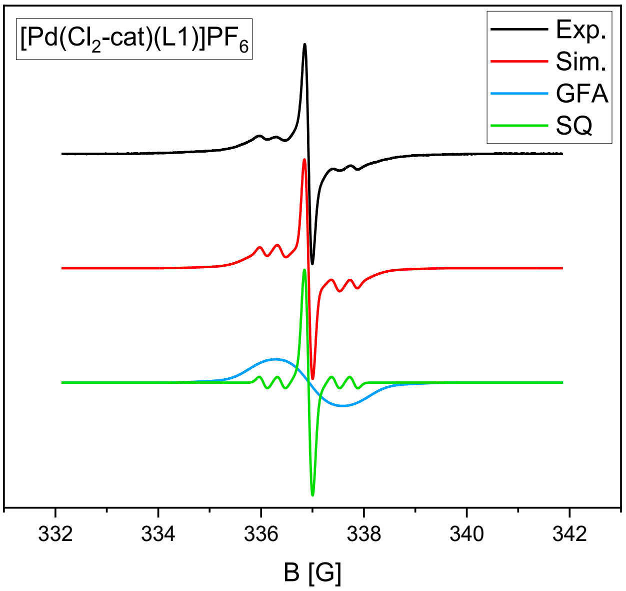

Figure S49: Contributions of the GFA and SQ components to the EPR simulation ([Pd(Cl_2-cat)(L1)]PF_6).

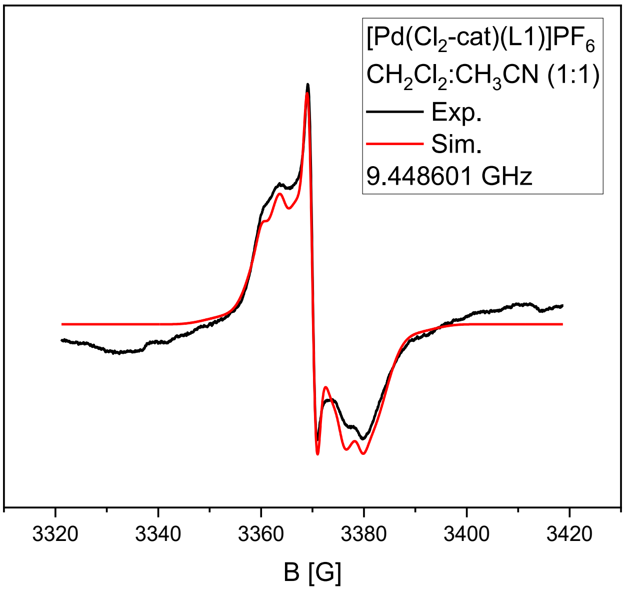

Figure S50: X-band EPR spectrum (CH_2Cl_2:CH_3CN (1:1)) of [Pd(Cl_2-cat)(L1)]PF_6. Simulation parameters (GFA): 97%, g(iso)=2.0029, A(Pd)= 4.34 G; A(2 N) = 4.17 G; A(4 N) = 1.50 G (taken to be close to calculated from other spectra but not limited during this simulation), lwpp = 0.3308. Simulation parameters (SQ): 3%, g(iso)=2.0038, A(Pd)= 3.66 G; lwpp = 0.1842.

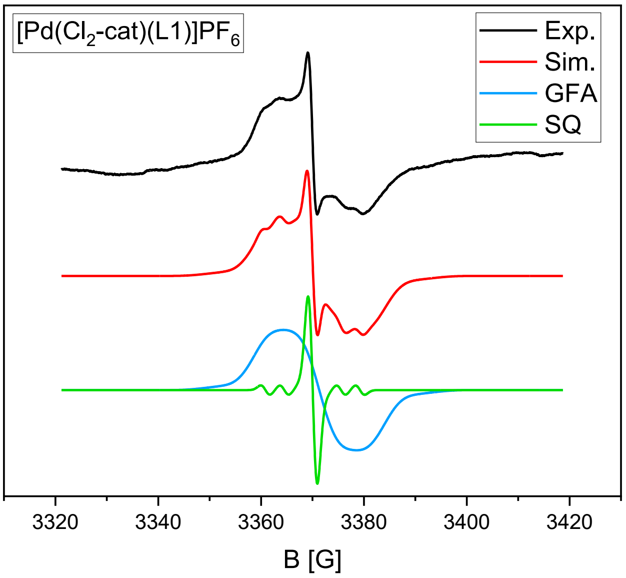

Figure S51: Contributions of the GFA and SQ components to the EPR simulation ([Pd(Cl_2-cat)(L1)]PF_6).

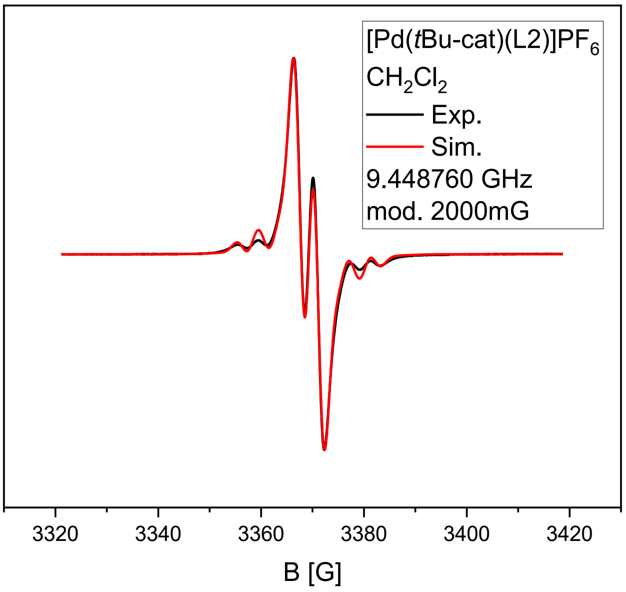

Figure S52: X-band EPR spectrum (CH_2Cl_2) of [Pd(tBu-cat)(L2)]PF_6. Simulation parameters (GFA): 43.2%, g = 2.0036, a(Pd)=4.49G, a(2N)=3.35G, a(4N)=1.458G; lwpp 0.278115. Simulation parameters (SQ): 56.8%, g=2.0037;A(Pd)=4.50G; a(1H)=3.51G; lwpp 0.264291. (rsmd 225.473).

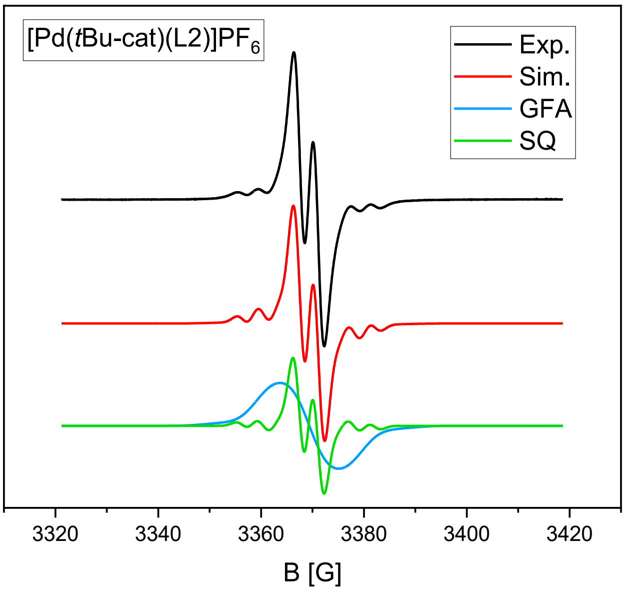

Figure S53: Contributions of the GFA and SQ components to the EPR simulation ([Pd(tBu-cat)(L2)]PF_6).

Table S2: The g and A values obtained from the fits of the X-band EPR spectra (CH_2Cl_2, room temperature).

	Parameter	[Pd(*t*Bu-cat)(L1)]$^+$	[Pd(Cl_2-cat)(L1)]$^+$	[Pd(*t*Bu-cat)(L2)]$^+$
L1 or L2	g	2.0039	2.0039	2.0036
	A (^{105}Pd)	4.4 G	4.7 G	4.5 G
	A (2N)	3.5 G	3.8 G	3.4 G
	A (4N)	1.5 G	1.3 G	1.5 G
SQ	g	2.0042	2.0038	2.0037
	A (^{105}Pd)	4.4 G	3.5 G	4.5 G
	A (^{1}H)	3.5 G	-	3.5 G
	Ratio of SQ [%]	65%	11%	57%

S5.3 Additional EPR spectra

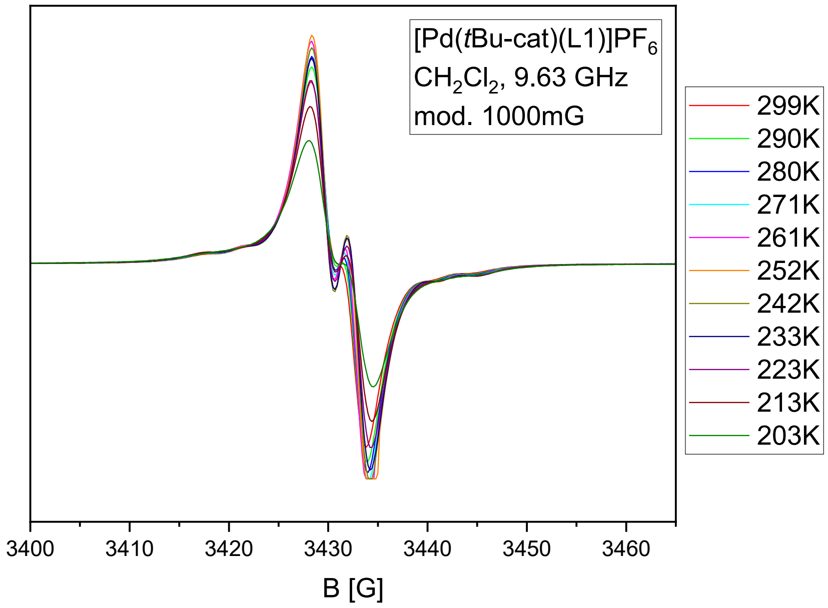

Figure S54: X-band EPR spectrum (CH_2Cl_2) of [Pd(tBu-cat)(L1)]PF_6 at different temperatures.

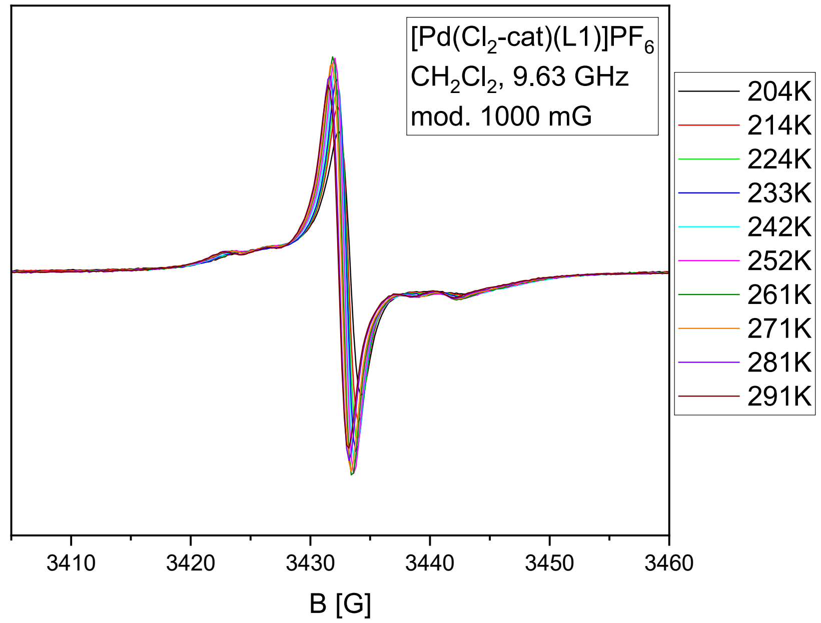

Figure S55: X-band EPR spectrum (CH_2Cl_2) of [Pd(Cl_2-cat)(L1)]PF_6 at different temperatures.

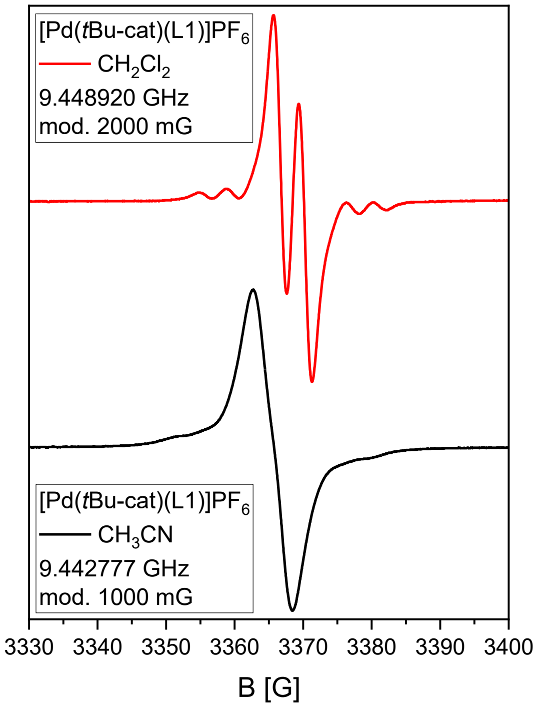

Figure S56: X-band EPR spectrum of [Pd(tBu-cat)(L1)]PF_6 in CH_2Cl_2 and CH_3CN.

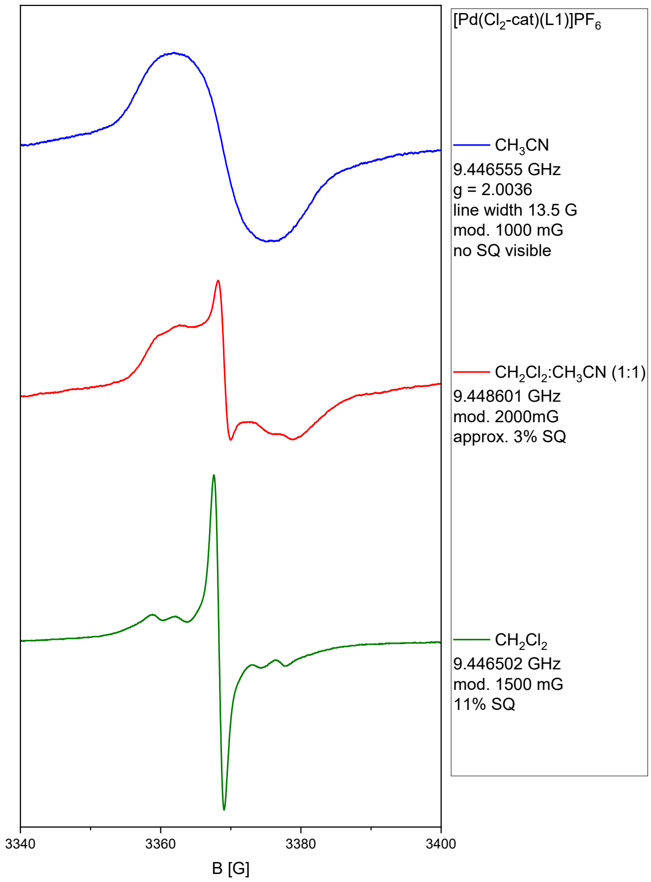

Figure S57: X-band EPR spectra of [Pd(Cl_2-cat)(L1)]PF_6 with varying solvent polarity.

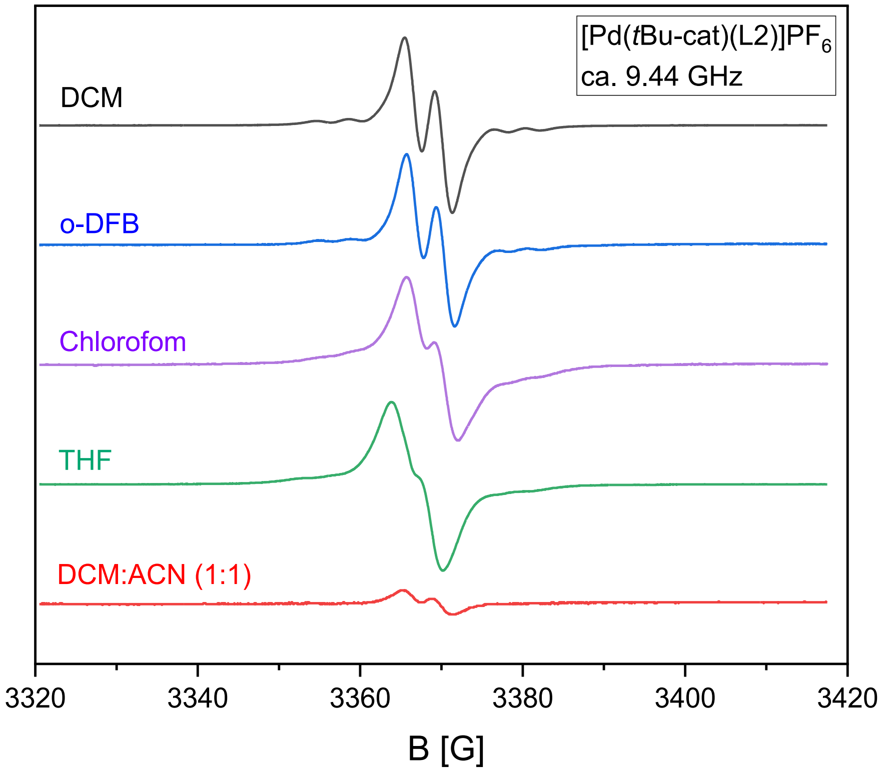

Figure S58: X-band EPR spectra of [Pd(*t*Bu-cat)(L2)]PF_6 in different solvents.

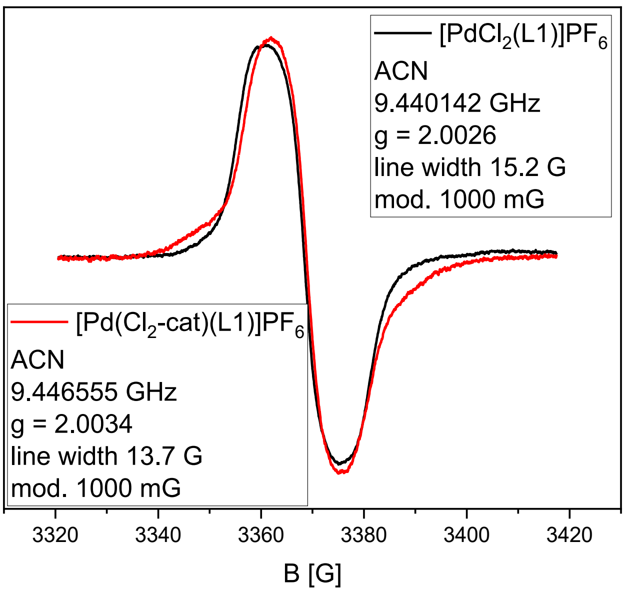

Figure S59: Comparison of the X-band EPR spectra (CH_3CN) of [$PdCl_2$(L1)]PF_6 and [Pd(Cl_2-cat)(L1)]PF_6.

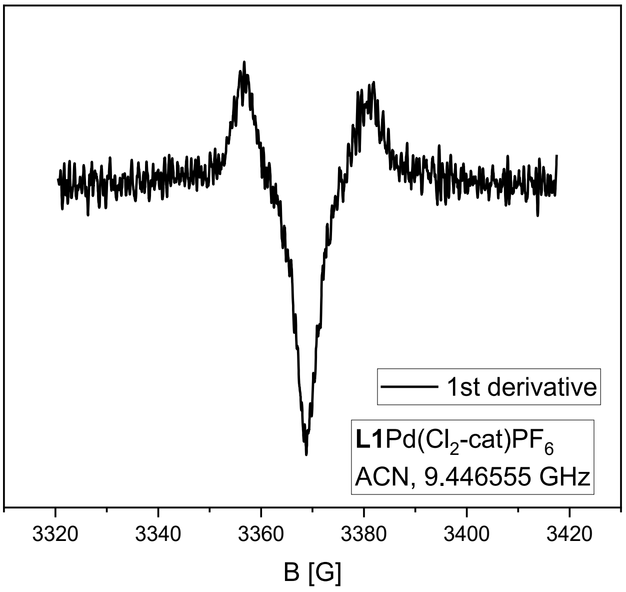

Figure S60: First derivate of the X-band EPR spectrum (CH_3CN) of [Pd(Cl_2-cat)(L1)]PF_6.

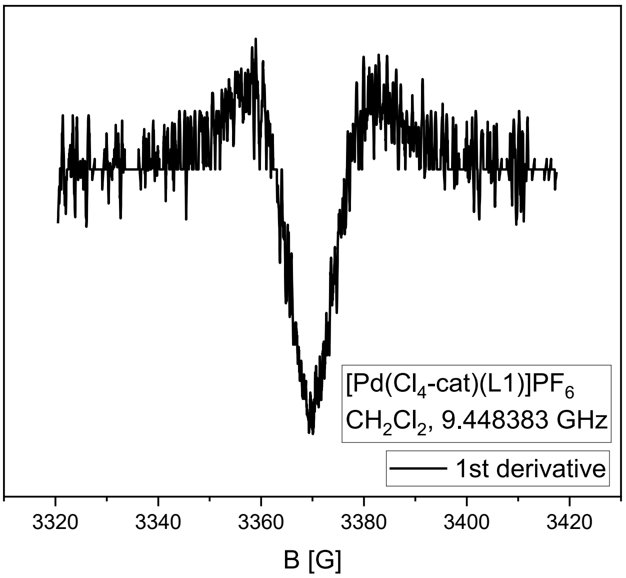

Figure S61: First derivate of the X-band EPR spectrum (CH_2Cl_2) of [Pd(Cl_4-cat)(L1)]PF_6.

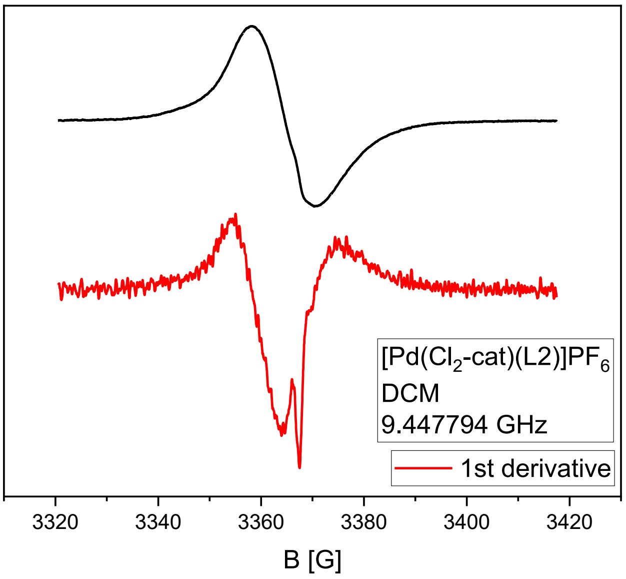

Figure S62: X-band EPR spectrum (CH_2Cl_2) and the first derivative of [Pd(Cl_2-cat)(L2)]PF_6. The asymmetry/shoulder in the signal is confirmed by the first derivate.

S5.4 Solid state spectra

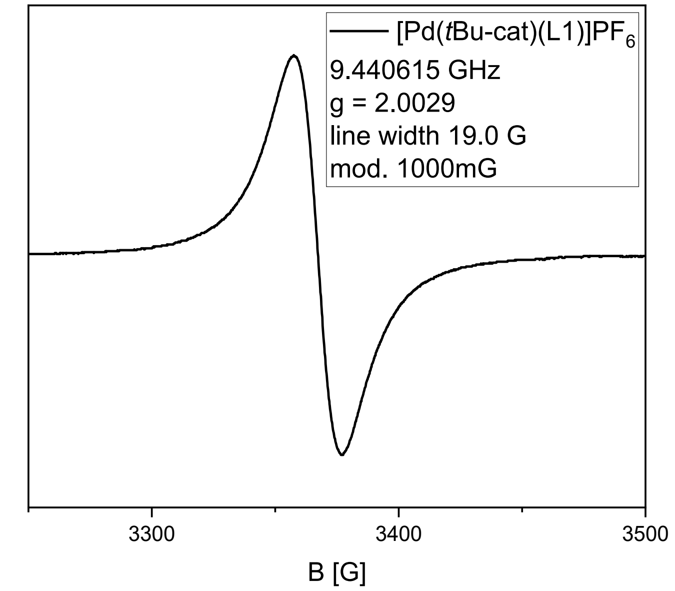

Figure S63: X-band EPR spectrum (solid state) of [Pd(*t*Bu-cat)(L1)]PF_6.

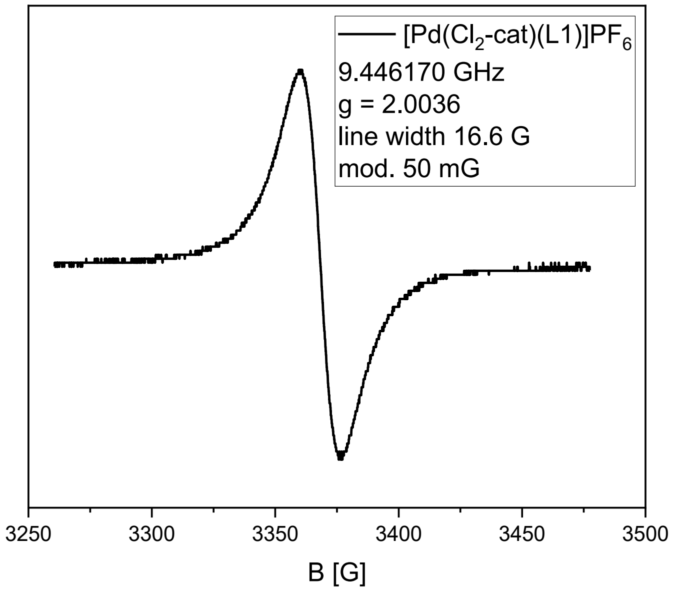

Figure S64: X-band EPR spectrum (solid state) of [Pd(Cl_2-cat)(L1)]PF_6.

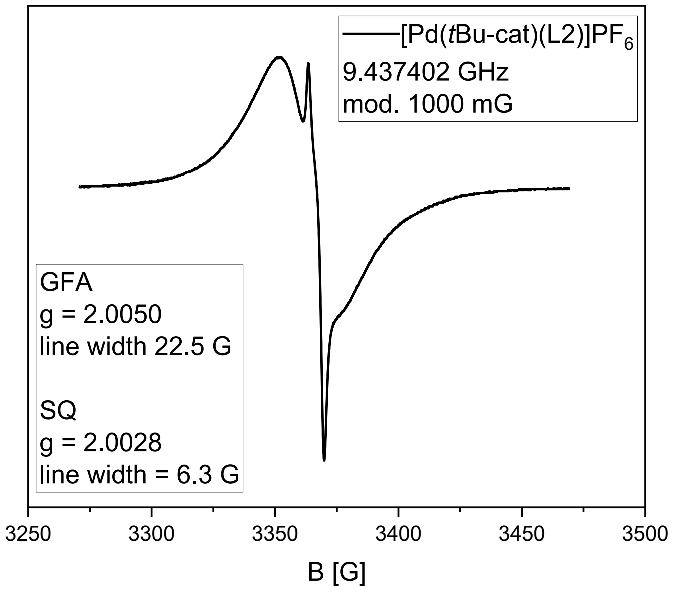

Figure S65: X-band EPR spectrum (solid state) of [Pd(*t*Bu-cat)(L2)]PF_6.

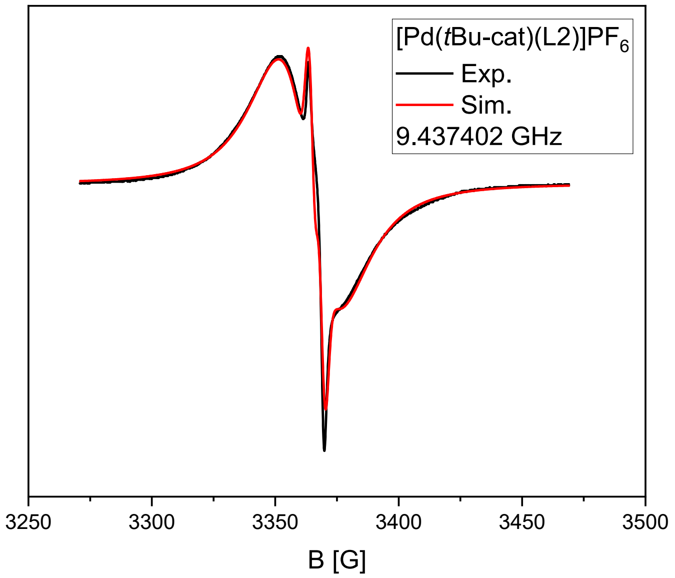

Figure S66: Experimental X-band EPR spectrum (solid state) and Simulation of [Pd(*t*Bu-cat)(L2)]PF_6. Simulation parameters (GFA): ca.98.5%, g=2.0051, lw= 1.4,3.3mT. Simulation parameters (SQ): ca. 1.5%, g=2.0032, a(1H)=0.39 MHz, lw=0.04,0.12mT.

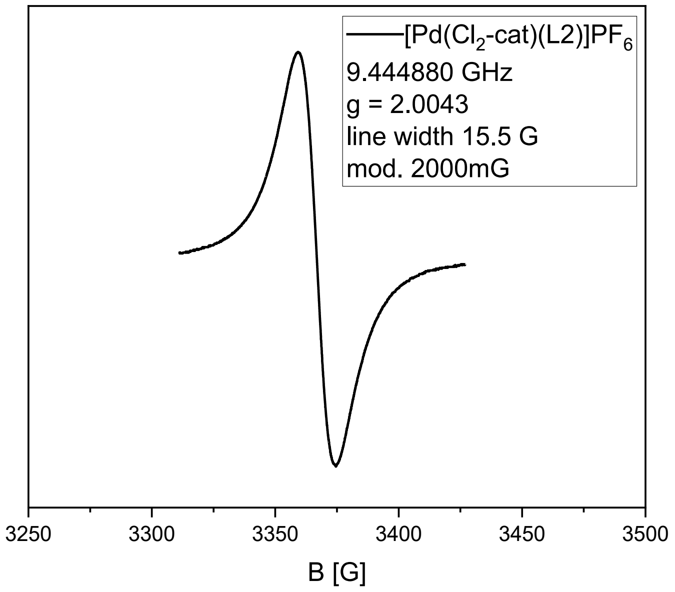

Figure S67: X-band EPR spectrum (solid state) of $[Pd(Cl_2\text{-cat})(L2)]PF_6$.

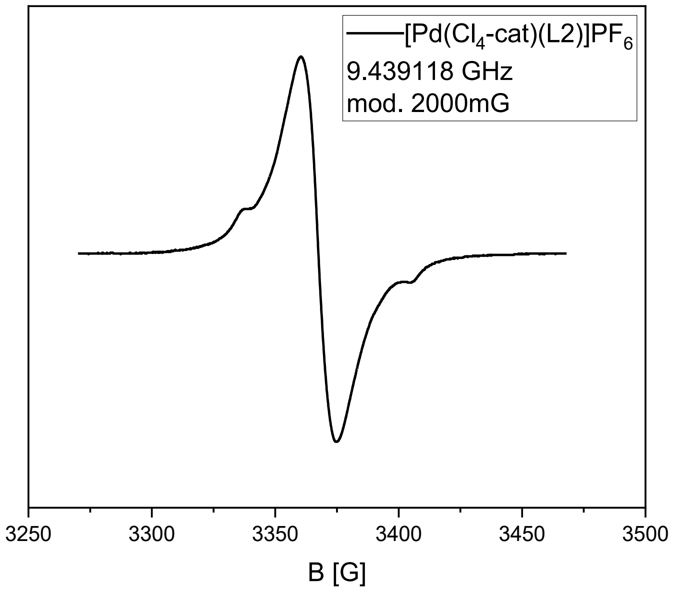

Figure S68: X-band EPR spectrum (solid state) of $[Pd(Cl_4\text{-cat})(L2)]PF_6$. Orthorhombic anisotropy, g_1=2.012, g_2=2.003, g_3=1.980.

S6 UV-vis spectroscopy

S6.1 Spectra in nm

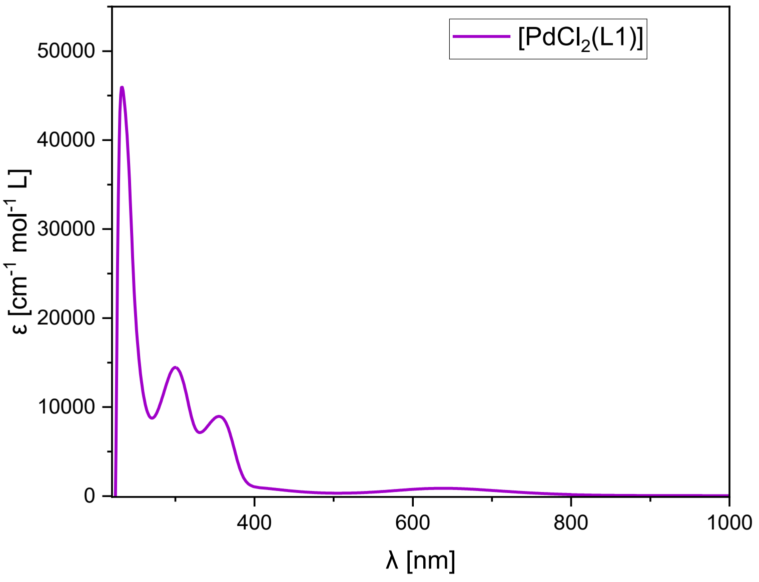

Figure S69: UV-vis spectrum (CH_2Cl_2) of $[PdCl_2(L1)]$.

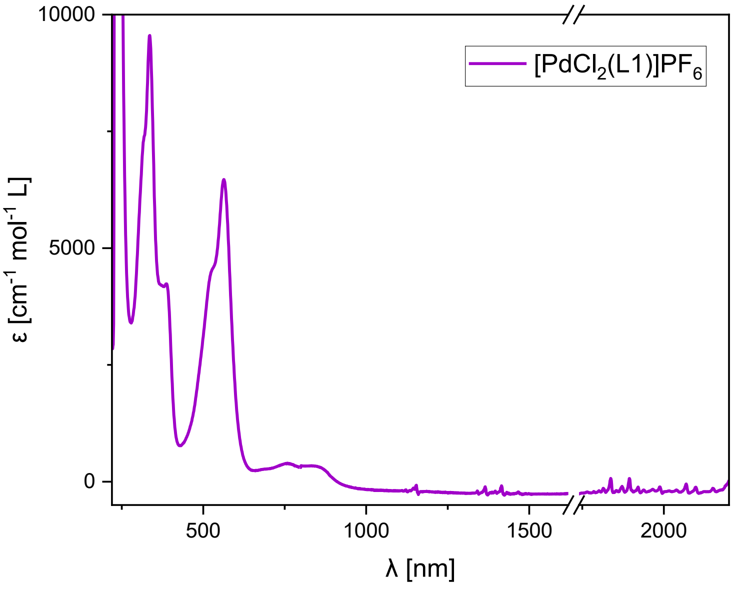

Figure S70: UV-vis spectrum (CH_2Cl_2) of $[PdCl_2(L1)]PF_6$.

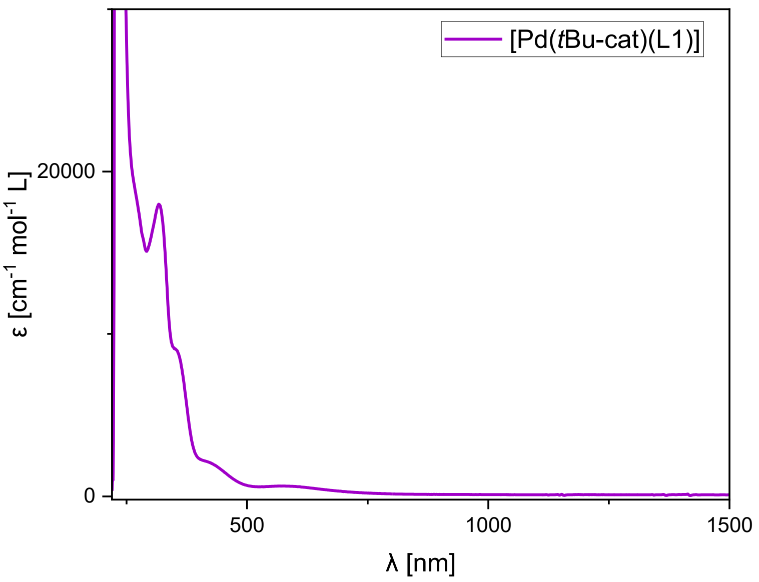

Figure S71: UV-vis spectrum (CH_2Cl_2) of [Pd(tBu-cat)(L1)].

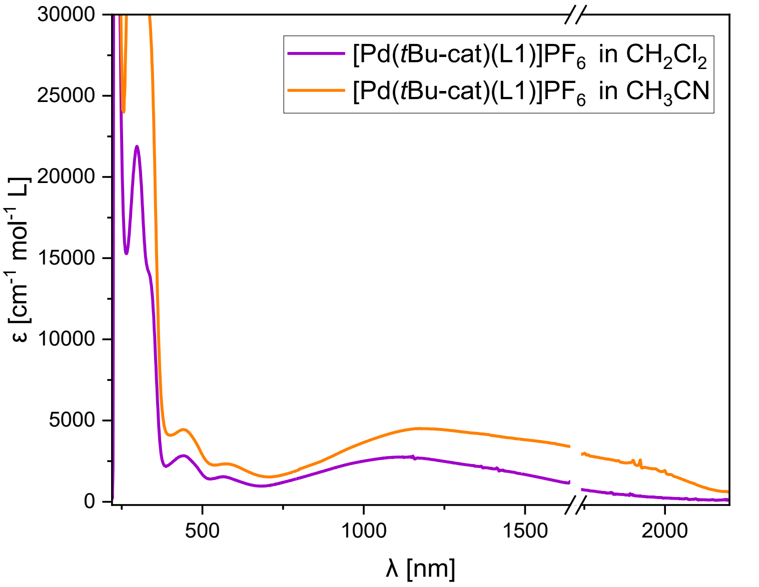

Figure S72: UV-vis spectra of [Pd(tBu-cat)(L1)]PF_6.

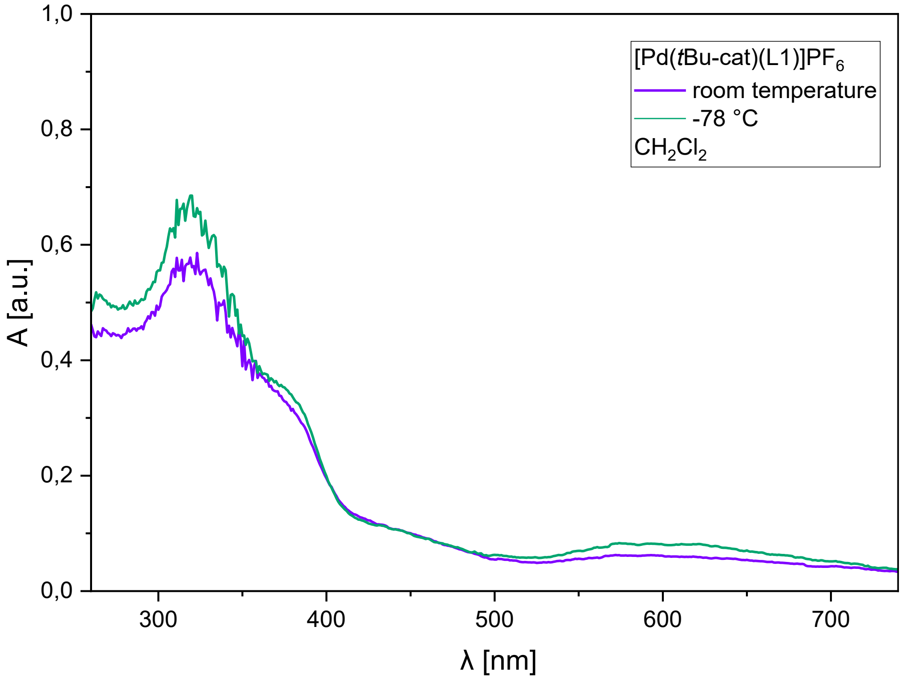

Figure S73: UV-vis spectra (CH_2Cl_2) of [Pd(tBu-cat)(L1)]PF_6 at different temperatures.

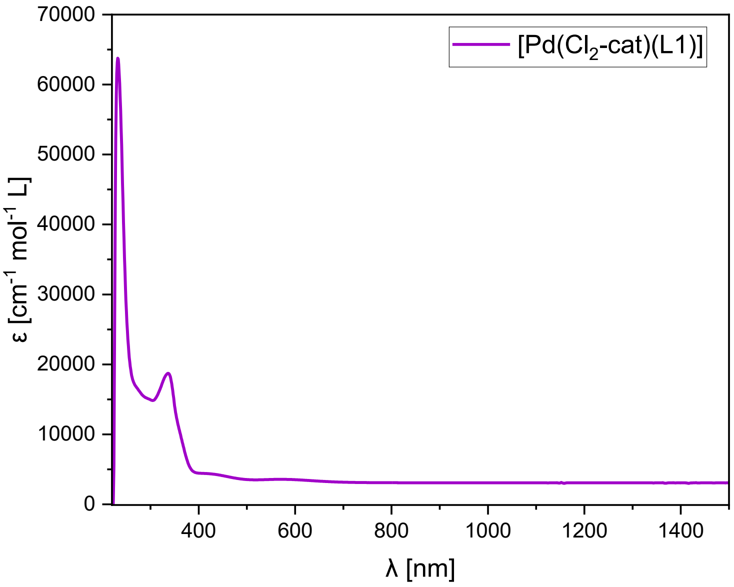

Figure S74: UV-vis spectrum (CH_2Cl_2) of [Pd(Cl_2-cat)(L1)].

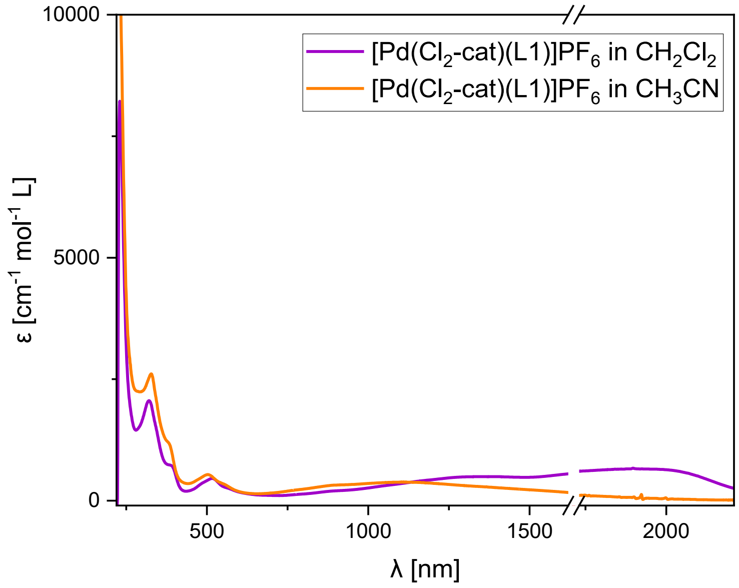

Figure S75: UV-vis spectra of [Pd(Cl_2-cat)(L1)]PF_6.

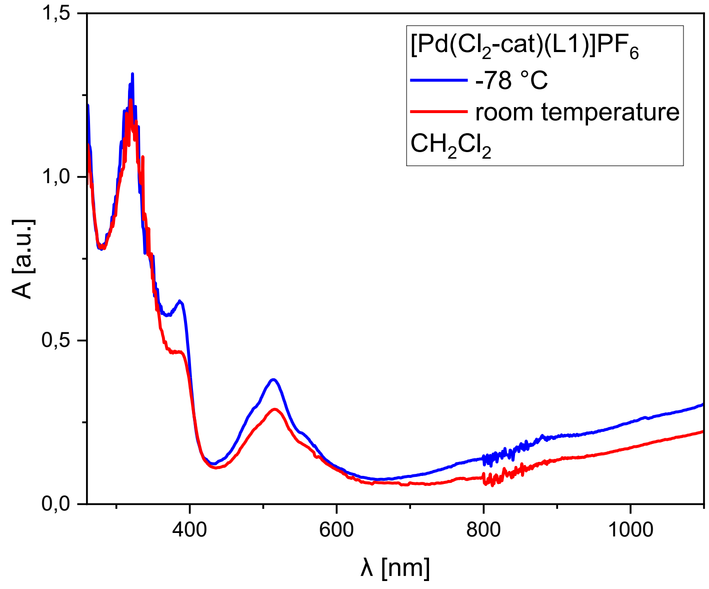

Figure S76: UV-vis spectra (CH_2Cl_2) of [Pd(Cl_2-cat)(L1)]PF_6 at different temperatures.

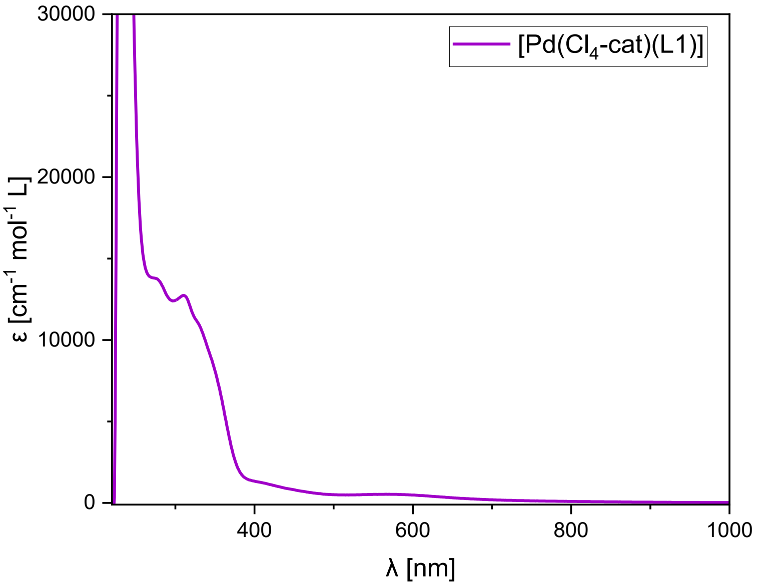

Figure S77: UV-vis spectrum (CH_2Cl_2) of [Pd(Cl_4-cat)(L1)].

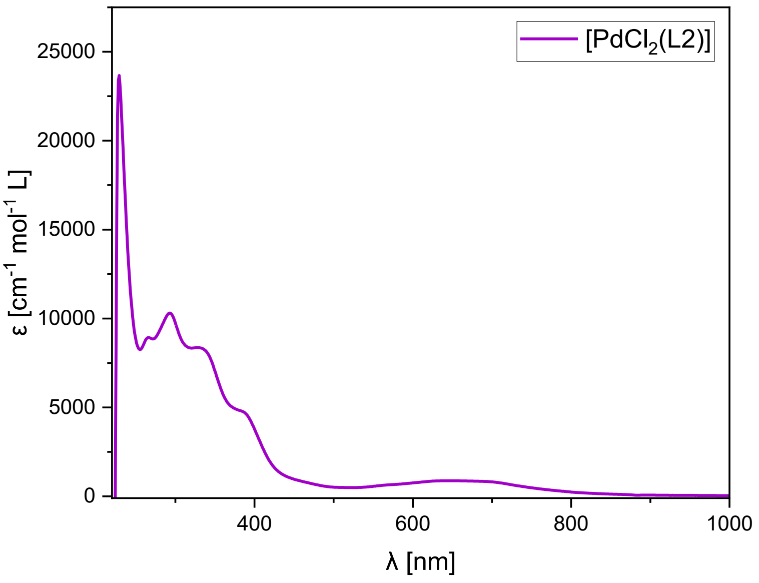

Figure S78: UV-vis spectrum (CH_2Cl_2) of [$PdCl_2$(L2)].

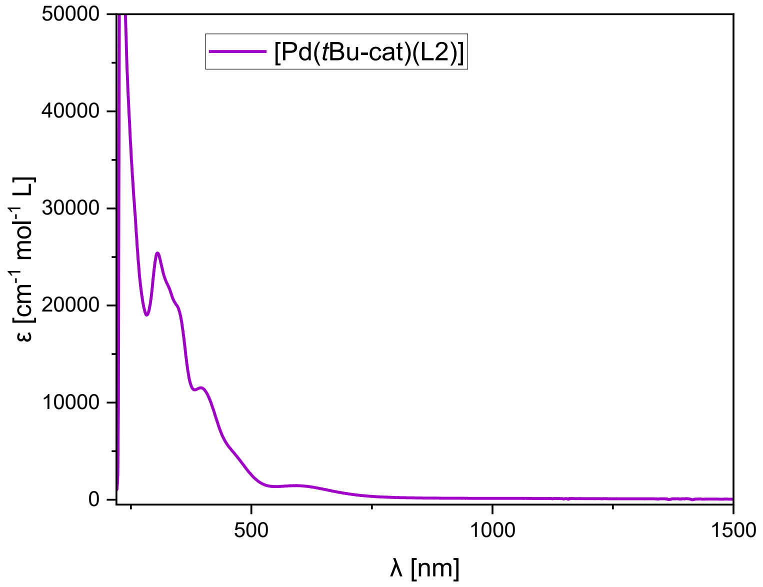

Figure S79: UV-vis spectrum (CH_2Cl_2) of [Pd(tBu-cat)(L2)].

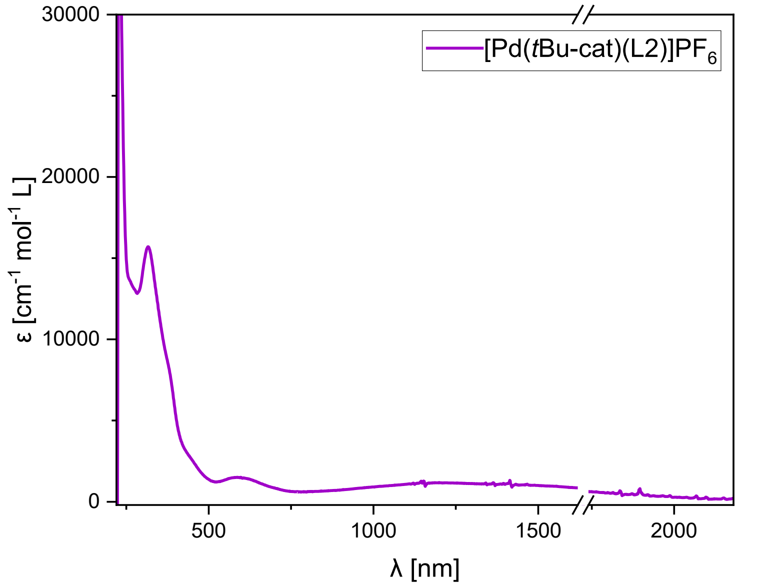

Figure S80: UV-vis spectrum (CH_2Cl_2) of [Pd(tBu-cat)(L2)]PF_6.

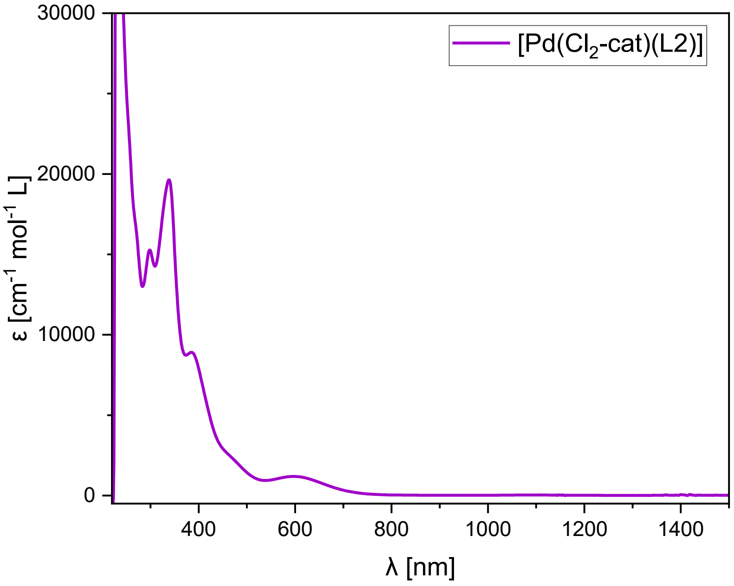

Figure S81: UV-vis spectrum (CH_2Cl_2) of [Pd(Cl_2-cat)(L2)].

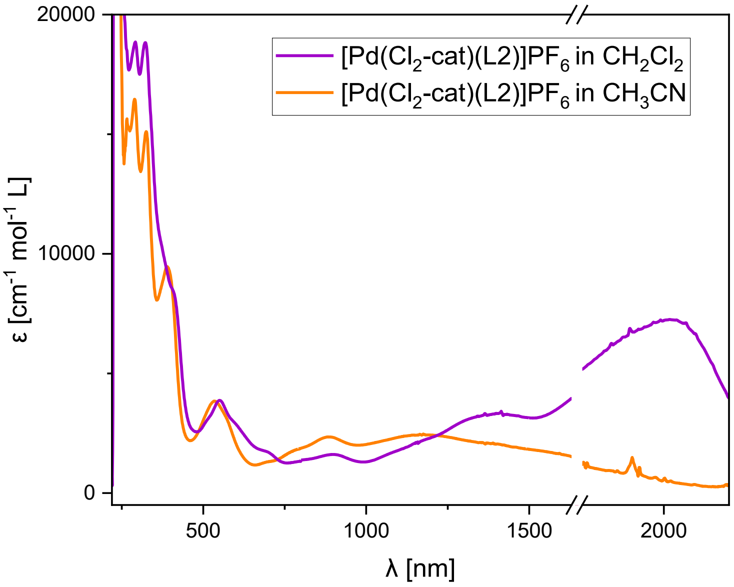

Figure S82: UV-vis spectra of [Pd(Cl_2-cat)(L2)]PF_6.

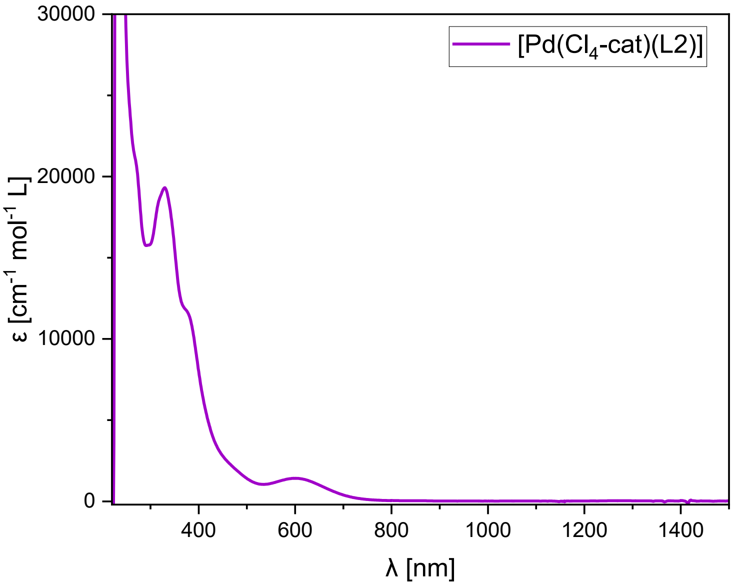

Figure S83: UV-vis spectrum (CH_2Cl_2) of [Pd(Cl_4-cat)(L2)].

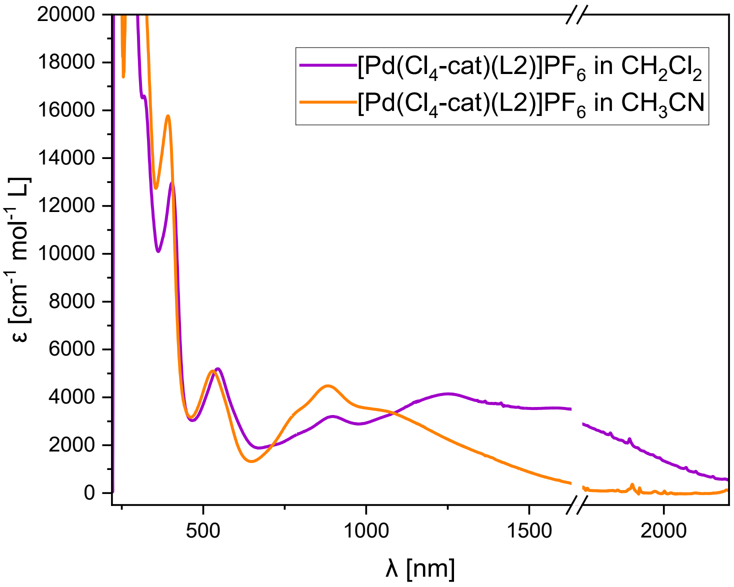

Figure S84: UV-vis spectra of [Pd(Cl_4-cat)(L2)]PF_6.

S6.2 Tabular summary

Table S3: Observed IVCT bands of the oxidised complexes.

Compound	Solvent	λ [nm] (ε [10^3 M^{-1} cm^{-1}])	*E* [cm^{-1}]	*E* [kJ/mol]	*E* [eV]
$[Pd(t\text{Bu-cat})(L1)]^+$	CH_2Cl_2	1110 (2.76)	9009	107.8	1.12
$[Pd(Cl_2\text{-cat})(L1)]^+$	CH_2Cl_2	1365 (0.50), 1940 (0.65)	7326, 5155	87.6, 61.7	0.91, 0.64
$[Pd(t\text{Bu-cat})(L2)]^+$	CH_2Cl_2	1200 (1.18)	8333	99.7	1.03
$[Pd(Cl_2\text{-cat})(L2)]^+$	CH_2Cl_2	1381 (3.28), 2018 (7.26)	7241, 4955	86.6, 59.3	0.90, 0.61
$[Pd(Cl_4\text{-cat})(L2)]^+$	CH_2Cl_2	1253 (4.14), (1594 (3.55), shoulder)	7981, 6274	95.5, 75.0	0.99, 0.78
$[Pd(t\text{Bu-cat})(L1)]^+$	CH_3CN	1169 (4.50)	8554	102.3	1.06
$[Pd(Cl_2\text{-cat})(L1)]^+$	CH_3CN	1118 (0.38)	8945	107.0	1.11
$[Pd(Cl_2\text{-cat})(L2)]^+$	CH_3CN	1175 (2.47)	8511	101.8	1.06
$[Pd(Cl_4\text{-cat})(L2)]^+$	CH_3CN	1069 (3.40)	9355	111.9	1.16

S6.3 Interpretation of IVCT bands by Marcus-Hush model

The Marcus-Hush model[34a,35,34b] allows to interpret the IVCT bands in terms of only a few parameters. The main idea of the model is the existence of a reaction coordinate that connects the equilibrium structures of either state. Each state is represented by a harmonic diabatic potential energy curve along the reaction coordinate, see Figure S85. The results of the ab initio calculations, Fig. 4d of the main article, confirm such a picture qualitatively. The two curves are characterised by the relative energy position of the minima, ΔG^0, and the reorganisation energy λ, which is the energy by which the energy curve has increased in either diabatic state, when traversing along the reaction coordinate from its minimum to the point, where the other state has its minimum. For the sake of simplicity, equal reorganisation energies are assumed for both states. In addition, there is also a coupling constant V, which determines the probability of the hopping process between the two states. This constant is not discussed in detail here, at the end of this section, we will shortly discuss the expected (thermal) charge transfer rates according to the Marcus model.

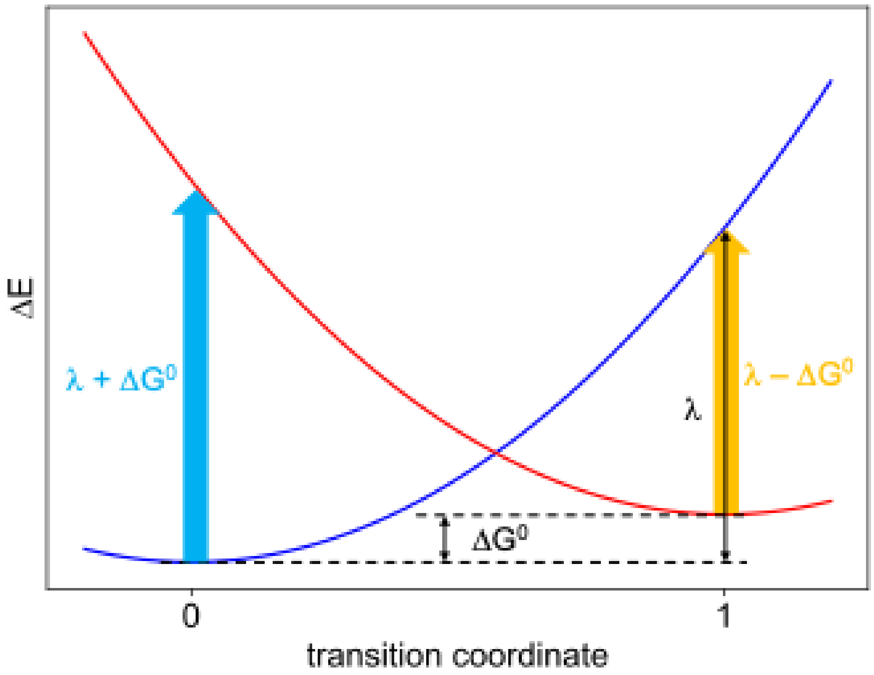

Figure S85: Illustration of the Marcus-Hush model (assuming identical reorganisation energies for both states). The vertical excitations at each minimum are also indicated.

As indicated in Figure S85, if both transitions are observable, the model allows to estimate the reorganisation energy λ as the average vertical IVCT excitation energy, and the energy difference ΔG^0 as half the difference of these energies. Furthermore, the solvent shift of the IVCT energies can be interpreted as the differential solvent stabilisation energy. It should be noted, however, that the IVCT bands are very broad and the position of the maximum might be slightly shifted from the vertical excitation energy. This can particularly happen, if the transition coordinate includes a significant contribution from "hard" normal modes (with $\hbar\omega \gg kT$), which then only contribute their 0-0 transition to the Franck-Condon profile. Therefore, the interpretation of the IVCT spectra should be interpreted with care.

For $[Pd(Cl_2\text{-cat})(L1)]^+$ in CH_2Cl_2 solvent, absorption maxima are found that correspond to 87.6 kJ mol^{-1} and 61.7 kJ mol^{-1}. Assuming comparable reorganisation energies, we get λ = 75 kJ mol^{-1} and ΔG^0 = 13 kJ mol^{-1}. This value is a bit larger than that estimated from the fit of

the EPR spectra (5.2 kJ mol^{-1}), which may be rectified by assuming slightly different reorganisation energies for the two states.

In acetonitrile solvent, only a single IVCT band blue-shifted to 107.0 kJ mol^{-1} is found. This can be interpreted as a stabilisation of the diguanidine-centred radical by approximately 19 kJ mol^{-1} (the stabilisation predicted by the dCOSMO-RS model is 11 kJ mol^{-1}, see Tab. 2 of the main text). The relatively large energy difference of at least 24 kJ mol^{-1} (using the EPR-derived value for CH_2Cl_2 and the solvent shift discussed before) will lead to a very small fraction of the semiquinonate and explains the absence of a second IVCT peak in acetonitrile solvent.

The spectra of $[Pd(Cl_2\text{-cat})(L2)]^+$ are very similar to those of the compound with L1 ligand. The two radical species are both contributing when using CH_2Cl_2 as solvent and two IVCT bands are visible, with band maxima corresponding to 86.6 and 59.3 kJ mol^{-1}. From this, λ = 73 kJ mol^{-1} and a ΔG^0 = 13.7 kJ mol^{-1} can be extracted, very similar to the L1 case. Upon changing the solvent to CH_2CN, the IVCT band of the diguanidino-centred radical is blue-shifted by 15.2 kJ mol^{-1}, while the other IVCT band vanishes.

$[Pd(Cl_4\text{-cat})(L2)]^+$ shows an IVCT peak with a broad shoulder in CH_2Cl_2, but this feature cannot be the IVCT of the semiquinonate, which is expected at sufficiently higher energy thus suppressing any thermal occupation. We assign the peak at 95.5 kJ mol^{-1} to the diguanidino radical, which is a shift of 9 kJ mol^{-1} relative to the L1 species $[Pd(Cl_2\text{-cat})(L1)]^+$ in the same solvent, in line with the computed stabilization of the diguanidine-based radical with change of the catecholate ligand. The solvent stabilisation of 16 kJ mol^{-1} when moving to acetonitrile is also in line with calculations (14 kJ mol^{-1}).

For $[Pd(t\text{Bu-cat})(L1)]^+$ again only a single IVCT band is observed, but in this case it is expected to originate from a semiquinonate radical. In accordance with that, changing to the more polar acetonitrile solvent leads to a red shift of the IVCT band. The measurements give a shift of 5 kJ mol^{-1} (computed 10 kJ mol^{-1}).

As indicated above, we have so far not considered the magnitude of the coupling between the two states. The computations seem to indicate a rather small coupling, as no indications of an avoided crossing were found at the Hartree-Fock and DFT level of theory. However, we have not yet scanned the transition path with sufficient detail. In terms of the experimental observations, it appears that the system instantaneously adapts to the thermodynamic equilibrium. A quick estimate based on the Marcus rate equation[35]

$$k = \frac{2\pi}{\hbar}|V|^2\sqrt{\frac{1}{4\pi\lambda kT}}\exp\left[-\frac{(\Delta G^0+\lambda)^2}{4\lambda kT}\right]$$

shows that, due to the very low barrier ($< \frac{\lambda}{4} \approx$ 20 kJ mol^{-1}), even a tiny coupling of $V \sim 1$ cm^{-1} is sufficient to create a transfer rate > 10^3 s^{-1}.

S7 Ab initio calculations

Table S4: Computed energy differences (kJ mol^{-1}) between the guanidino radical and the dioxolene radical form of the Pd complexes. Negative sign indicates more stable guanidino radical form.

	$[Pd(Cl_4$-cat)(L1)$]^+$	$[Pd(Cl_2$-cat)(L1)$]^+$	[Pd(*t*Bu-cat)(L1)$]^+$	$[Pd(Cl_4$-cat)(L2)$]^+$	$[Pd(Cl_2$-cat)(L2)$]^+$	[Pd(*t*Bu-cat)(L2)$]^+$
ΔE(DFT)[a]	28.9	54.7	84.1	40.3	63.8	93.0
ΔE(CC)[b]	16.3	38.7	62.7	21.9	42.1	66.9
$\Delta\Delta$E(ZPE, vac)[c]	0.1	–0.4	[–1.3] [k]	0.0	–0.7	[–1.1] [k]
$\Delta\Delta$E(ZPE, solv)[d]	0.5	–0.2	–1.3	–0.4	0.3	–1.1
$\Delta\Delta\mu$(298.15 K, vac) [e]	–2.9	–3.3	[–3.0] [k]	–1.6	–1.7	[–2.2] [k]
$\Delta\Delta\mu$(298.15 K, solv) [f]	–2.4	–2.9	–3.0	–1.9	–1.6	–2.2
$\Delta\Delta$E(sol,CHX) [g]	–20.4	–25.4	–25.4	–19.7	–22.7	–23.2
$\Delta\Delta$E(sol,DCM) [g]	–40.7	–48.0	–45.8	–43.8	–48.7	–47.2
$\Delta\Delta$E(sol,ACN) [g]	–57.5	–59.2	–55.5	–58.2	–58.7	–53.5
ΔG(est,vac) [h]	13.5	35.0	58.4	20.2	39.0	63.6
ΔG(est,CHX) [i]	–6.0	10.2	32.9	-0.3	18.1	40.4
ΔG(est,DCM) [i]	–26.3	–12.4	12.5	–24.3	–7.9	16.4
ΔG(est,ACN) [i]	–43.1	–23.7	2.9	–38.7	–17.9	10.2
ΔG (exp, DCM)[j]		–5.2	+1.5			+0.7

[a] Energy difference computed by DFT/LC-ωPBE-D3(BJ)/def2-SVP; [b] Energy difference computed by PNO-LCCSD(T)-F12a/cc-pVDZ-F12; [c] Zero-point vibrational energy contribution at DFT/LC-ωPBE-D3(BJ)/def2-SVP level; [d] Zero-point vibrational energy contribution at DFT/LC-ωPBE-D3(BJ)/def2-SVP, COSMO(ε=2) level; [e] Chemical potential contribution at 298.15 K, using frequencies at DFT/LC-ωPBE-D3(BJ)/def2-SVP level; [f] Chemical potential contribution at 298.15 K, using frequencies at DFT/LC-ωPBE-D3(BJ)/def2-SVP, COSMO(ε=2) level; [g] Solvation energy contribution at DFT/LC-ωPBE-D3(BJ)/def2-SVP, dCOSMO-RS level using parameter sets for cyclohexane (CHX), dichloromethane (DCM), and acetonitrile (ACN); [f] Best estimates for gas phase energy difference from ΔE(CC) + $\Delta\Delta$E(ZPE, vac) + $\Delta\Delta\mu$(298.15 K, vac); [f] Best estimates for energy difference in solution from ΔE(CC) + $\Delta\Delta$E(ZPE) + $\Delta\Delta\mu$(298.15 K) + $\Delta\Delta$E(sol, X) for solvent X; [j] Values estimated from fits of EPR spectra. [k] Value from COSMO(ε=2) computation, see text.

Detailed results for the relative stabilisation energy of the two radical forms are summarised in Table S4. Computational details have been summarised in section S1.3.

The geometries used are the gas phase structures obtained at the DFT/LC-ωPBE-D3(BJ)/def2 SVP level of theory, except for the *t*Bu-substituted species, for which we had to include COSMO(ε=2) solvation stabilisation contributions to be able to converge to the diguanidino radical local minimum. For all cases, we also computed the structures including solvation effects using COSMO(ε=2) and COSMO($\varepsilon=\infty$). Zero-point vibrational energies were computed at the same level of theory. All structures of the L1-GFA species were minima, only for the semiquinonate radicals of the L2-GFA species, one imaginary eigenvalue was found, corresponding to a distortion of the GFA ligand. We have not further investigated this, the effect on the zero-point energy and chemical potential is minimal.

The comparison with respect to the experimental estimates from the EPR spectra is not fully satisfactory. The stability of the diguanidino radical form $[Pd(Cl_2\text{-cat})(L1)]^+$ is overestimated by the computations, as well as the stability of the semiquinonate form in case of $[Pd(t\text{Bu-cat})(L1)]^+$ and $[Pd(t\text{Bu-cat})(L2)]^+$. A possible reason is the rather large contribution from the solvation energy, which also depends on the predicted charge distribution. Both estimates depend on the density functional theory description of the system. The use of a range-separated functional turned out to be crucial for being able to recover both minima (diguanidino and semiquinonato radical). As can be deduced from table S4, the coupled-cluster description of the energetics was seminal for a correct description of the switching between the stable forms along the series of dioxolene ligands, once solvent effects are taken into account. Chemical accuracy (1 kcal mol^{-1} ≈ 4.183 kJ mol^{-1}) could not yet be reached, however.

The calculated EPR parameters are summarized in Table S5.

Table S5: EPR parameters for $[Pd(Cl_2\text{-cat})(L1)]^+$ obtained from ab initio calculations. Hyperfine coupling constants are given in MHz. Within N2 and N3, N2 refers to the nitrogens, that are oriented slightly closer to the palladium.

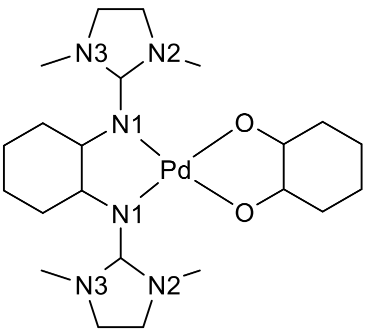

Atom	GFA radical	SQ radical
Pd	13.8	8.4
N1	8.1	-0.5
N2	3.1	0.04
N3	3.3	0.007
g- value (anisotropy)	2.0058 (0.05)	2.0019 (0.02)

S8 X-ray crystallography

S8.1 Comparision of structural data

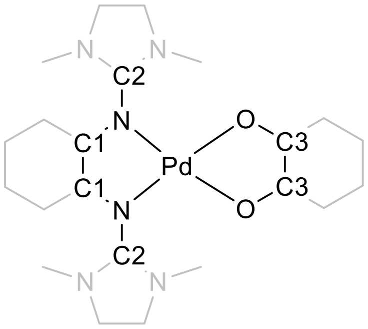

Table S6: Selected bond lengths (in Å) for the neutral Pd complexes from SC-XRD measurements. In the case of [Pd(Cl_2-cat)(L2)], the unit cell contains two different complexes denoted a and b, and a co-crystallized catechole molecule.

	[Pd(Cl_2-cat)(L1)]	[Pd(Cl_4-cat)(L1)]	[Pd(Cl_2-cat)(L2)] a	[Pd(Cl_2-cat)(L2)] b	[Pd(Cl_4-cat)(L2)]
Pd-N	2.017(3)/ 2.047(3)	2.0138(15)/ 2.0130(15)	2.0183(17)/ 2.0264(17)	2.0071(17)/ 2.0282(17)	2.014(2)/ 2.036(2)
Pd-O	1.984(3)/ 2.025(3)	1.9972(13)/ 1.9961(13)	2.0147(14)/ 2.0267(14)	2.0200(14)/ 2.079(14)	2.0046(17)/ 2.0107(18)
C1-N	1.413(5)/ 1.422(5)	1.413(2)/ 1.414(2)	1.423(3)/ 1.414(3)	1.420(3)/ 1.414(3)	1.421(3)/ 1.417(3)
C3-O	1.335(5)/ 1.332(5)	1.321(2)/ 1.321(2)	1.349(2)/ 1.348(2)	1.356(2)/ 1.351(2)	1.317(3)/ 1.339(3)
C1-C1	1.338(6)	1.406(2)	1.400(3)	1.400(3)	1.398(3)
C3-C3	1.424(5)	1.424(3)	1.420(3)	1.408(3)	1.422(4)
C2-N	1.348(5)/ 1.331(5)	1.335(2)/ 1.335(2)	1.351(3)/ 1.340(3)	1.343(3)/ 1.343(3)	1.345(3)/ 1.333(3)

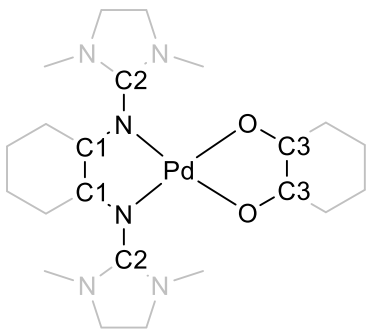

Table S7: Selected bond lengths (in Å) for the oxidised Pd complexes from SC-XRD measurements. In the case of $[Pd(Cl_2\text{-cat})(L2)]^+$, the unit cell contains two independent cations denoted a and b.

	$[Pd(Cl_2\text{-cat})(L1)]^+$	$[Pd(t\text{Bu-cat})(L2)]^{2+}$	$[Pd(Cl_2\text{-cat})(L2)]^+$ a	$[Pd(Cl_2\text{-cat})(L2)]^+$ b	$[Pd(Cl_4\text{-cat})(L2)]^+$
Pd-N	2.021(7)/ 1.990(7)	1.990(3)	2.012(3)/ 2.019(3)	2.001(3)/ 2.008(3)	2.017(4)/ 2.004(4)
Pd-O	2.013(5)/ 2.013(6)	1.987(2)	1.983(2)/ 1.994(2)	1.998(3)/ 2.004(3)	1.992(3)/ 1.985(3)
C1-N	1.385(11)/ 1.359(10)	1.362(4)	1.363(4)/ 1.353(5)	1.367(5)/ 1.364(5)	1.374(6)/ 1.354(6)
C3-O	1.339(11)/ 1.334(12)	1.306(4)	1.342(4)/ 1.346(4)	1.327(5)/ 1.353(5)	1.345(5)/ 1.334(5)
C1-C1	1.430(11)	1.445(6)	1.452(5)	1.433(5)	1.457(6)
C3-C3	1.439(13)	1.421(8)	1.422(5)	1.394(6)	1.412(6)
C2-N	1.386(10)/ 1.382(10)	1.376(4)	1.367(4)/ 1.368(4)	1.365(5)/ 1.371(5)	1.357(5)/ 1.368(5)

Table S8: Metrical oxidation states (MOS) of the oxolene ligands.[36]

	MOS (neutral)	MOS (oxidised)
$[Pd(Cl_2\text{-cat})(L1)]^+$	-1.78 / -1.77	-1.85
$[Pd(Cl_4\text{-cat})(L1)]^+$	-1.70	-
$[Pd(t\text{Bu-cat})(L2)]^{2+}$	-	-1.33*
$[Pd(Cl_2\text{-cat})(L2)]^+$	-1.94 / -2.01	-1.84 / -1.91
$[Pd(Cl_4\text{-cat})(L2)]^+$	-1.76	-1.82

*twofold oxidized species

S8.2 Crystallographic data

Compound	$[PdCl_2(L1)]$	$[PdCl_2(L1)]PF_6$	$[Pd(Cl_2\text{-cat})(L1)]$
Identification code	mo_2024_fk77_0ma	mo_2025_fk118_4_0m	mo_2024_fkpb03_3_0m
Empirical formula	$C_{18}H_{28}Cl_2N_6O_2Pd$	$C_{18}H_{28}Cl_2F_6N_6O_2PPd$	$C_{24}H_{30}Cl_2N_6O_4Pd \cdot 0.75\ CH_2Cl_2$
Formula weight	537.76	682.73	707.53
Temperature/K	100.00	100.00	100.00
Crystal system	monoclinic	triclinic	triclinic
Space group	$P2_1/n$	P-1	P-1
a/Å	11.1313(7)	13.2611(10)	12.7552(14)
b/Å	14.5950(9)	13.9853(10)	13.2163(16)
c/Å	13.1314(8)	15.1673(11)	20.464(3)
α/°	90	72.399(3)	72.527(5)
β/°	91.108(3)	79.673(3)	73.726(4)
γ/°	90	88.308(3)	62.958(4)
Volume/Å^3	2132.9(2)	2636.9(3)	2888.6(6)
Z	4	4	4
ρ_{calc}g/cm^3	1.675	1.720	1.627
μ/mm^{-1}	1.148	1.038	1.009
F(000)	1096.0	1372.0	1438.0
Crystal size/mm^3	0.49 × 0.07 × 0.07	0.21 × 0.19 × 0.1	0.246 × 0.173 × 0.093
Radiation	MoKα (λ = 0.71073)	MoKα (λ = 0.71073)	MoKα (λ = 0.71073)
2Θ range for data collection/°	4.172 to 56.704	3.842 to 54	3.764 to 54.46
Index ranges	-14 ≤ h ≤ 14, -19 ≤ k ≤ 19, -17 ≤ l ≤ 17	-16 ≤ h ≤ 16, -17 ≤ k ≤ 17, -19 ≤ l ≤ 19	-16 ≤ h ≤ 16, -17 ≤ k ≤ 16, -26 ≤ l ≤ 26
Reflections collected	117023	131007	75691
Independent reflections	5328 [R_{int} = 0.0737, R_{sigma} = 0.0262]	11492 [R_{int} = 0.0386, R_{sigma} = 0.0180]	12837 [R_{int} = 0.0958, R_{sigma} = 0.0696]
Data/restraints/parameters	5328/3/306	11492/299/733	12837/0/706
Goodness-of-fit on F^2	1.060	1.095	1.051
Final R indexes [I>=2σ (I)]	R_1 = 0.0304, wR_2 = 0.0740	R_1 = 0.0269, wR_2 = 0.0652	R_1 = 0.0493, wR_2 = 0.1128
Final R indexes [all data]	R_1 = 0.0359, wR_2 = 0.0769	R_1 = 0.0321, wR_2 = 0.0699	R_1 = 0.0608, wR_2 = 0.1199
Largest diff. peak/hole / e Å^{-3}	0.88/-0.46	1.26/-0.46	1.71/-1.14

Compound	$[Pd(Cl_2\text{-cat})(L1)]PF_6$	$[Pd(Cl_4\text{-cat})(L1)]$	$[PdCl_2(L2)]$
Identification code	mo_2025_fk111_3m	mo_2025_fk116_0m	mo_2024_fkpb19_1ma
Empirical formula	$C_{24}H_{30}Cl_2F_6N_6O_4PPd$	$C_{24}H_{28}Cl_4N_6O_4Pd$	$C_{28}H_{30}Cl_4N_6O_2Pd$
Formula weight	788.81	712.72	730.78
Temperature/K	100.00	100.00	100.00
Crystal system	orthorhombic	monoclinic	monoclinic
Space group	Pbca	$P2_1/n$	$P2_1/n$
a/Å	18.781(2)	11.2891(5)	12.2882(14)
b/Å	12.9338(15)	11.5983(5)	14.8310(15)
c/Å	24.859(3)	21.9259(9)	16.847(2)
α/°	90	90	90
β/°	90	94.216(2)	100.829(4)
γ/°	90	90	90
Volume/$Å^3$	6038.6(12)	2863.1(2)	3015.6(6)
Z	8	4	4
$\rho_{calc}g/cm^3$	1.735	1.653	1.610
μ/mm^{-1}	0.924	1.064	1.007
F(000)	3176.0	1440.0	1480.0
Crystal size/mm^3	0.14 × 0.07 × 0.04	0.36 × 0.17 × 0.08	0.23 × 0.14 × 0.1
Radiation	MoKα (λ = 0.71073)	MoKα (λ = 0.71073)	MoKα (λ = 0.71073)
2Θ range for data collection/°	3.93 to 52.352	3.946 to 55	3.784 to 51.994
Index ranges	-23 ≤ h ≤ 23, -15 ≤ k ≤ 16, -30 ≤ l ≤ 30	-14 ≤ h ≤ 14, -15 ≤ k ≤ 15, -28 ≤ l ≤ 28	-15 ≤ h ≤ 15, -18 ≤ k ≤ 18, -20 ≤ l ≤ 20
Reflections collected	61175	175480	87151
Independent reflections	6003 [R_{int} = 0.2197, R_{sigma} = 0.1079]	6551 [R_{int} = 0.0871, R_{sigma} = 0.0245]	5930 [R_{int} = 0.1181, R_{sigma} = 0.0447]
Data/restraints/parameters	6003/302/458	6551/0/358	5930/0/349
Goodness-of-fit on F^2	1.009	1.064	1.042
Final R indexes [I>=2σ (I)]	R_1 = 0.0706, wR_2 = 0.1693	R_1 = 0.0250, wR_2 = 0.0566	R_1 = 0.0401, wR_2 = 0.0937
Final R indexes [all data]	R_1 = 0.1434, wR_2 = 0.2231	R_1 = 0.0276, wR_2 = 0.0584	R_1 = 0.0480, wR_2 = 0.0992
Largest diff. peak/hole / e $Å^{-3}$	0.90/-1.45	0.53/-0.51	1.18/-0.65

Compound	[Pd(*t*Bu-cat)(L2)]$(PF_6)_2$	[Pd(Cl_2-cat)(L2)]	[Pd(Cl_2-cat)(L2)]PF_6
Identification code	mo_2025_fklh06_3_0ma	mo_2025_fkpb19_1_0m	mo_2025_fkfb04_4_0m
Empirical formula	$C_{37}H_{39}F_{12}N_6O_4P_2Pd$ ·1.4 $C_6H_4F_2$	2 $C_{33}H_{30}Cl_2N_6O_4Pd$ ·2 $C_6H_4Cl_2O_2$ ·2 CH_2Cl_2	$C_{33}H_{30}Cl_2F_6N_6O_4PPd$ ·1.5 $C_6H_4F_2$
Formula weight	1187.81	2031.69	1196.39
Temperature/K	100.00	100.00	100.00
Crystal system	monoclinic	triclinic	triclinic
Space group	C2/c	P-1	P-1
a/Å	16.2857(15)	14.9318(7)	11.8888(8)
b/Å	29.230(4)	16.6707(8)	19.5285(14)
c/Å	11.6162(12)	19.7730(7)	23.0636(16)
α/°	90	73.984(2)	82.053(3)
β/°	102.606(3)	86.035(2)	76.208(3)
γ/°	90	64.032(2)	81.176(2)
Volume/Å^3	5396.3(10)	4245.7(3)	5109.8(6)
Z	4	2	4
ρ_{calc}g/cm^3	1.462	1.589	1.555
μ/mm^{-1}	0.500	0.869	0.590
F(000)	2401.0	2056.0	2413.0
Crystal size/mm^3	0.45 × 0.076 × 0.044	0.44 × 0.112 × 0.086	0.38 × 0.27 × 0.25
Radiation	MoKα (λ = 0.71073)	MoKα (λ = 0.71073)	MoKα (λ = 0.71073)
2Θ range for data collection/°	4.172 to 54	3.99 to 55	2.65 to 54
Index ranges	-20 ≤ h ≤ 20, -37 ≤ k ≤ 37, -14 ≤ l ≤ 14	-19 ≤ h ≤ 19, -21 ≤ k ≤ 21, -25 ≤ l ≤ 25	-15 ≤ h ≤ 15, -24 ≤ k ≤ 24, -29 ≤ l ≤ 29
Reflections collected	107032	260189	241742
Independent reflections	5899 [R_{int} = 0.1161, R_{sigma} = 0.0404]	19490 [R_{int} = 0.0901, R_{sigma} = 0.0351]	22277 [R_{int} = 0.0560, R_{sigma} = 0.0262]
Data/restraints/parameters	5899/36/305	19490/0/1052	22277/963/1303
Goodness-of-fit on F^2	1.039	1.029	1.077
Final R indexes [I>=2σ (I)]	R_1 = 0.0516, wR_2 = 0.1375	R_1 = 0.0298, wR_2 = 0.0680	R_1 = 0.0567, wR_2 = 0.1506
Final R indexes [all data]	R_1 = 0.0631, wR_2 = 0.1431	R_1 = 0.0369, wR_2 = 0.0727	R_1 = 0.0669, wR_2 = 0.1663
Largest diff. peak/hole / e Å^{-3}	1.19/-0.72	1.26/-0.64	1.16/-1.17

Compound	$[Pd(Cl_4\text{-cat})(L2)]$	$[Pd(Cl_4\text{-cat})(L2)]PF_6$
Identification code	mo_2024_fkpb18_0m	mo_2025_fk107_3b_0ma
Empirical formula	$C_{34}H_{30}Cl_6N_6O_4Pd$	$C_{42}H_{34}Cl_4F_9N_6O_4PPd$
Formula weight	905.74	1136.92
Temperature/K	100.00	100.00
Crystal system	triclinic	monoclinic
Space group	P-1	$P2_1/n$
a/Å	12.0717(8)	9.9296(7)
b/Å	13.0018(10)	17.7180(12)
c/Å	13.2648(10)	27.3097(18)
α/°	104.841(3)	90
β/°	114.810(3)	94.490(2)
γ/°	92.562(3)	90
Volume/Å^3	1799.1(2)	4789.9(6)
Z	2	4
ρ_{calc}g/cm^3	1.672	1.577
μ/mm^{-1}	1.010	0.726
F(000)	912.0	2280.0
Crystal size/mm^3	0.48 × 0.09 × 0.055	0.45 × 0.11 × 0.08
Radiation	MoKα (λ = 0.71073)	Mo Kα (λ = 0.71073)
2Θ range for data collection/°	3.774 to 53.998	3.774 to 51.998
Index ranges	-15 ≤ h ≤ 15, -16 ≤ k ≤ 16, -16 ≤ l ≤ 16	-12 ≤ h ≤ 12, -21 ≤ k ≤ 21, -33 ≤ l ≤ 33
Reflections collected	76875	210205
Independent reflections	7863 [R_{int} = 0.0993, R_{sigma} = 0.0501]	9401 [R_{int} = 0.1009, R_{sigma} = 0.0291]
Data/restraints/parameters	7863/0/466	9401/144/605
Goodness-of-fit on F^2	1.033	1.089
Final R indexes [I>=2σ (I)]	R_1 = 0.0354, wR_2 = 0.0865	R_1 = 0.0547, wR_2 = 0.1408
Final R indexes [all data]	R_1 = 0.0393, wR_2 = 0.0902	R_1 = 0.0702, wR_2 = 0.1527
Largest diff. peak/hole / e Å^{-3}	1.23/-0.61	2.12/-1.14

S9 Literature

[1] J. Osterbrink, P. Walter, S. Leingang, H. Pfisterer, E. Kaifer, H.-J. Himmel, *Chem. Eur. J.* **2023**, *29*, e202300514.
[2] U. Jahn, P. Hartmann, I. Dix, P. G. Jones, *Eur. J. Org. Chem.* **2001**, *2001*, 3333.
[3] S. Stoll, A. Schweiger, *J. Magn. Reson.* **2006**, *178*, 42.
[4] *SAINT (APEXIII/IV)*, Bruker AXS GmbH, Karlsruhe, Germany, **2016/2021**.
[5] a) G. M. Sheldrick, *SADABS*, Bruker AXS GmbH, Karlsruhe, Germany, **2004-2014**; b) L. Krause, R. Herbst-Irmer, G. M. Sheldrick, D. Stalke, *J. Appl. Cryst.* **2015**, *48*, 3.
[6] a) G. M. Sheldrick, *SHELXT, Program for Crystal Structure Solution*, University of Göttingen, Germany, **2014-2018**; b) G. M. Sheldrick, *Acta Cryst.* **2015**, *71*, 3.
[7] a) G. M. Sheldrick, *SHELXL-20xx*, University of Göttingen and Bruker AXS GmbH, Karlsruhe, Germany, **2012-2018**; b) W. Robinson, G. M. Sheldrick in: N. W. Isaaks, M. R. Taylor (eds.) (Hrsg.) *Crystallographic Computing 4*. Ch. 22, IUCr and Oxford University Press, Oxford, UK, **1988**; c) G. M. Sheldrick, *Acta Cryst.* **2008**, *64*, 112.
[8] K. A. Peterson, T. B. Adler, H.-J. Werner, *J. Chem. Phys.* **2008**, *128*, 84102.
[9] O. V. Dolomanov, L. J. Bourhis, R. J. Gildea, J. A. K. Howard, H. Puschmann, *J. Appl. Cryst.* **2009**, *42*, 339.
[10] A. Thorn, B. Dittrich, G. M. Sheldrick, *Acta Cryst.* **2012**, *68*, 448.
[11] a) P. van der Sluis, A. L. Spek, *Acta Cryst.* **1990**, *46*, 194; b) A. L. Spek, *Acta Cryst.* **2015**, *71*, 9.
[12] a) A. L. Spek, *PLATON*, Utrecht University, The Netherlands; b) A. L. Spek, *J. Appl. Cryst.* **2003**, *36*, 7.
[13] O. A. Vydrov, G. E. Scuseria, *J. Chem. Phys.* **2006**, *125*, 234109.
[14] a) S. Grimme, J. Antony, S. Ehrlich, H. Krieg, *J. Chem. Phys.* **2010**, *132*, 154104; b) S. Grimme, S. Ehrlich, L. Goerigk, *J. Comput. Chem.* **2011**, *32*, 1456.
[15] S. G. Balasubramani, G. P. Chen, S. Coriani, M. Diedenhofen, M. S. Frank, Y. J. Franzke, F. Furche, R. Grotjahn, M. E. Harding, C. Hättig et al., *J. Chem. Phys.* **2020**, *152*, 184107.
[16] S. Lehtola, C. Steigemann, M. J. Oliveira, M. A. Marques, *SoftwareX* **2018**, *7*, 1.
[17] F. Weigend, R. Ahlrichs, *Phys. Chem. Chem. Phys.* **2005**, *7*, 3297.
[18] F. Weigend, *Phys. Chem. Chem. Phys.* **2006**, *8*, 1057.
[19] C. Holzer, *J. Chem. Phys.* **2020**, *153*, 184115.
[20] D. Andrae, U. Huermann, M. Dolg, H. Stoll, H. Preu, *Theor. Chim. Acta* **1990**, *77*, 123.
[21] A. Klamt, G. Schüürmann, *J. Chem. Soc., Perkin Trans. 2* **1993**, 799.
[22] A. Pausch, *J. Chem. Theory Comput.* **2024**, *20*, 3169.
[23] S. Sinnecker, A. Rajendran, A. Klamt, M. Diedenhofen, F. Neese, *J. Phys. Chem. A* **2006**, *110*, 2235.
[24] Y. Zhao, D. G. Truhlar, *Phys. Chem. Chem. Phys.* **2008**, *10*, 2813.
[25] a) S. Gillhuber, Y. J. Franzke, F. Weigend, *J. Phys. Chem. A* **2021**, *125*, 9707; b) F. Bruder, Y. J. Franzke, F. Weigend, *J. Phys. Chem. A* **2022**, *126*, 5050.
[26] Y. J. Franzke, F. Weigend, *J. Chem. Theory Comput.* **2019**, *15*, 1028.
[27] Y. J. Franzke, R. Treß, T. M. Pazdera, F. Weigend, *Phys. Chem. Chem. Phys.* **2019**, *21*, 16658.
[28] C. Steffen, K. Thomas, U. Huniar, A. Hellweg, O. Rubner, A. Schroer, *J. Comput. Chem.* **2010**, *31*, 2967.
[29] H.-J. Werner, P. J. Knowles, F. R. Manby, J. A. Black, K. Doll, A. Heßelmann, D. Kats, A. Köhn, T. Korona, D. A. Kreplin et al., *J. Chem. Phys.* **2020**, *152*, 144107.

[30] a) Q. Ma, H.-J. Werner, *J. Chem. Theory Comput.* **2018**, *14*, 198; b) Q. Ma, H.-J. Werner, *J. Chem. Theory Comput.* **2021**, *17*, 902; c) Q. Ma, H.-J. Werner, *WIREs Comput. Mol. Sci.* **2018**, *8*.
[31] K. A. Peterson, D. Figgen, M. Dolg, H. Stoll, *J. Chem. Phys.* **2007**, *126*, 124101.
[32] T. A. Halgren, W. N. Lipscomb, *Chem. Phys. Lett.* **1977**, *49*, 225.
[33] C. Eckart, *Phys. Rev.* **1935**, *47*, 552.
[34] a) R. A. Marcus, *J. Chem. Phys.* **1956**, *24*, 966; b) N. S. Hush, *Trans. Faraday Soc.* **1961**, *57*, 557.
[35] R. A. Marcus, N. Sutin, *Biochim. Biophys. Acta - Bioenerg.* **1985**, *811*, 265.
[36] S. N. Brown, *Inorg. Chem.* **2012**, *51*, 1251.
