# Supplementary material for: Bistability in Palladium Complexes with Two Different Redox‐Active Ligands of Orthogonal Charge Regimes
Source: Chemistry. 2025 Nov 4;31(69):e03160. doi: 10.1002/chem.202503160 (PMC12699171; doi:10.1002/chem.202503160)

# checkCIF/PLATON report

Structure factors have been supplied for datablock(s) mo_2024_fk77_0ma

THIS REPORT IS FOR GUIDANCE ONLY. IF USED AS PART OF A REVIEW PROCEDURE FOR PUBLICATION, IT SHOULD NOT REPLACE THE EXPERTISE OF AN EXPERIENCED CRYSTALLOGRAPHIC REFEREE.

No syntax errors found. CIF dictionary Interpreting this report

## Datablock: mo_2024_fk77_0ma

---

```
Bond precision:    C-C = 0.0031 A           Wavelength=0.71073

Cell:              a=11.1313(7)     b=14.5950(9)        c=13.1314(8)
                   alpha=90         beta=91.108(3)      gamma=90
Temperature:       100 K

                 Calculated                     Reported
Volume           2133.0(2)                      2132.9(2)
Space group      P 21/n                         P 1 21/n 1
Hall group       -P 2yn                         -P 2yn
Moiety formula   C18 H28 Cl2 N6 O2 Pd           C18 H28 Cl2 N6 O2 Pd
Sum formula      C18 H28 Cl2 N6 O2 Pd           C18 H28 Cl2 N6 O2 Pd
Mr               537.76                         537.76
Dx,g cm-3        1.675                          1.675
Z                4                              4
Mu (mm-1)        1.148                          1.148
F000             1096.0                         1096.0
F000'            1093.48
h,k,lmax         14,19,17                       14,19,17
Nref             5332                           5328
Tmin,Tmax        0.908,0.923                    0.631,0.746
Tmin'            0.570

Correction method= # Reported T Limits: Tmin=0.631 Tmax=0.746
AbsCorr = MULTI-SCAN

Data completeness= 0.999           Theta(max)= 28.352

                                                         wR2(reflections)=
R(reflections)= 0.0304( 4771)                            0.0769( 5328)

S = 1.060                     Npar= 306
```

The following ALERTS were generated. Each ALERT has the format
**test-name_ALERT_alert-type_alert-level.**
Click on the hyperlinks for more details of the test.

## Alert level C

```
PLAT213_ALERT_2_C Atom C17              has ADP max/min Ratio .....         3.5 prolat
PLAT220_ALERT_2_C NonSolvent   Resd 1  C   Ueq(max)/Ueq(min) Range         3.7 Ratio
PLAT906_ALERT_3_C Large K Value in the Analysis of Variance ......       2.008 Check
```

## Alert level G

```
PLAT002_ALERT_2_G Number of Distance or Angle Restraints on AtSite           6 Note
PLAT172_ALERT_4_G The CIF-Embedded .res File Contains DFIX Records           1 Report
PLAT176_ALERT_4_G The CIF-Embedded .res File Contains SADI Records           6 Report
PLAT178_ALERT_4_G The CIF-Embedded .res File Contains SIMU Records           1 Report
PLAT299_ALERT_4_G Atom Site Occupancy Constrained at ............         0.5 Check
              N6      N6A     C11     C14     C14B    C16     C16B    C19
              H11A    H11B    H11C    H13A    H13B    H13C    H13D    H14A
              H14B    H14C    H14D    H16A    H16B    H16C    H16D    H16E
              H16F    H19A    H19B    H19C
PLAT301_ALERT_3_G Main Residue  Disorder .............(Resd    1)         14% Note
PLAT380_ALERT_4_G Incorrectly? Oriented X(sp2)-Methyl Moiety .....         C15 Check
PLAT413_ALERT_2_G Short Inter XH3 .. XHn     H11C     ..H17C     .        2.04 Ang.
                                        -1/2+x,1/2-y,-1/2+z  =      4_565 Check
PLAT860_ALERT_3_G Number of Least-Squares Restraints ............           3 Note
PLAT912_ALERT_4_G Missing # of FCF Reflections Above STh/L=  0.600          4 Note
PLAT969_ALERT_5_G The 'Henn et al.' R-Factor-gap value ..........       2.552 Note
              Predicted wR2: Based on SigI**2  3.01 or SHELX Weight  7.25
PLAT978_ALERT_2_G Number C-C Bonds with Positive Residual Density.          6 Info
```

0 **ALERT level A** = Most likely a serious problem - resolve or explain
0 **ALERT level B** = A potentially serious problem, consider carefully
3 **ALERT level C** = Check. Ensure it is not caused by an omission or oversight
12 **ALERT level G** = General information/check it is not something unexpected

0 ALERT type 1 CIF construction/syntax error, inconsistent or missing data
5 ALERT type 2 Indicator that the structure model may be wrong or deficient
3 ALERT type 3 Indicator that the structure quality may be low
6 ALERT type 4 Improvement, methodology, query or suggestion
1 ALERT type 5 Informative message, check

It is advisable to attempt to resolve as many as possible of the alerts in all categories. Often the minor alerts point to easily fixed oversights, errors and omissions in your CIF or refinement strategy, so attention to these fine details can be worthwhile. In order to resolve some of the more serious problems it may be necessary to carry out additional measurements or structure refinements. However, the purpose of your study may justify the reported deviations and the more serious of these should normally be commented upon in the discussion or experimental section of a paper or in the "special_details" fields of the CIF. checkCIF was carefully designed to identify outliers and unusual parameters, but every test has its limitations and alerts that are not important in a particular case may appear. Conversely, the absence of alerts does not guarantee there are no aspects of the results needing attention. It is up to the individual to critically assess their own results and, if necessary, seek expert advice.

**Publication of your CIF in IUCr journals**

A basic structural check has been run on your CIF. These basic checks will be run on all CIFs submitted for publication in IUCr journals (*Acta Crystallographica*, *Journal of Applied Crystallography*, *Journal of Synchrotron Radiation*); however, if you intend to submit to *Acta Crystallographica Section C* or *E* or *IUCrData*, you should make sure that full publication checks are run on the final version of your CIF prior to submission.

**Publication of your CIF in other journals**

Please refer to the *Notes for Authors* of the relevant journal for any special instructions relating to CIF submission.

---

**PLATON version of 15/07/2024; check.def file version of 15/07/2024**

**Datablock mo_2024_fk77_0ma** - ellipsoid plot

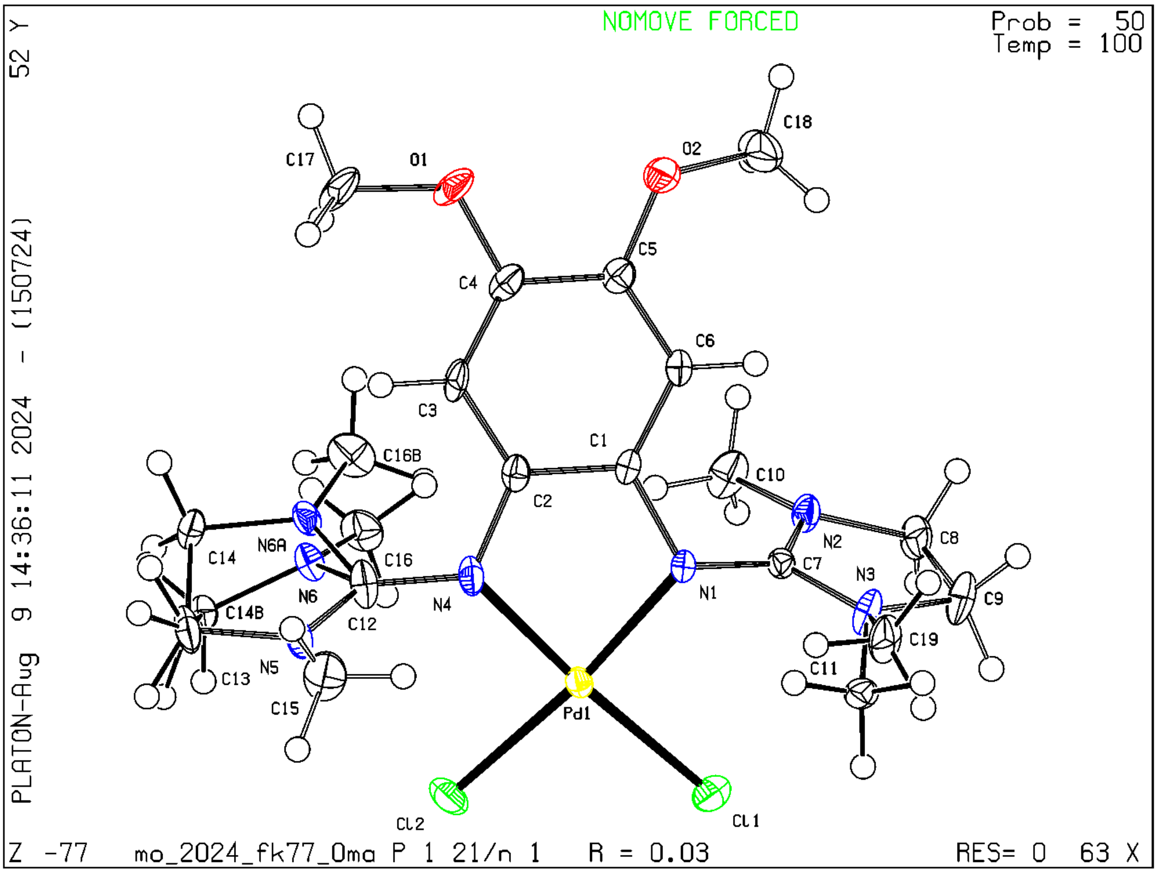

Supplement: Supplementary file 2 — Supporting Information [file CHEM-31-e03160-s002.zip › mo_2024_fk77_0ma_cifreport.pdf]
