# Supplementary material for: Bistability in Palladium Complexes with Two Different Redox‐Active Ligands of Orthogonal Charge Regimes
Source: Chemistry. 2025 Nov 4;31(69):e03160. doi: 10.1002/chem.202503160 (PMC12699171; doi:10.1002/chem.202503160)

checkCIF/PLATON report

Structure factors have been supplied for datablock(s) mo_2024_fkpb03_3_0m

THIS REPORT IS FOR GUIDANCE ONLY. IF USED AS PART OF A REVIEW PROCEDURE FOR PUBLICATION, IT SHOULD NOT REPLACE THE EXPERTISE OF AN EXPERIENCED CRYSTALLOGRAPHIC REFEREE.

No syntax errors found. CIF dictionary Interpreting this report

Datablock: mo_2024_fkpb03_3_0m

Bond precision: C-C = 0.0067 A Wavelength=0.71073

Cell:	a=12.7552(14)	b=13.2163(16)	c=20.464(3)
	alpha=72.527(5)	beta=73.726(4)	gamma=62.958(4)
Temperature:	100 K		

	Calculated	Reported
Volume	2888.6(6)	2888.6(6)
Space group	P -1	P -1
Hall group	-P 1	-P 1
Moiety formula	2(C24 H30 Cl2 N6 O4 Pd), C H2 Cl2 [+ solvent]	C24 H30 Cl2 N6 O4 Pd, 0.5(C H2 Cl2), 0.25[CH2CL2]
Sum formula	C49 H62 Cl6 N12 O8 Pd2 [+ solvent]	C24.75 H31.50 Cl3.50 N6 O4 Pd
Mr	1372.61	707.53
Dx,g cm-3	1.578	1.627
Z	2	4
Mu (mm-1)	0.961	1.009
F000	1396.0	1438.0
F000’	1394.22	
h,k,lmax	16,17,26	16,17,26
Nref	12916	12837
Tmin,Tmax	0.811,0.910	0.604,0.746
Tmin’	0.780	

Correction method= # Reported T Limits: Tmin=0.604 Tmax=0.746
AbsCorr = MULTI-SCAN

Data completeness= 0.994 Theta(max)= 27.230

R(reflections)= 0.0493(10678)   wR2(reflections)= 0.1199(12837)

S = 1.051   Npar= 706

The following ALERTS were generated. Each ALERT has the format
test-name_ALERT_alert-type_alert-level.
Click on the hyperlinks for more details of the test.

Alert level C

```
PLAT906_ALERT_3_C Large K Value in the Analysis of Variance ......      2.612 Check
PLAT911_ALERT_3_C Missing FCF Refl Between Thmin & STh/L=    0.600         12 Report
                0  8  1,   0 10  1,   6 -6  2,   8 -1  2,   9  1  3,   6  3  4,
                7  8  5,   8  9  5,   9 10  5,   4 10  6,  11  7  7,   2  3 14,
PLAT971_ALERT_2_C Check Calcd Resid. Dens.  1.43Ang From Pd1            1.77 eA-3
PLAT971_ALERT_2_C Check Calcd Resid. Dens.  1.21Ang From O33            1.55 eA-3
```

Alert level G

```
FORMU01_ALERT_2_G  There is a discrepancy between the atom counts in the
            _chemical_formula_sum and the formula from the _atom_site* data.
            Atom count from _chemical_formula_sum:C24.75 H31.5 Cl3.5 N6 O4 Pd1
            Atom count from the _atom_site data:  C24.5 H31 Cl3 N6 O4 Pd1
CELLZ01_ALERT_1_G Difference between formula and atom_site contents detected.
CELLZ01_ALERT_1_G ALERT: Large difference may be due to a
            symmetry error - see SYMMG tests
           From the CIF: _cell_formula_units_Z    4
           From the CIF: _chemical_formula_sum  C24.75 H31.50 Cl3.50 N6 O4 Pd
           TEST: Compare cell contents of formula and atom_site data

           atom    Z*formula  cif sites diff
           C         99.00     98.00    1.00
           H        126.00    124.00    2.00
           Cl        14.00     12.00    2.00
           N         24.00     24.00    0.00
           O         16.00     16.00    0.00
           Pd         4.00      4.00    0.00
PLAT041_ALERT_1_G Calc. and Reported SumFormula    Strings  Differ     Please Check
              Calc: C24.50 H31 Cl3 N6 O4 Pd
              Rep.: C24.75 H31.50 Cl3.50 N6 O4 Pd
PLAT042_ALERT_1_G Calc. and Reported MoietyFormula Strings  Differ     Please Check
              Calc: 2(C24 H30 Cl2 N6 O4 Pd), C H2 Cl2
              Rep.: C24 H30 Cl2 N6 O4 Pd, 0.5(C H2 Cl2), 0.25[CH2C
                    L2]
PLAT045_ALERT_1_G Calculated and Reported Z Differ by a Factor ...      0.500 Check
PLAT051_ALERT_1_G Mu(calc) and Mu(cif) Ratio Differs from 1.0 by .       4.72 %
PLAT083_ALERT_2_G SHELXL Second Parameter in WGHT  Unusually Large      14.34 Why ?
PLAT431_ALERT_2_G Short Inter HL..A Contact  Cl31     ..O3        .      3.09 Ang.
                                              1-x,2-y,1-z  =      2_676 Check
PLAT605_ALERT_4_G Largest Solvent Accessible VOID in the Structure        112 A**3
PLAT794_ALERT_5_G Tentative Bond Valency for Pd1       (II)       .      2.22 Info
PLAT794_ALERT_5_G Tentative Bond Valency for Pd2       (II)       .      2.22 Info
PLAT868_ALERT_4_G ALERTS Due to the Use of _smtbx_masks Suppressed          ! Info
PLAT910_ALERT_3_G Missing # of FCF Reflection(s) Below Theta(Min).          4 Note
                1  0  0,   0  1  0,   0  0  1,   0  1  1,
```

```
PLAT912_ALERT_4_G Missing # of FCF Reflections Above STh/L=  0.600         64 Note
PLAT933_ALERT_2_G Number of HKL-OMIT Records in Embedded .res File          1 Note
                2  3 14,
PLAT969_ALERT_5_G The 'Henn et al.' R-Factor-gap value ..........       1.554 Note
              Predicted wR2: Based on SigI**2  7.72 or SHELX Weight 11.41
PLAT978_ALERT_2_G Number C-C Bonds with Positive Residual Density.          2 Info
```

```
   0 ALERT level A = Most likely a serious problem - resolve or explain
   0 ALERT level B = A potentially serious problem, consider carefully
   4 ALERT level C = Check. Ensure it is not caused by an omission or oversight
  18 ALERT level G = General information/check it is not something unexpected

   6 ALERT type 1 CIF construction/syntax error, inconsistent or missing data
   7 ALERT type 2 Indicator that the structure model may be wrong or deficient
   3 ALERT type 3 Indicator that the structure quality may be low
   3 ALERT type 4 Improvement, methodology, query or suggestion
   3 ALERT type 5 Informative message, check
```

It is advisable to attempt to resolve as many as possible of the alerts in all categories. Often the minor alerts point to easily fixed oversights, errors and omissions in your CIF or refinement strategy, so attention to these fine details can be worthwhile. In order to resolve some of the more serious problems it may be necessary to carry out additional measurements or structure refinements. However, the purpose of your study may justify the reported deviations and the more serious of these should normally be commented upon in the discussion or experimental section of a paper or in the "special_details" fields of the CIF. checkCIF was carefully designed to identify outliers and unusual parameters, but every test has its limitations and alerts that are not important in a particular case may appear. Conversely, the absence of alerts does not guarantee there are no aspects of the results needing attention. It is up to the individual to critically assess their own results and, if necessary, seek expert advice.

Publication of your CIF in IUCr journals

A basic structural check has been run on your CIF. These basic checks will be run on all CIFs submitted for publication in IUCr journals (*Acta Crystallographica*, *Journal of Applied Crystallography*, *Journal of Synchrotron Radiation*); however, if you intend to submit to *Acta Crystallographica Section C* or *E* or *IUCrData*, you should make sure that full publication checks are run on the final version of your CIF prior to submission.

Publication of your CIF in other journals

Please refer to the *Notes for Authors* of the relevant journal for any special instructions relating to CIF submission.

PLATON version of 22/08/2024; check.def file version of 21/08/2024

Datablock mo_2024_fkpb03_3_0m - ellipsoid plot

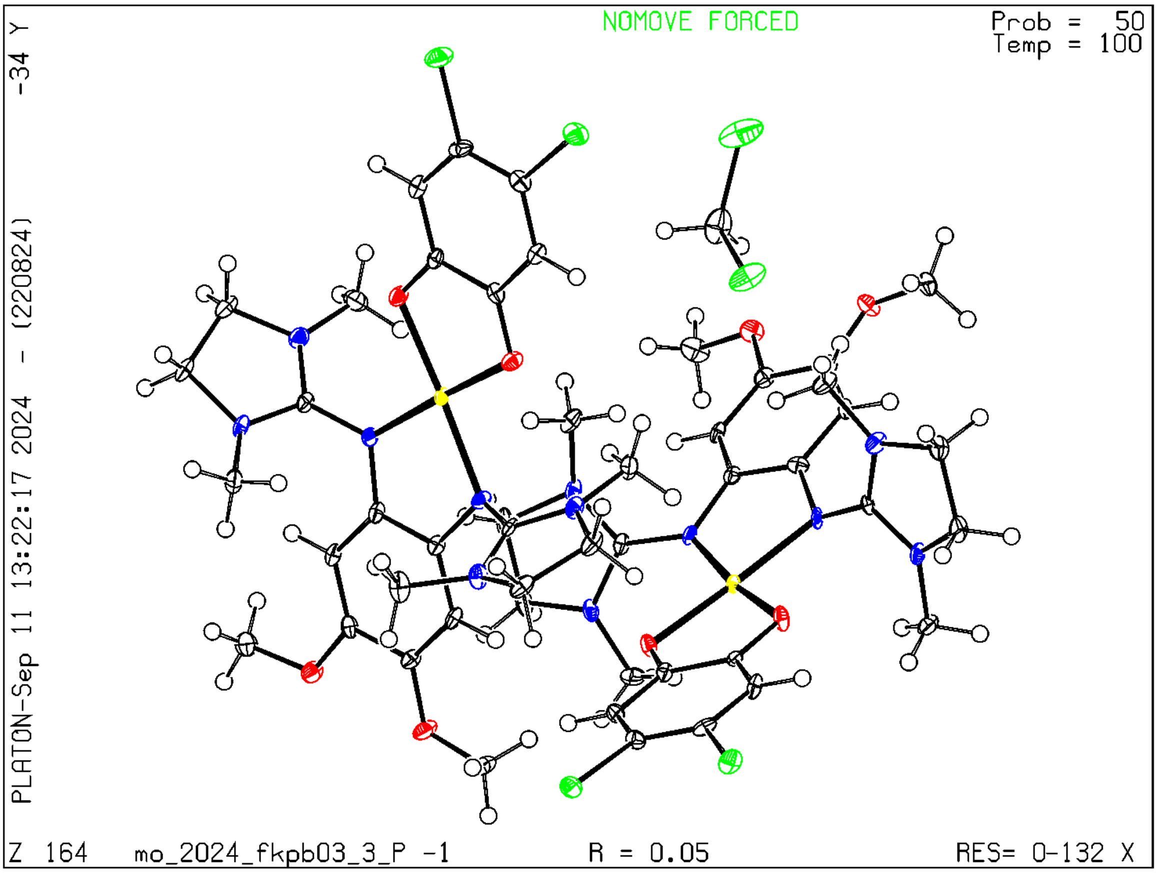

Supplement: Supplementary file 2 — Supporting Information [file CHEM-31-e03160-s002.zip › mo_2024_fkpb03_3_0m_cifreport.pdf]
