# Supplementary material for: Bistability in Palladium Complexes with Two Different Redox‐Active Ligands of Orthogonal Charge Regimes
Source: Chemistry. 2025 Nov 4;31(69):e03160. doi: 10.1002/chem.202503160 (PMC12699171; doi:10.1002/chem.202503160)

checkCIF/PLATON report

Structure factors have been supplied for datablock(s) mo_2024_fkpb19_1ma

THIS REPORT IS FOR GUIDANCE ONLY. IF USED AS PART OF A REVIEW PROCEDURE FOR PUBLICATION, IT SHOULD NOT REPLACE THE EXPERTISE OF AN EXPERIENCED CRYSTALLOGRAPHIC REFEREE.

No syntax errors found. CIF dictionary Interpreting this report

Datablock: mo_2024_fkpb19_1ma

Bond precision:	C-C = 0.0055 A		Wavelength=0.71073
Cell:	a=12.2882(14)	b=14.8310(15)	c=16.847(2)
	alpha=90	beta=100.829(4)	gamma=90
Temperature:	100 K		

	Calculated	Reported
Volume	3015.6(6)	3015.6(6)
Space group	P 21/n	P 1 21/n 1
Hall group	-P 2yn	-P 2yn
Moiety formula	C27 H28 Cl2 N6 O2 Pd [+ solvent]	C27 H28 Cl2 N6 O2 Pd, 1[CH2CL2]
Sum formula	C27 H28 Cl2 N6 O2 Pd [+ solvent]	C28 H30 Cl4 N6 O2 Pd
Mr	645.85	730.78
Dx,g cm-3	1.423	1.610
Z	4	4
Mu (mm-1)	0.826	1.007
F000	1312.0	1480.0
F000'	1309.57	
h,k,lmax	15,18,20	15,18,20
Nref	5931	5930
Tmin,Tmax	0.844,0.904	0.634,0.746
Tmin'	0.793	

Correction method= # Reported T Limits: Tmin=0.634 Tmax=0.746
AbsCorr = MULTI-SCAN

Data completeness= 1.000 Theta(max)= 25.997

R(reflections)= 0.0401(5096)

wR2(reflections)= 0.0992(5930)

S = 1.042

Npar= 349

The following ALERTS were generated. Each ALERT has the format
test-name_ALERT_alert-type_alert-level.
Click on the hyperlinks for more details of the test.

Alert level C

PLAT906_ALERT_3_C Large K Value in the Analysis of Variance ...... 2.025 Check

---

Alert level G

FORMU01_ALERT_2_G There is a discrepancy between the atom counts in the
_chemical_formula_sum and the formula from the _atom_site* data.
Atom count from _chemical_formula_sum:C28 H30 Cl4 N6 O2 Pd1
Atom count from the _atom_site data: C27 H28 Cl2 N6 O2 Pd1
CELLZ01_ALERT_1_G Difference between formula and atom_site contents detected.
CELLZ01_ALERT_1_G ALERT: Large difference may be due to a
symmetry error - see SYMMG tests
From the CIF: _cell_formula_units_Z 4
From the CIF: _chemical_formula_sum C28 H30 Cl4 N6 O2 Pd
TEST: Compare cell contents of formula and atom_site data

atom	Z*formula	cif sites	diff
C	112.00	108.00	4.00
H	120.00	112.00	8.00
Cl	16.00	8.00	8.00
N	24.00	24.00	0.00
O	8.00	8.00	0.00
Pd	4.00	4.00	0.00

PLAT041_ALERT_1_G Calc. and Reported SumFormula Strings Differ Please Check
Calc: C27 H28 Cl2 N6 O2 Pd
Rep.: C28 H30 Cl4 N6 O2 Pd
PLAT042_ALERT_1_G Calc. and Reported MoietyFormula Strings Differ Please Check
Calc: C27 H28 Cl2 N6 O2 Pd
Rep.: C27 H28 Cl2 N6 O2 Pd, 1[CH2CL2]
PLAT051_ALERT_1_G Mu(calc) and Mu(cif) Ratio Differs from 1.0 by . 17.98 %
PLAT083_ALERT_2_G SHELXL Second Parameter in WGHT Unusually Large 8.36 Why ?
PLAT232_ALERT_2_G Hirshfeld Test Diff (M-X) Pd1 --Cl2 . 5.4 s.u.
PLAT398_ALERT_2_G Deviating C-O-C Angle From 120 for O1 . 104.6 Degree
PLAT398_ALERT_2_G Deviating C-O-C Angle From 120 for O2 . 104.5 Degree
PLAT432_ALERT_2_G Short Inter X...Y Contact C12 ..C21 . 3.16 Ang.
1/2+x,1/2-y,1/2+z = 4_666 Check
PLAT605_ALERT_4_G Largest Solvent Accessible VOID in the Structure 112 A**3
PLAT794_ALERT_5_G Tentative Bond Valency for Pd1 (II) . 2.05 Info
PLAT868_ALERT_4_G ALERTS Due to the Use of _smtbx_masks Suppressed ! Info
PLAT910_ALERT_3_G Missing # of FCF Reflection(s) Below Theta(Min). 1 Note
0 1 1,
PLAT967_ALERT_5_G Note: Two-Theta Cutoff Value in Embedded .res .. 52.0 Degree
PLAT969_ALERT_5_G The 'Henn et al.' R-Factor-gap value .... 1.904 Note
Predicted wR2: Based on SigI**2 5.21 or SHELX Weight 9.52
PLAT978_ALERT_2_G Number C-C Bonds with Positive Residual Density. 2 Info

```
 0 ALERT level A = Most likely a serious problem - resolve or explain
 0 ALERT level B = A potentially serious problem, consider carefully
 1 ALERT level C = Check. Ensure it is not caused by an omission or oversight
18 ALERT level G = General information/check it is not something unexpected

 5 ALERT type 1 CIF construction/syntax error, inconsistent or missing data
 7 ALERT type 2 Indicator that the structure model may be wrong or deficient
 2 ALERT type 3 Indicator that the structure quality may be low
 2 ALERT type 4 Improvement, methodology, query or suggestion
 3 ALERT type 5 Informative message, check
```

It is advisable to attempt to resolve as many as possible of the alerts in all categories. Often the minor alerts point to easily fixed oversights, errors and omissions in your CIF or refinement strategy, so attention to these fine details can be worthwhile. In order to resolve some of the more serious problems it may be necessary to carry out additional measurements or structure refinements. However, the purpose of your study may justify the reported deviations and the more serious of these should normally be commented upon in the discussion or experimental section of a paper or in the "special_details" fields of the CIF. checkCIF was carefully designed to identify outliers and unusual parameters, but every test has its limitations and alerts that are not important in a particular case may appear. Conversely, the absence of alerts does not guarantee there are no aspects of the results needing attention. It is up to the individual to critically assess their own results and, if necessary, seek expert advice.

Publication of your CIF in IUCr journals

A basic structural check has been run on your CIF. These basic checks will be run on all CIFs submitted for publication in IUCr journals (*Acta Crystallographica*, *Journal of Applied Crystallography*, *Journal of Synchrotron Radiation*); however, if you intend to submit to *Acta Crystallographica Section C* or *E* or *IUCrData*, you should make sure that full publication checks are run on the final version of your CIF prior to submission.

Publication of your CIF in other journals

Please refer to the *Notes for Authors* of the relevant journal for any special instructions relating to CIF submission.

PLATON version of 11/11/2024; check.def file version of 11/11/2024

Datablock mo_2024_fkpb19_1ma - ellipsoid plot

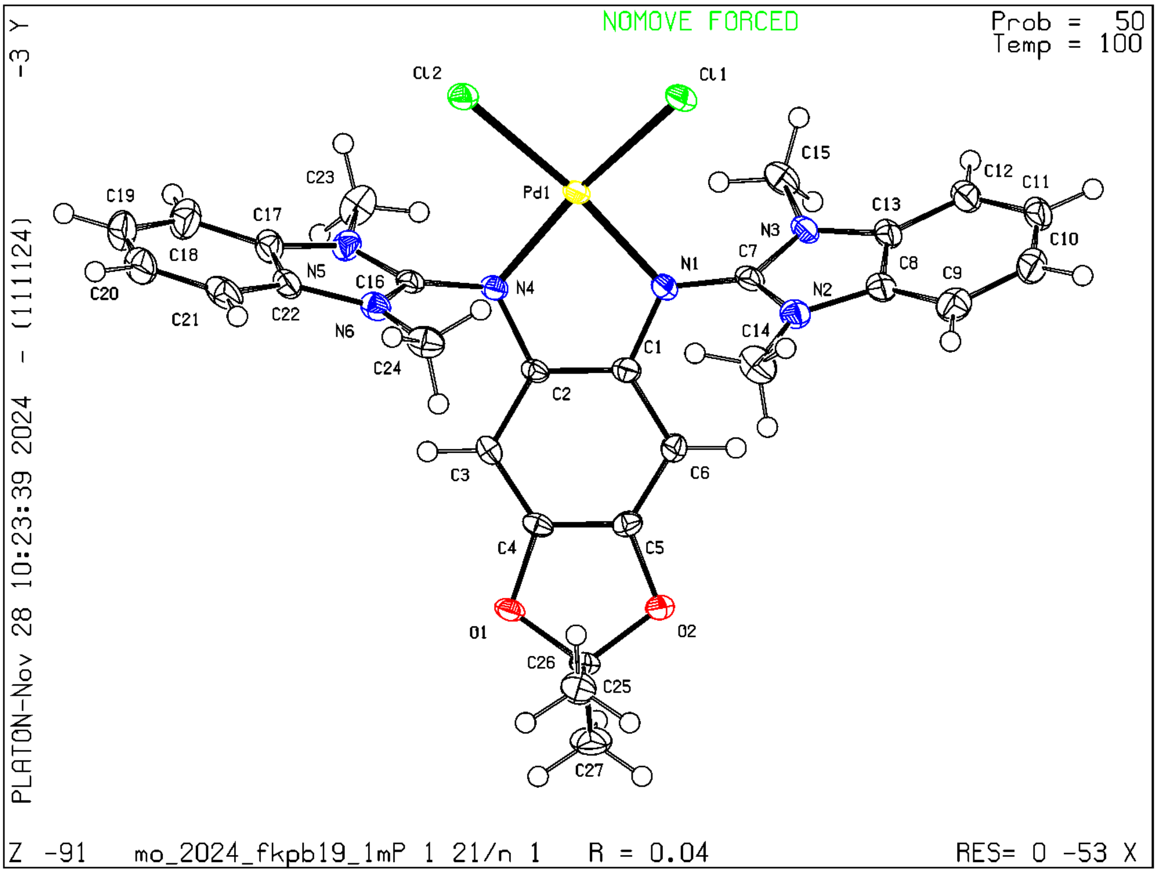

Supplement: Supplementary file 2 — Supporting Information [file CHEM-31-e03160-s002.zip › mo_2024_fkpb19_1ma_cifreport.pdf]
