# Supplementary material for: Bistability in Palladium Complexes with Two Different Redox‐Active Ligands of Orthogonal Charge Regimes
Source: Chemistry. 2025 Nov 4;31(69):e03160. doi: 10.1002/chem.202503160 (PMC12699171; doi:10.1002/chem.202503160)

checkCIF/PLATON report

Structure factors have been supplied for datablock(s) mo_2025_fk107_3b_0ma

THIS REPORT IS FOR GUIDANCE ONLY. IF USED AS PART OF A REVIEW PROCEDURE FOR PUBLICATION, IT SHOULD NOT REPLACE THE EXPERTISE OF AN EXPERIENCED CRYSTALLOGRAPHIC REFEREE.

No syntax errors found. CIF dictionary Interpreting this report

Datablock: mo_2025_fk107_3b_0ma

Bond precision: C-C = 0.0062 A Wavelength=0.71073

Cell:	a=9.9296(7)	b=17.7180(12)	c=27.3097(18)
	alpha=90	beta=94.490(2)	gamma=90
Temperature:	100 K		

	Calculated	Reported
Volume	4789.9(6)	4789.9(6)
Space group	P 21/n	P 1 21/n 1
Hall group	-P 2yn	-P 2yn
Moiety formula	C33 H28 Cl4 N6 O4 Pd, 1.5(C6 H4 F2), F6 P	C33 H28 Cl4 N6 O4 Pd, F6 P, 1.5(C6 H4 F2)
Sum formula	C42 H34 Cl4 F9 N6 O4 P Pd	C42 H34 Cl4 F9 N6 O4 P Pd
Mr	1136.92	1136.96
Dx,g cm-3	1.577	1.577
Z	4	4
Mu (mm-1)	0.726	0.726
F000	2280.0	2280.5
F000'	2279.99	
h,k,lmax	12,21,33	12,21,33
Nref	9406	9401
Tmin,Tmax	0.909,0.944	0.628,0.745
Tmin'	0.721	

Correction method= # Reported T Limits: Tmin=0.628 Tmax=0.745
AbsCorr = NONE

Data completeness= 0.999 Theta(max)= 26.000

R(reflections)= 0.0536(7607) wR2(reflections)= 0.1523(9401)

S = 1.083 Npar= 610

The following ALERTS were generated. Each ALERT has the format
test-name_ALERT_alert-type_alert-level.
Click on the hyperlinks for more details of the test.

Alert level B

PLAT213_ALERT_2_B Atom C11 has ADP max/min Ratio ..... 4.6 oblate

Alert level C

PLAT042_ALERT_1_C Calc. and Reported MoietyFormula Strings Differ Please Check
Calc: C33 H28 Cl4 N6 O4 Pd, 1.5(C6 H4 F2), F6 P
Rep.: C33 H28 Cl4 N6 O4 Pd, F6 P, 1.5(C6 H4 F2)
PLAT094_ALERT_2_C Ratio of Maximum / Minimum Residual Density .... 2.14 Report
PLAT250_ALERT_2_C Large U3/U1 Ratio for <U(i,j)> Tensor(Resd 1) 2.2 Note
PLAT411_ALERT_2_C Short Inter H...H Contact H10 ..H19 . 2.14 Ang.
x,-1+y,z = 1_545 Check
PLAT906_ALERT_3_C Large K Value in the Analysis of Variance ...... 2.186 Check
PLAT911_ALERT_3_C Missing FCF Refl Between Thmin & STh/L= 0.600 2 Report
-1 0 1, 2 0 2,
PLAT971_ALERT_2_C Check Calcd Resid. Dens. 0.64Ang From C11 1.70 eA-3

Alert level G

PLAT003_ALERT_2_G Number of Uiso or U(i,j) Restrained non-H-Atoms 18 Report
PLAT068_ALERT_1_G Reported F000 Differs from Calcd (or Missing)... Please Check
PLAT073_ALERT_1_G H-atoms ref., but hydrogen treatment Reported as constr Check
PLAT083_ALERT_2_G SHELXL Second Parameter in WGHT Unusually Large 27.05 Why ?
PLAT178_ALERT_4_G The CIF-Embedded .res File Contains SIMU Records 3 Report
PLAT187_ALERT_4_G The CIF-Embedded .res File Contains RIGU Records 3 Report
PLAT188_ALERT_3_G A Non-default SIMU Restraint Value has been used 0.0200 Report
PLAT190_ALERT_3_G A Non-default RIGU Restraint Value for First Par 0.0020 Report
PLAT190_ALERT_3_G A Non-default RIGU Restraint Value for SecondPar 0.0020 Report
PLAT300_ALERT_4_G Atom Site Occupancy of F7 Constrained at 0.75 Check
PLAT300_ALERT_4_G Atom Site Occupancy of F8 Constrained at 0.75 Check
PLAT300_ALERT_4_G Atom Site Occupancy of C34 Constrained at 0.75 Check
PLAT300_ALERT_4_G Atom Site Occupancy of C35 Constrained at 0.75 Check
PLAT300_ALERT_4_G Atom Site Occupancy of C36 Constrained at 0.75 Check
PLAT300_ALERT_4_G Atom Site Occupancy of C37 Constrained at 0.75 Check
PLAT300_ALERT_4_G Atom Site Occupancy of C38 Constrained at 0.75 Check
PLAT300_ALERT_4_G Atom Site Occupancy of C39 Constrained at 0.75 Check
PLAT300_ALERT_4_G Atom Site Occupancy of H36 Constrained at 0.75 Check
PLAT300_ALERT_4_G Atom Site Occupancy of H37 Constrained at 0.75 Check
PLAT300_ALERT_4_G Atom Site Occupancy of H38 Constrained at 0.75 Check
PLAT300_ALERT_4_G Atom Site Occupancy of H39 Constrained at 0.75 Check
PLAT300_ALERT_4_G Atom Site Occupancy of F9 Constrained at 0.75 Check
PLAT300_ALERT_4_G Atom Site Occupancy of F10 Constrained at 0.75 Check
PLAT300_ALERT_4_G Atom Site Occupancy of C40 Constrained at 0.75 Check
PLAT300_ALERT_4_G Atom Site Occupancy of C41 Constrained at 0.75 Check
PLAT300_ALERT_4_G Atom Site Occupancy of C42 Constrained at 0.75 Check
PLAT300_ALERT_4_G Atom Site Occupancy of C43 Constrained at 0.75 Check
PLAT300_ALERT_4_G Atom Site Occupancy of C44 Constrained at 0.75 Check
PLAT300_ALERT_4_G Atom Site Occupancy of C45 Constrained at 0.75 Check
PLAT300_ALERT_4_G Atom Site Occupancy of H42 Constrained at 0.75 Check
PLAT300_ALERT_4_G Atom Site Occupancy of H43 Constrained at 0.75 Check

```
PLAT300_ALERT_4_G Atom Site Occupancy of H44        Constrained at       0.75 Check
PLAT300_ALERT_4_G Atom Site Occupancy of H45        Constrained at       0.75 Check
PLAT302_ALERT_4_G Anion/Solvent/Minor-Residue Disorder (Resd    2)       100% Note
PLAT302_ALERT_4_G Anion/Solvent/Minor-Residue Disorder (Resd    3)       100% Note
PLAT398_ALERT_2_G Deviating  C-O-C   Angle From 120 for O1       .      108.0 Degree
PLAT398_ALERT_2_G Deviating  C-O-C   Angle From 120 for O2       .      108.0 Degree
PLAT432_ALERT_2_G Short Inter X...Y Contact  F5       ..C14      .       2.93 Ang.
                                                     x,y,z  =      1_555 Check
PLAT432_ALERT_2_G Short Inter X...Y Contact  F5       ..C15      .       2.96 Ang.
                                                   1+x,y,z  =      1_655 Check
PLAT769_ALERT_4_G CIF Embedded Explicitly Supplied Scattering Data     Please Note
PLAT794_ALERT_5_G Tentative Bond Valency for Pd1       (II)      .       2.34 Info
PLAT860_ALERT_3_G Number of Least-Squares Restraints ............        216 Note
PLAT883_ALERT_1_G Absent Datum for _atom_sites_solution_primary ..     Please Do !
PLAT910_ALERT_3_G Missing # of FCF Reflection(s) Below Theta(Min).          2 Note
                0  1  1,   0  0  2,
PLAT913_ALERT_3_G Missing # of Very Strong Reflections in FCF ....          1 Note
                2  0  2,
PLAT933_ALERT_2_G Number of HKL-OMIT Records in Embedded .res File          1 Note
               -1  0  1,
PLAT967_ALERT_5_G Note: Two-Theta Cutoff Value in Embedded .res ..       52.0 Degree
PLAT969_ALERT_5_G The 'Henn et al.' R-Factor-gap value ..........      4.916 Note
              Predicted wR2: Based on SigI**2  3.10 or SHELX Weight 14.06
PLAT978_ALERT_2_G Number C-C Bonds with Positive Residual Density.          4 Info
PLAT982_ALERT_1_G The Pd-f'=   -0.9775 Deviates from  IT-Value =     -0.9988 Check
PLAT983_ALERT_1_G The Cl-f"=    0.1597 Deviates from  IT-Value =      0.1585 Check
PLAT983_ALERT_1_G The Pd-f"=    1.0117 Deviates from  IT-Value =      1.0072 Check
```

---

 0 **ALERT level A** = Most likely a serious problem - resolve or explain
 1 **ALERT level B** = A potentially serious problem, consider carefully
 7 **ALERT level C** = Check. Ensure it is not caused by an omission or oversight
52 **ALERT level G** = General information/check it is not something unexpected

 7 ALERT type 1 CIF construction/syntax error, inconsistent or missing data
13 ALERT type 2 Indicator that the structure model may be wrong or deficient
 8 ALERT type 3 Indicator that the structure quality may be low
29 ALERT type 4 Improvement, methodology, query or suggestion
 3 ALERT type 5 Informative message, check

It is advisable to attempt to resolve as many as possible of the alerts in all categories. Often the minor alerts point to easily fixed oversights, errors and omissions in your CIF or refinement strategy, so attention to these fine details can be worthwhile. In order to resolve some of the more serious problems it may be necessary to carry out additional measurements or structure refinements. However, the purpose of your study may justify the reported deviations and the more serious of these should normally be commented upon in the discussion or experimental section of a paper or in the "special_details" fields of the CIF. checkCIF was carefully designed to identify outliers and unusual parameters, but every test has its limitations and alerts that are not important in a particular case may appear. Conversely, the absence of alerts does not guarantee there are no aspects of the results needing attention. It is up to the individual to critically assess their own results and, if necessary, seek expert advice.

Publication of your CIF in IUCr journals

A basic structural check has been run on your CIF. These basic checks will be run on all CIFs submitted for publication in IUCr journals (*Acta Crystallographica*, *Journal of Applied Crystallography*, *Journal of Synchrotron Radiation*); however, if you intend to submit to *Acta Crystallographica Section C* or *E* or *IUCrData*, you should make sure that full publication checks are run on the final version of your CIF prior to submission.

Publication of your CIF in other journals

Please refer to the *Notes for Authors* of the relevant journal for any special instructions relating to CIF submission.

PLATON version of 02/02/2025; check.def file version of 02/02/2025

Datablock mo_2025_fk107_3b_0ma - ellipsoid plot

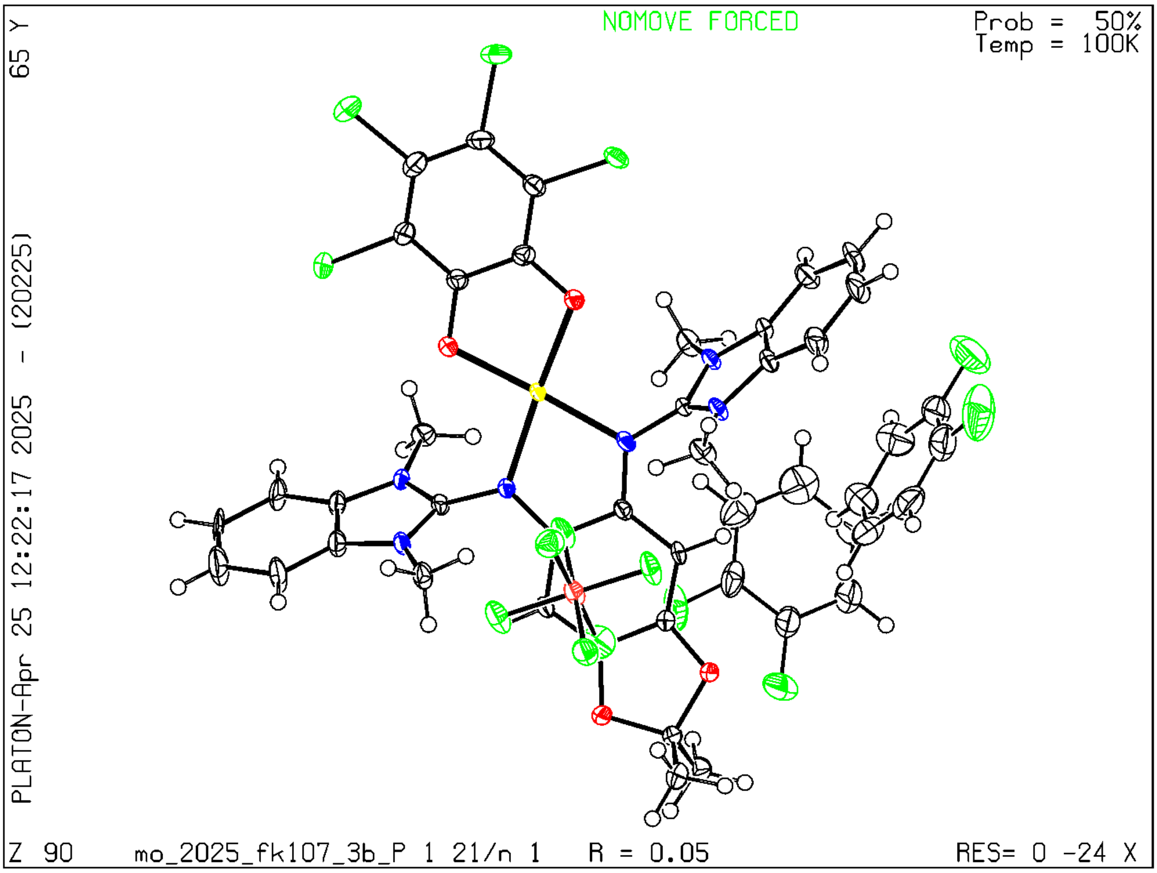

Supplement: Supplementary file 2 — Supporting Information [file CHEM-31-e03160-s002.zip › mo_2025_fk107_3b_0ma_cifreport.pdf]
