# Supplementary material for: Bistability in Palladium Complexes with Two Different Redox‐Active Ligands of Orthogonal Charge Regimes
Source: Chemistry. 2025 Nov 4;31(69):e03160. doi: 10.1002/chem.202503160 (PMC12699171; doi:10.1002/chem.202503160)

checkCIF/PLATON report

Structure factors have been supplied for datablock(s) mo_2025_fk111_3m

THIS REPORT IS FOR GUIDANCE ONLY. IF USED AS PART OF A REVIEW PROCEDURE FOR PUBLICATION, IT SHOULD NOT REPLACE THE EXPERTISE OF AN EXPERIENCED CRYSTALLOGRAPHIC REFEREE.

No syntax errors found. CIF dictionary Interpreting this report

Datablock: mo_2025_fk111_3m

```
Bond precision:   C-C = 0.0128 A           Wavelength=0.71073

Cell:              a=18.781(2)     b=12.9338(15)      c=24.859(3)
                   alpha=90        beta=90            gamma=90
Temperature:       100 K

                Calculated                    Reported
Volume          6038.5(12)                    6038.6(12)
Space group     P b c a                       Pbca
Hall group      -P 2ac 2ab                    -P 2ac 2ab
Moiety formula  C24 H30 Cl2 N6 O4 Pd, F6 P    C24 H30 Cl2 N6 O4 Pd, F6 P
Sum formula     C24 H30 Cl2 F6 N6 O4 P Pd     C24 H30 Cl2 F6 N6 O4 P Pd
Mr              788.81                        788.81
Dx,g cm-3       1.735                         1.735
Z               8                             8
Mu (mm-1)       0.924                         0.924
F000            3176.0                        3176.0
F000'           3172.85
h,k,lmax        23,16,30                      23,16,30
Nref            6039                          6003
Tmin,Tmax       0.925,0.964                   0.618,0.745
Tmin'           0.879

Correction method= # Reported T Limits: Tmin=0.618 Tmax=0.745
AbsCorr = MULTI-SCAN

Data completeness= 0.994           Theta(max)= 26.176

                                                           wR2(reflections)=
R(reflections)= 0.0706( 3224)                              0.2231( 6003)

S = 1.009                    Npar= 458
```

The following ALERTS were generated. Each ALERT has the format
test-name_ALERT_alert-type_alert-level.
Click on the hyperlinks for more details of the test.

Alert level B

```
RINTA01_ALERT_3_B  The value of Rint is greater than 0.18
            Rint given   0.220
```

Author Response: Due to very small and poorly diffracting crystal.

```
PLAT020_ALERT_3_B The Value of Rint is Greater Than 0.12 .........      0.220 Report
```

Author Response: Due to very small and poorly diffracting crystal.

Alert level C

```
PLAT230_ALERT_2_C Hirshfeld Test Diff for    O4       --C20      .        5.6 s.u.
PLAT342_ALERT_3_C Low Bond Precision on  C-C Bonds ...............    0.01279 Ang.
PLAT905_ALERT_3_C Negative K value in the Analysis of Variance ...     -3.264 Report
PLAT975_ALERT_2_C Check Calcd Resid. Dens.  1.00Ang From O4      .       0.49 eA-3
PLAT975_ALERT_2_C Check Calcd Resid. Dens.  0.79Ang From O3      .       0.43 eA-3
```

Alert level G

```
PLAT002_ALERT_2_G Number of Distance or Angle Restraints on AtSite         13 Note
PLAT003_ALERT_2_G Number of Uiso or U(i,j)  Restrained non-H-Atoms         13 Report
PLAT083_ALERT_2_G SHELXL Second Parameter in WGHT  Unusually Large      29.66 Why ?
PLAT172_ALERT_4_G The CIF-Embedded .res File Contains DFIX Records          2 Report
PLAT176_ALERT_4_G The CIF-Embedded .res File Contains SADI Records          6 Report
PLAT178_ALERT_4_G The CIF-Embedded .res File Contains SIMU Records          2 Report
PLAT187_ALERT_4_G The CIF-Embedded .res File Contains RIGU Records          2 Report
PLAT191_ALERT_3_G A Non-default SADI Restraint Value has been used     0.0400 Report
PLAT191_ALERT_3_G A Non-default SADI Restraint Value has been used     0.0400 Report
PLAT191_ALERT_3_G A Non-default SADI Restraint Value has been used     0.0500 Report
PLAT191_ALERT_3_G A Non-default SADI Restraint Value has been used     0.0500 Report
PLAT244_ALERT_4_G Low    'Solvent' Ueq as Compared to Neighbors of         P1 Check
PLAT302_ALERT_4_G Anion/Solvent/Minor-Residue Disorder (Resd    2)        86% Note
PLAT432_ALERT_2_G Short Inter X...Y Contact  C15      ..C24      .       3.11 Ang.
                                         -1/2+x,y,1/2-z  =      6_556 Check
PLAT794_ALERT_5_G Tentative Bond Valency for Pd1       (II)      .       2.25 Info
PLAT860_ALERT_3_G Number of Least-Squares Restraints .............        302 Note
PLAT910_ALERT_3_G Missing # of FCF Reflection(s) Below Theta(Min).          1 Note
               0   0   2,
PLAT912_ALERT_4_G Missing # of FCF Reflections Above STh/L=  0.600         35 Note
PLAT969_ALERT_5_G The 'Henn et al.' R-Factor-gap value ...........      1.944 Note
              Predicted wR2: Based on SigI**2 11.47 or SHELX Weight 22.10
PLAT978_ALERT_2_G Number C-C Bonds with Positive Residual Density.          0 Info
```

0 **ALERT level A** = Most likely a serious problem - resolve or explain
2 **ALERT level B** = A potentially serious problem, consider carefully

```
 5 ALERT level C = Check. Ensure it is not caused by an omission or oversight
20 ALERT level G = General information/check it is not something unexpected

 0 ALERT type 1 CIF construction/syntax error, inconsistent or missing data
 8 ALERT type 2 Indicator that the structure model may be wrong or deficient
10 ALERT type 3 Indicator that the structure quality may be low
 7 ALERT type 4 Improvement, methodology, query or suggestion
 2 ALERT type 5 Informative message, check
```

It is advisable to attempt to resolve as many as possible of the alerts in all categories. Often the minor alerts point to easily fixed oversights, errors and omissions in your CIF or refinement strategy, so attention to these fine details can be worthwhile. In order to resolve some of the more serious problems it may be necessary to carry out additional measurements or structure refinements. However, the purpose of your study may justify the reported deviations and the more serious of these should normally be commented upon in the discussion or experimental section of a paper or in the "special_details" fields of the CIF. checkCIF was carefully designed to identify outliers and unusual parameters, but every test has its limitations and alerts that are not important in a particular case may appear. Conversely, the absence of alerts does not guarantee there are no aspects of the results needing attention. It is up to the individual to critically assess their own results and, if necessary, seek expert advice.

Publication of your CIF in IUCr journals

A basic structural check has been run on your CIF. These basic checks will be run on all CIFs submitted for publication in IUCr journals (*Acta Crystallographica*, *Journal of Applied Crystallography*, *Journal of Synchrotron Radiation*); however, if you intend to submit to *Acta Crystallographica Section C* or *E* or *IUCrData*, you should make sure that full publication checks are run on the final version of your CIF prior to submission.

Publication of your CIF in other journals

Please refer to the *Notes for Authors* of the relevant journal for any special instructions relating to CIF submission.

PLATON version of 02/02/2025; check.def file version of 02/02/2025

Datablock mo_2025_fk111_3m - ellipsoid plot

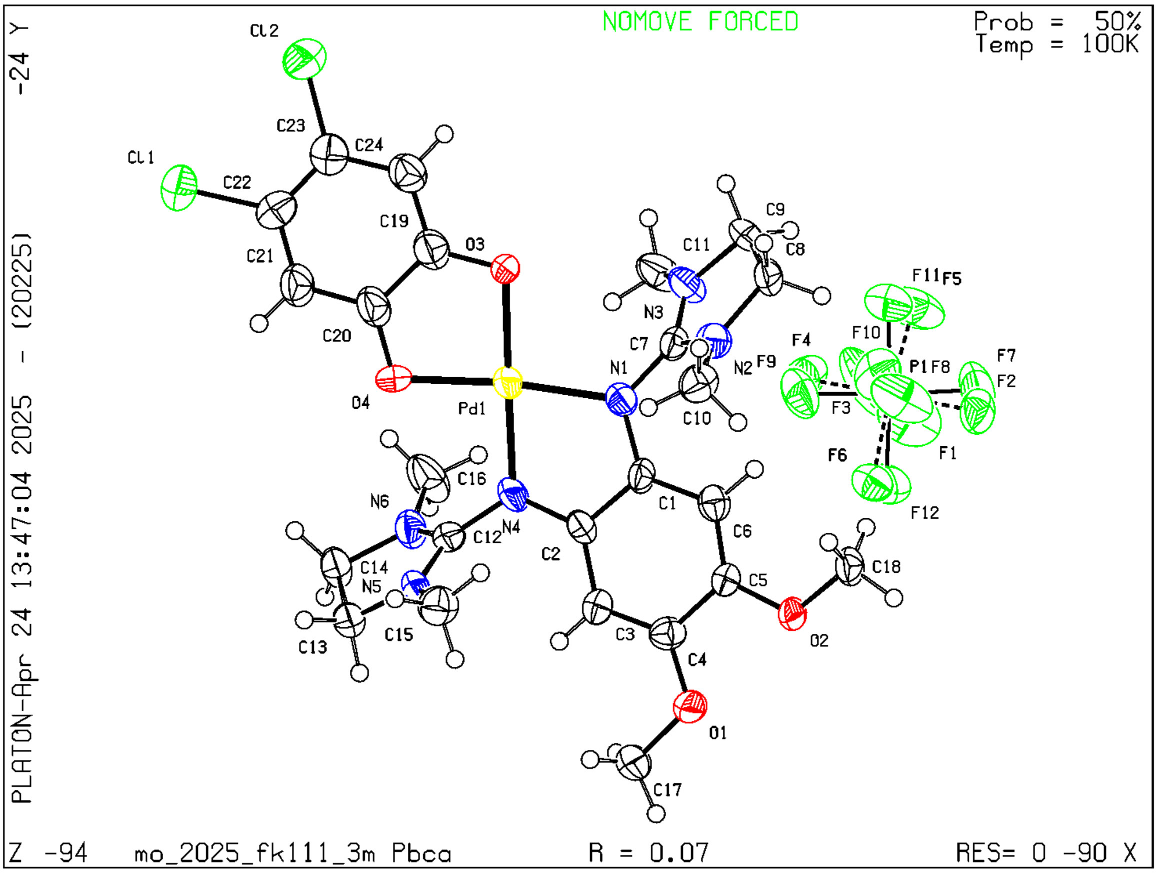

Supplement: Supplementary file 2 — Supporting Information [file CHEM-31-e03160-s002.zip › mo_2025_fk111_3m_cifreport.pdf]
