# Supplementary material for: Bistability in Palladium Complexes with Two Different Redox‐Active Ligands of Orthogonal Charge Regimes
Source: Chemistry. 2025 Nov 4;31(69):e03160. doi: 10.1002/chem.202503160 (PMC12699171; doi:10.1002/chem.202503160)

checkCIF/PLATON report

Structure factors have been supplied for datablock(s) mo_2025_fk116_0m

THIS REPORT IS FOR GUIDANCE ONLY. IF USED AS PART OF A REVIEW PROCEDURE FOR PUBLICATION, IT SHOULD NOT REPLACE THE EXPERTISE OF AN EXPERIENCED CRYSTALLOGRAPHIC REFEREE.

No syntax errors found. CIF dictionary Interpreting this report

Datablock: mo_2025_fk116_0m

Bond precision:	C-C = 0.0029 A		Wavelength=0.71073
Cell:	a=11.2891(5)	b=11.5983(5)	c=21.9259(9)
	alpha=90	beta=94.216(2)	gamma=90
Temperature:	100 K		

	Calculated	Reported
Volume	2863.1(2)	2863.1(2)
Space group	P 21/n	P 1 21/n 1
Hall group	-P 2yn	-P 2yn
Moiety formula	C24 H28 Cl4 N6 O4 Pd	C24 H28 Cl4 N6 O4 Pd
Sum formula	C24 H28 Cl4 N6 O4 Pd	C24 H28 Cl4 N6 O4 Pd
Mr	712.72	712.72
Dx,g cm-3	1.653	1.653
Z	4	4
Mu (mm-1)	1.064	1.064
F000	1440.0	1440.0
F000'	1438.82	
h,k,lmax	14,15,28	14,15,28
Nref	6565	6551
Tmin,Tmax	0.805,0.918	0.663,0.746
Tmin'	0.682	

Correction method= # Reported T Limits: Tmin=0.663 Tmax=0.746
AbsCorr = MULTI-SCAN

Data completeness= 0.998 Theta(max)= 27.500

R(reflections)= 0.0250(6117) wR2(reflections)= 0.0584(6551)

S = 1.064 Npar= 358

The following ALERTS were generated. Each ALERT has the format
test-name_ALERT_alert-type_alert-level.
Click on the hyperlinks for more details of the test.

Alert level C

```
PLAT431_ALERT_2_C Short Inter HL..A Contact  Cl2      ..N3       .        3.17 Ang.
                                                 1+x,y,z  =      1_655 Check
PLAT911_ALERT_3_C Missing FCF Refl Between Thmin & STh/L=    0.600          13 Report
                3  0  1,   1  1  1,   0  2  1,   1  2  1,   1  3  1,  -2  0  2,
                4  0  2,   1  3  2,  -3  1  3,   2  0  4,  -1  0  5,   0  2  5,
               -2  0 10,
PLAT913_ALERT_3_C Missing # of Very Strong Reflections in FCF ....          12 Note
                3  0  1,   0  2  1,   1  2  1,   1  3  1,  -2  0  2,   0  0  2,
                4  0  2,   1  3  2,  -3  1  3,  -1  0  5,   0  2  5,  -2  0 10,
```

Alert level G

```
PLAT380_ALERT_4_G Incorrectly? Oriented X(sp2)-Methyl Moiety .....         C16 Check
PLAT794_ALERT_5_G Tentative Bond Valency for Pd1       (II)       .       2.30 Info
PLAT910_ALERT_3_G Missing FCF Reflection(s) Below Theta(Min)[Deg]=       1.97 Note
                0  0  2,
PLAT967_ALERT_5_G Note: Two-Theta Cutoff Value in Embedded .res ..       55.0 Degree
PLAT969_ALERT_5_G The 'Henn et al.' R-Factor-gap value ..........      2.199 Note
              Predicted wR2: Based on SigI**2  2.65 or SHELX Weight  5.49
PLAT978_ALERT_2_G Number C-C Bonds with Positive Residual Density.           7 Info
```

0 **ALERT level A** = Most likely a serious problem - resolve or explain
0 **ALERT level B** = A potentially serious problem, consider carefully
3 **ALERT level C** = Check. Ensure it is not caused by an omission or oversight
6 **ALERT level G** = General information/check it is not something unexpected

0 ALERT type 1 CIF construction/syntax error, inconsistent or missing data
2 ALERT type 2 Indicator that the structure model may be wrong or deficient
3 ALERT type 3 Indicator that the structure quality may be low
1 ALERT type 4 Improvement, methodology, query or suggestion
3 ALERT type 5 Informative message, check

It is advisable to attempt to resolve as many as possible of the alerts in all categories. Often the minor alerts point to easily fixed oversights, errors and omissions in your CIF or refinement strategy, so attention to these fine details can be worthwhile. In order to resolve some of the more serious problems it may be necessary to carry out additional measurements or structure refinements. However, the purpose of your study may justify the reported deviations and the more serious of these should normally be commented upon in the discussion or experimental section of a paper or in the "special_details" fields of the CIF. checkCIF was carefully designed to identify outliers and unusual parameters, but every test has its limitations and alerts that are not important in a particular case may appear. Conversely, the absence of alerts does not guarantee there are no aspects of the results needing attention. It is up to the individual to critically assess their own results and, if necessary, seek expert advice.

Publication of your CIF in IUCr journals

A basic structural check has been run on your CIF. These basic checks will be run on all CIFs submitted for publication in IUCr journals (*Acta Crystallographica*, *Journal of Applied Crystallography*, *Journal of Synchrotron Radiation*); however, if you intend to submit to *Acta Crystallographica Section C* or *E* or *IUCrData*, you should make sure that full publication checks are run on the final version of your CIF prior to submission.

Publication of your CIF in other journals

Please refer to the *Notes for Authors* of the relevant journal for any special instructions relating to CIF submission.

PLATON version of 04/06/2025; check.def file version of 30/05/2025

Datablock mo_2025_fk116_0m - ellipsoid plot

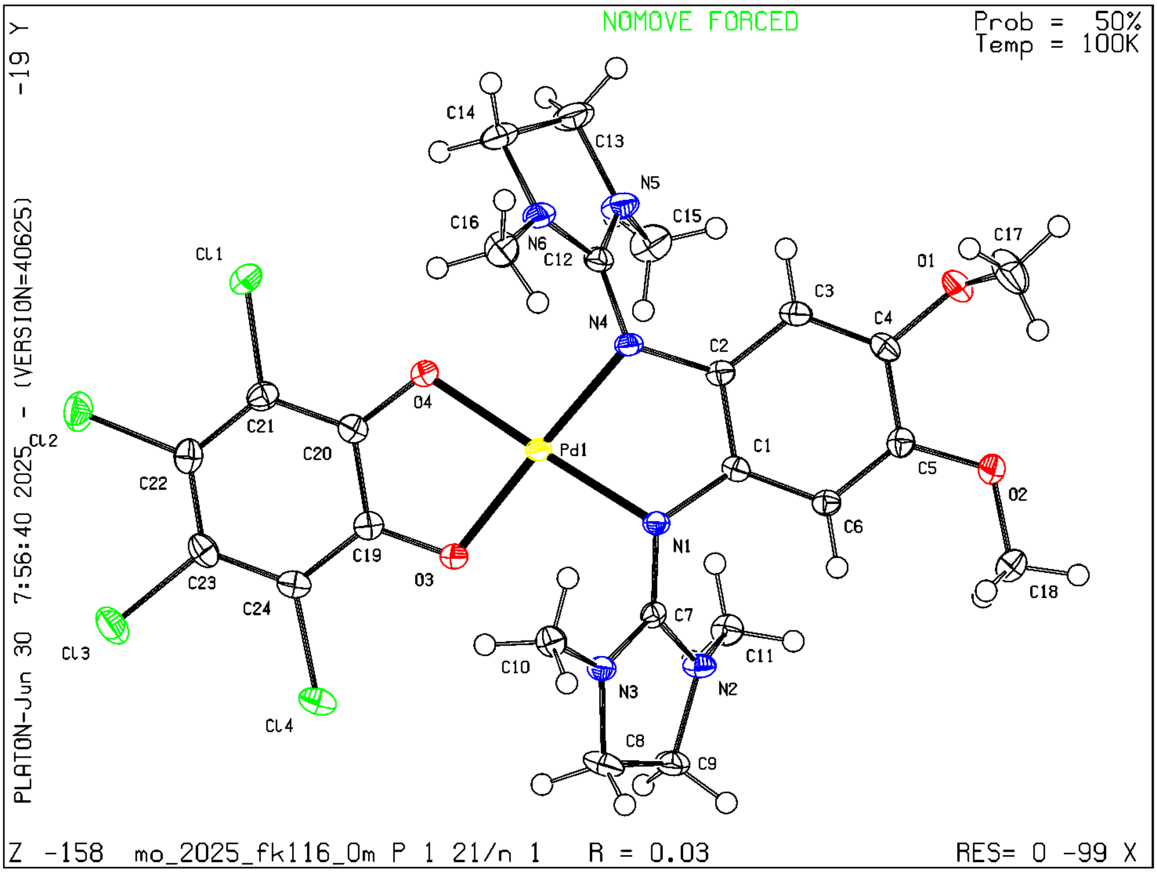

Supplement: Supplementary file 2 — Supporting Information [file CHEM-31-e03160-s002.zip › mo_2025_fk116_0m_cifreport.pdf]
