# Supplementary material for: Bistability in Palladium Complexes with Two Different Redox‐Active Ligands of Orthogonal Charge Regimes
Source: Chemistry. 2025 Nov 4;31(69):e03160. doi: 10.1002/chem.202503160 (PMC12699171; doi:10.1002/chem.202503160)

checkCIF/PLATON report

Structure factors have been supplied for datablock(s) mo_2025_fk118_4_0m

THIS REPORT IS FOR GUIDANCE ONLY. IF USED AS PART OF A REVIEW PROCEDURE FOR PUBLICATION, IT SHOULD NOT REPLACE THE EXPERTISE OF AN EXPERIENCED CRYSTALLOGRAPHIC REFEREE.

No syntax errors found.   CIF dictionary   Interpreting this report

Datablock: mo_2025_fk118_4_0m

Bond precision: C-C = 0.0034 A   Wavelength=0.71073

Cell:	a=13.2611(10)	b=13.9853(10)	c=15.1673(11)
	alpha=72.399(3)	beta=79.673(3)	gamma=88.308(3)
Temperature:	100 K		

	Calculated	Reported
Volume	2636.9(3)	2636.9(3)
Space group	P -1	P-1
Hall group	-P 1	-P 1
Moiety formula	C18 H28 Cl2 N6 O2 Pd, F6 P	C18 H28 Cl2 N6 O2 Pd, F6 P
Sum formula	C18 H28 Cl2 F6 N6 O2 P Pd	C18 H28 Cl2 F6 N6 O2 P Pd
Mr	682.73	682.73
Dx,g cm-3	1.720	1.720
Z	4	4
Mu (mm-1)	1.038	1.038
F000	1372.0	1372.0
F000'	1370.28	
h,k,lmax	16,17,19	16,17,19
Nref	11497	11492
Tmin,Tmax	0.804,0.901	0.668,0.746
Tmin'	0.804	

Correction method= # Reported T Limits: Tmin=0.668 Tmax=0.746
AbsCorr = MULTI-SCAN

Data completeness= 1.000   Theta(max)= 27.000

R(reflections)= 0.0269(10346)   wR2(reflections)= 0.0699(11492)

S = 1.095   Npar= 733

The following ALERTS were generated. Each ALERT has the format
test-name_ALERT_alert-type_alert-level.
Click on the hyperlinks for more details of the test.

Alert level C

```
PLAT094_ALERT_2_C Ratio of Maximum / Minimum Residual Density ....       2.76 Report
PLAT250_ALERT_2_C Large U3/U1 Ratio for <U(i,j)> Tensor(Resd    5)        2.2 Note
PLAT977_ALERT_2_C Check Negative Difference Density on H36A      .      -0.35 eA-3
```

Alert level G

```
PLAT002_ALERT_2_G Number of Distance or Angle Restraints on AtSite         14 Note
PLAT003_ALERT_2_G Number of Uiso or U(i,j)  Restrained non-H-Atoms         16 Report
PLAT154_ALERT_1_G The s.u.'s on the Cell Angles are Equal ..(Note)      0.003 Degree
PLAT176_ALERT_4_G The CIF-Embedded .res File Contains SADI Records          6 Report
PLAT178_ALERT_4_G The CIF-Embedded .res File Contains SIMU Records          3 Report
PLAT187_ALERT_4_G The CIF-Embedded .res File Contains RIGU Records          3 Report
PLAT191_ALERT_3_G A Non-default SADI Restraint Value has been used     0.0400 Report
PLAT191_ALERT_3_G A Non-default SADI Restraint Value has been used     0.0500 Report
PLAT191_ALERT_3_G A Non-default SADI Restraint Value has been used     0.0400 Report
PLAT191_ALERT_3_G A Non-default SADI Restraint Value has been used     0.0500 Report
PLAT232_ALERT_2_G Hirshfeld Test Diff (M-X)  Pd1      --Cl1      .        6.0 s.u.
PLAT232_ALERT_2_G Hirshfeld Test Diff (M-X)  Pd2      --Cl3      .        7.3 s.u.
PLAT244_ALERT_4_G Low    'Solvent' Ueq as Compared to Neighbors of         P1 Check
PLAT300_ALERT_4_G Atom Site Occupancy of Cl2         Constrained at       0.9 Check
PLAT300_ALERT_4_G Atom Site Occupancy of Cl2B        Constrained at       0.1 Check
PLAT300_ALERT_4_G Atom Site Occupancy of P2          Constrained at       0.9 Check
PLAT300_ALERT_4_G Atom Site Occupancy of F7          Constrained at       0.9 Check
PLAT300_ALERT_4_G Atom Site Occupancy of F8          Constrained at       0.9 Check
PLAT300_ALERT_4_G Atom Site Occupancy of F9          Constrained at       0.9 Check
PLAT300_ALERT_4_G Atom Site Occupancy of F10         Constrained at       0.9 Check
PLAT300_ALERT_4_G Atom Site Occupancy of F11         Constrained at       0.9 Check
PLAT300_ALERT_4_G Atom Site Occupancy of F12         Constrained at       0.9 Check
PLAT300_ALERT_4_G Atom Site Occupancy of P3          Constrained at       0.1 Check
PLAT300_ALERT_4_G Atom Site Occupancy of F13         Constrained at       0.1 Check
PLAT300_ALERT_4_G Atom Site Occupancy of F14         Constrained at       0.1 Check
PLAT300_ALERT_4_G Atom Site Occupancy of F15         Constrained at       0.1 Check
PLAT300_ALERT_4_G Atom Site Occupancy of F16         Constrained at       0.1 Check
PLAT300_ALERT_4_G Atom Site Occupancy of F17         Constrained at       0.1 Check
PLAT300_ALERT_4_G Atom Site Occupancy of F18         Constrained at       0.1 Check
PLAT301_ALERT_3_G Main Residue  Disorder .............(Resd    1)          3% Note
PLAT302_ALERT_4_G Anion/Solvent/Minor-Residue Disorder (Resd    4)       100% Note
PLAT302_ALERT_4_G Anion/Solvent/Minor-Residue Disorder (Resd    5)       100% Note
PLAT304_ALERT_4_G Non-Integer Number of Atoms in ..... (Resd    4)       6.30 Check
PLAT304_ALERT_4_G Non-Integer Number of Atoms in ..... (Resd    5)       0.70 Check
PLAT794_ALERT_5_G Tentative Bond Valency for Pd2       (II)      .       2.17 Info
PLAT860_ALERT_3_G Number of Least-Squares Restraints ............         299 Note
PLAT883_ALERT_1_G Absent Datum for _atom_sites_solution_primary ..     Please Do !
PLAT910_ALERT_3_G Missing FCF Reflection(s) Below Theta(Min)[Deg]=       1.92 Note
                1  0  0,   0  1  0,   0  0  1,   0  1  1,
PLAT933_ALERT_2_G Number of HKL-OMIT Records in Embedded .res File          1 Note
                1  1  3,
PLAT967_ALERT_5_G Note: Two-Theta Cutoff Value in Embedded .res ..       54.0 Degree
PLAT969_ALERT_5_G The 'Henn et al.' R-Factor-gap value ..........       3.481 Note
              Predicted wR2: Based on SigI**2  2.01 or SHELX Weight  6.38
```

```
PLAT978_ALERT_2_G Number C-C Bonds with Positive Residual Density.         6 Info
```

```
  0 ALERT level A = Most likely a serious problem - resolve or explain
  0 ALERT level B = A potentially serious problem, consider carefully
  3 ALERT level C = Check. Ensure it is not caused by an omission or oversight
 42 ALERT level G = General information/check it is not something unexpected

  2 ALERT type 1 CIF construction/syntax error, inconsistent or missing data
  9 ALERT type 2 Indicator that the structure model may be wrong or deficient
  7 ALERT type 3 Indicator that the structure quality may be low
 24 ALERT type 4 Improvement, methodology, query or suggestion
  3 ALERT type 5 Informative message, check
```

It is advisable to attempt to resolve as many as possible of the alerts in all categories. Often the minor alerts point to easily fixed oversights, errors and omissions in your CIF or refinement strategy, so attention to these fine details can be worthwhile. In order to resolve some of the more serious problems it may be necessary to carry out additional measurements or structure refinements. However, the purpose of your study may justify the reported deviations and the more serious of these should normally be commented upon in the discussion or experimental section of a paper or in the "special_details" fields of the CIF. checkCIF was carefully designed to identify outliers and unusual parameters, but every test has its limitations and alerts that are not important in a particular case may appear. Conversely, the absence of alerts does not guarantee there are no aspects of the results needing attention. It is up to the individual to critically assess their own results and, if necessary, seek expert advice.

Publication of your CIF in IUCr journals

A basic structural check has been run on your CIF. These basic checks will be run on all CIFs submitted for publication in IUCr journals (*Acta Crystallographica*, *Journal of Applied Crystallography*, *Journal of Synchrotron Radiation*); however, if you intend to submit to *Acta Crystallographica Section C* or *E* or *IUCrData*, you should make sure that full publication checks are run on the final version of your CIF prior to submission.

Publication of your CIF in other journals

Please refer to the *Notes for Authors* of the relevant journal for any special instructions relating to CIF submission.

PLATON version of 04/06/2025; check.def file version of 30/05/2025

Datablock mo_2025_fk118_4_0m - ellipsoid plot

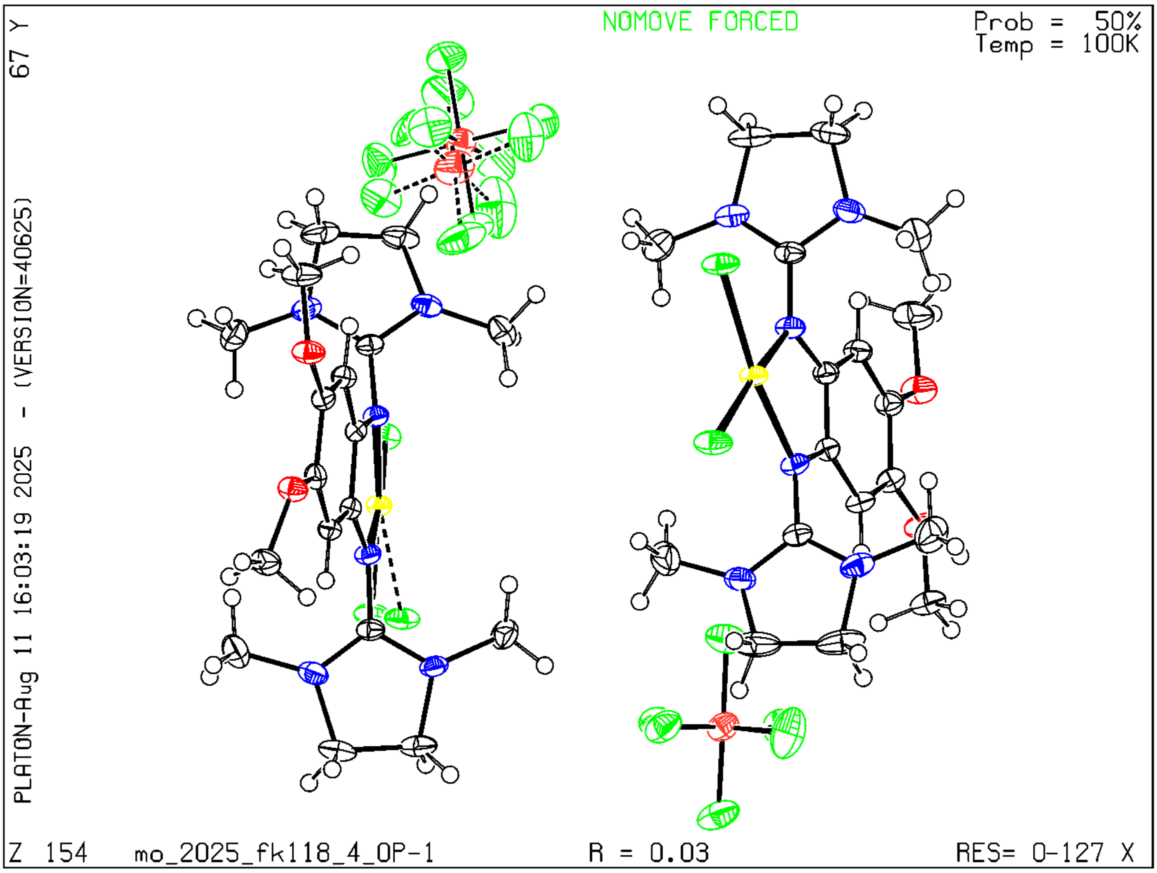

Supplement: Supplementary file 2 — Supporting Information [file CHEM-31-e03160-s002.zip › mo_2025_fk118_4_0m_cifreport.pdf]
