# Supplementary material for: Bistability in Palladium Complexes with Two Different Redox‐Active Ligands of Orthogonal Charge Regimes
Source: Chemistry. 2025 Nov 4;31(69):e03160. doi: 10.1002/chem.202503160 (PMC12699171; doi:10.1002/chem.202503160)

# checkCIF/PLATON report

Structure factors have been supplied for datablock(s) mo_2025_fkfb04_4_0m

THIS REPORT IS FOR GUIDANCE ONLY. IF USED AS PART OF A REVIEW PROCEDURE FOR PUBLICATION, IT SHOULD NOT REPLACE THE EXPERTISE OF AN EXPERIENCED CRYSTALLOGRAPHIC REFEREE.

No syntax errors found. CIF dictionary Interpreting this report

# Datablock: mo_2025_fkfb04_4_0m

---

```
Bond precision:   C-C = 0.0062 A          Wavelength=0.71073

Cell:            a=11.8888(8)       b=19.5285(14)      c=23.0636(16)
                 alpha=82.053(3)    beta=76.208(3)     gamma=81.176(2)
Temperature:     100 K
```

| | Calculated | Reported |
|---|---|---|
| Volume | 5109.8(6) | 5109.8(6) |
| Space group | P -1 | P-1 |
| Hall group | -P 1 | -P 1 |
| Moiety formula | C33 H30 Cl2 N6 O4 Pd, F6 P, 1.125(C6 H4 F2) [+ solvent] | C33 H30 Cl2 N6 O4 Pd, F6 P, 1.125(C6 H4 F2), 1.5[C6H4F2] |
| Sum formula | C39.75 H34.50 Cl2 F8.25 N6 O4 P Pd [+ solvent] | C48.75 H40.50 Cl2 F11.25 N6 O4 P Pd |
| Mr | 1025.25 | 1196.39 |
| Dx,g cm-3 | 1.333 | 1.555 |
| Z | 4 | 4 |
| Mu (mm-1) | 0.570 | 0.590 |
| F000 | 2065.0 | 2413.0 |
| F000' | 2063.72 | |
| h,k,lmax | 15,24,29 | 15,24,29 |
| Nref | 22301 | 22277 |
| Tmin,Tmax | 0.826,0.863 | 0.602,0.746 |
| Tmin' | 0.799 | |

```
Correction method= # Reported T Limits: Tmin=0.602 Tmax=0.746
AbsCorr = MULTI-SCAN

Data completeness= 0.999           Theta(max)= 27.000
```

R(reflections)= 0.0567( 19151)    wR2(reflections)= 0.1663( 22277)

S = 1.077    Npar= 1303

---

The following ALERTS were generated. Each ALERT has the format
**test-name_ALERT_alert-type_alert-level.**
Click on the hyperlinks for more details of the test.

---

## Alert level C

PLAT220_ALERT_2_C NonSolvent Resd 1 C Ueq(max)/Ueq(min) Range 3.5 Ratio
PLAT242_ALERT_2_C Low 'MainMol' Ueq as Compared to Neighbors of C75 Check
PLAT250_ALERT_2_C Large U3/U1 Ratio for <U(i,j)> Tensor(Resd 2) 2.1 Note
PLAT250_ALERT_2_C Large U3/U1 Ratio for <U(i,j)> Tensor(Resd 6) 3.2 Note
PLAT334_ALERT_2_C Small <C-C> Benzene Dist. C34 -C86 . 1.37 Ang.
PLAT334_ALERT_2_C Small <C-C> Benzene Dist. C87 -C92 . 1.37 Ang.
PLAT911_ALERT_3_C Missing FCF Refl Between Thmin & STh/L= 0.600 21 Report
-1 3 0, 0 -1 1, -5 0 1, 6 0 1, 1 5 1, -5 2 2,
2 -1 3, 6 0 3, -6 1 4, 6 1 4, 0 2 4, -1 -1 5,
7 0 5, -6 2 5, 6 3 6, -5 3 10, 8 4 10, 7 4 11,
-5 2 14, -1-11 16, 6 5 18,
PLAT918_ALERT_3_C Reflection(s) with I(obs) much Smaller I(calc) . 4 Check
PLAT973_ALERT_2_C Check Calcd Positive Resid. Density on Pd1 1.08 eA-3
PLAT975_ALERT_2_C Check Calcd Resid. Dens. 0.69Ang From O4 . 0.68 eA-3

---

## Alert level G

FORMU01_ALERT_2_G There is a discrepancy between the atom counts in the
_chemical_formula_sum and the formula from the _atom_site* data.
Atom count from _chemical_formula_sum:C48.75 H40.5 Cl2 F11.25 N6 O4 P1
Atom count from the _atom_site data: C39.75 H34.5 Cl2 F8.25 N6 O4 P1
CELLZ01_ALERT_1_G Difference between formula and atom_site contents detected.
CELLZ01_ALERT_1_G ALERT: Large difference may be due to a
symmetry error - see SYMMG tests
From the CIF: _cell_formula_units_Z 4
From the CIF: _chemical_formula_sum C48.75 H40.50 Cl2 F11.25 N6 O4 P P
TEST: Compare cell contents of formula and atom_site data

| atom | Z*formula | cif sites | diff |
|---|---|---|---|
| C | 195.00 | 159.00 | 36.00 |
| H | 162.00 | 138.00 | 24.00 |
| Cl | 8.00 | 8.00 | 0.00 |
| F | 45.00 | 33.00 | 12.00 |
| N | 24.00 | 24.00 | 0.00 |
| O | 16.00 | 16.00 | 0.00 |
| P | 4.00 | 4.00 | 0.00 |
| Pd | 4.00 | 4.00 | 0.00 |

PLAT002_ALERT_2_G Number of Distance or Angle Restraints on AtSite 45 Note
PLAT003_ALERT_2_G Number of Uiso or U(i,j) Restrained non-H-Atoms 51 Report
PLAT041_ALERT_1_G Calc. and Reported SumFormula Strings Differ Please Check
Calc: C39.75 H34.50 Cl2 F8.25 N6 O4 P Pd
Rep.: C48.75 H40.50 Cl2 F11.25 N6 O4 P Pd
PLAT042_ALERT_1_G Calc. and Reported MoietyFormula Strings Differ Please Check
Calc: C33 H30 Cl2 N6 O4 Pd, F6 P, 1.125(C6 H4 F2)
Rep.: C33 H30 Cl2 N6 O4 Pd, F6 P, 1.125(C6 H4 F2), 1

```
                    .5[C6H4F2]
PLAT083_ALERT_2_G SHELXL Second Parameter in WGHT  Unusually Large      16.56 Why ?
PLAT172_ALERT_4_G The CIF-Embedded .res File Contains DFIX Records         16 Report
PLAT173_ALERT_4_G The CIF-Embedded .res File Contains DANG Records         18 Report
PLAT174_ALERT_4_G The CIF-Embedded .res File Contains FLAT Records          5 Report
PLAT176_ALERT_4_G The CIF-Embedded .res File Contains SADI Records         23 Report
PLAT178_ALERT_4_G The CIF-Embedded .res File Contains SIMU Records          7 Report
PLAT187_ALERT_4_G The CIF-Embedded .res File Contains RIGU Records          7 Report
PLAT191_ALERT_3_G A Non-default SADI Restraint Value has been used     0.0400 Report
PLAT191_ALERT_3_G A Non-default SADI Restraint Value has been used     0.0400 Report
PLAT191_ALERT_3_G A Non-default SADI Restraint Value has been used     0.0500 Report
PLAT191_ALERT_3_G A Non-default SADI Restraint Value has been used     0.0400 Report
PLAT191_ALERT_3_G A Non-default SADI Restraint Value has been used     0.0400 Report
PLAT191_ALERT_3_G A Non-default SADI Restraint Value has been used     0.0400 Report
PLAT191_ALERT_3_G A Non-default SADI Restraint Value has been used     0.0400 Report
PLAT191_ALERT_3_G A Non-default SADI Restraint Value has been used     0.0400 Report
PLAT191_ALERT_3_G A Non-default SADI Restraint Value has been used     0.0400 Report
PLAT191_ALERT_3_G A Non-default SADI Restraint Value has been used     0.0400 Report
PLAT191_ALERT_3_G A Non-default SADI Restraint Value has been used     0.0400 Report
PLAT191_ALERT_3_G A Non-default SADI Restraint Value has been used     0.0400 Report
PLAT231_ALERT_4_G Hirshfeld Test (Solvent)   P2       --F11      .        5.2 s.u.
PLAT232_ALERT_2_G Hirshfeld Test Diff (M-X)  Pd2      --O54      .        7.1 s.u.
PLAT244_ALERT_4_G Low    'Solvent' Ueq as Compared to Neighbors of         P2 Check
PLAT244_ALERT_4_G Low    'Solvent' Ueq as Compared to Neighbors of         P1 Check
PLAT300_ALERT_4_G Atom Site Occupancy of Cl51       Constrained at       0.85 Check
PLAT300_ALERT_4_G Atom Site Occupancy of Cl52       Constrained at       0.85 Check
PLAT300_ALERT_4_G Atom Site Occupancy of Cl53       Constrained at       0.15 Check
PLAT300_ALERT_4_G Atom Site Occupancy of Cl54       Constrained at       0.15 Check
PLAT300_ALERT_4_G Atom Site Occupancy of C80        Constrained at       0.85 Check
PLAT300_ALERT_4_G Atom Site Occupancy of C81        Constrained at       0.85 Check
PLAT300_ALERT_4_G Atom Site Occupancy of C82        Constrained at       0.85 Check
PLAT300_ALERT_4_G Atom Site Occupancy of C83        Constrained at       0.85 Check
PLAT300_ALERT_4_G Atom Site Occupancy of C80B       Constrained at       0.15 Check
PLAT300_ALERT_4_G Atom Site Occupancy of C81B       Constrained at       0.15 Check
PLAT300_ALERT_4_G Atom Site Occupancy of C82B       Constrained at       0.15 Check
PLAT300_ALERT_4_G Atom Site Occupancy of C83B       Constrained at       0.15 Check
PLAT300_ALERT_4_G Atom Site Occupancy of H80        Constrained at       0.85 Check
PLAT300_ALERT_4_G Atom Site Occupancy of H83        Constrained at       0.85 Check
PLAT300_ALERT_4_G Atom Site Occupancy of H80B       Constrained at       0.15 Check
PLAT300_ALERT_4_G Atom Site Occupancy of H83B       Constrained at       0.15 Check
PLAT300_ALERT_4_G Atom Site Occupancy of F9         Constrained at        0.7 Check
PLAT300_ALERT_4_G Atom Site Occupancy of F10        Constrained at        0.7 Check
PLAT300_ALERT_4_G Atom Site Occupancy of F11        Constrained at        0.7 Check
PLAT300_ALERT_4_G Atom Site Occupancy of F12        Constrained at        0.7 Check
PLAT300_ALERT_4_G Atom Site Occupancy of F9B        Constrained at        0.3 Check
PLAT300_ALERT_4_G Atom Site Occupancy of F10B       Constrained at        0.3 Check
PLAT300_ALERT_4_G Atom Site Occupancy of F11B       Constrained at        0.3 Check
PLAT300_ALERT_4_G Atom Site Occupancy of F12B       Constrained at        0.3 Check
PLAT300_ALERT_4_G Atom Site Occupancy of F81        Constrained at       0.75 Check
PLAT300_ALERT_4_G Atom Site Occupancy of F82        Constrained at       0.75 Check
PLAT300_ALERT_4_G Atom Site Occupancy of C34        Constrained at       0.75 Check
PLAT300_ALERT_4_G Atom Site Occupancy of C35        Constrained at       0.75 Check
PLAT300_ALERT_4_G Atom Site Occupancy of C36        Constrained at       0.75 Check
PLAT300_ALERT_4_G Atom Site Occupancy of C84        Constrained at       0.75 Check
PLAT300_ALERT_4_G Atom Site Occupancy of C85        Constrained at       0.75 Check
PLAT300_ALERT_4_G Atom Site Occupancy of C86        Constrained at       0.75 Check
PLAT300_ALERT_4_G Atom Site Occupancy of H36        Constrained at       0.75 Check
```

```
PLAT300_ALERT_4_G Atom Site Occupancy of H84        Constrained at       0.75 Check
PLAT300_ALERT_4_G Atom Site Occupancy of H85        Constrained at       0.75 Check
PLAT300_ALERT_4_G Atom Site Occupancy of H86        Constrained at       0.75 Check
PLAT300_ALERT_4_G Atom Site Occupancy of F83        Constrained at       0.75 Check
PLAT300_ALERT_4_G Atom Site Occupancy of F84        Constrained at       0.75 Check
PLAT300_ALERT_4_G Atom Site Occupancy of C87        Constrained at       0.75 Check
PLAT300_ALERT_4_G Atom Site Occupancy of C88        Constrained at       0.75 Check
PLAT300_ALERT_4_G Atom Site Occupancy of C89        Constrained at       0.75 Check
PLAT300_ALERT_4_G Atom Site Occupancy of C90        Constrained at       0.75 Check
PLAT300_ALERT_4_G Atom Site Occupancy of C91        Constrained at       0.75 Check
PLAT300_ALERT_4_G Atom Site Occupancy of C92        Constrained at       0.75 Check
PLAT300_ALERT_4_G Atom Site Occupancy of H89        Constrained at       0.75 Check
PLAT300_ALERT_4_G Atom Site Occupancy of H90        Constrained at       0.75 Check
PLAT300_ALERT_4_G Atom Site Occupancy of H91        Constrained at       0.75 Check
PLAT300_ALERT_4_G Atom Site Occupancy of H92        Constrained at       0.75 Check
PLAT300_ALERT_4_G Atom Site Occupancy of F85        Constrained at       0.75 Check
PLAT300_ALERT_4_G Atom Site Occupancy of F86        Constrained at       0.75 Check
PLAT300_ALERT_4_G Atom Site Occupancy of C93        Constrained at       0.75 Check
PLAT300_ALERT_4_G Atom Site Occupancy of C94        Constrained at       0.75 Check
PLAT300_ALERT_4_G Atom Site Occupancy of C95        Constrained at       0.75 Check
PLAT300_ALERT_4_G Atom Site Occupancy of C96        Constrained at       0.75 Check
PLAT300_ALERT_4_G Atom Site Occupancy of C97        Constrained at       0.75 Check
PLAT300_ALERT_4_G Atom Site Occupancy of C98        Constrained at       0.75 Check
PLAT300_ALERT_4_G Atom Site Occupancy of H95        Constrained at       0.75 Check
PLAT300_ALERT_4_G Atom Site Occupancy of H96        Constrained at       0.75 Check
PLAT300_ALERT_4_G Atom Site Occupancy of H97        Constrained at       0.75 Check
PLAT300_ALERT_4_G Atom Site Occupancy of H98        Constrained at       0.75 Check
PLAT301_ALERT_3_G Main Residue  Disorder .............(Resd    1)        13% Note
PLAT302_ALERT_4_G Anion/Solvent/Minor-Residue Disorder (Resd    2)         7% Note
PLAT302_ALERT_4_G Anion/Solvent/Minor-Residue Disorder (Resd    3)        57% Note
PLAT302_ALERT_4_G Anion/Solvent/Minor-Residue Disorder (Resd    4)       100% Note
PLAT302_ALERT_4_G Anion/Solvent/Minor-Residue Disorder (Resd    5)       100% Note
PLAT302_ALERT_4_G Anion/Solvent/Minor-Residue Disorder (Resd    6)       100% Note
PLAT398_ALERT_2_G Deviating  C-O-C   Angle From 120 for O51      .      108.0 Degree
PLAT398_ALERT_2_G Deviating  C-O-C   Angle From 120 for O52      .      107.0 Degree
PLAT398_ALERT_2_G Deviating  C-O-C   Angle From 120 for O1       .      106.3 Degree
PLAT398_ALERT_2_G Deviating  C-O-C   Angle From 120 for O2       .      109.2 Degree
PLAT432_ALERT_2_G Short Inter X...Y Contact  F84      ..C8       .       2.84 Ang.
                                              x,-1+y,z  =      1_545 Check
PLAT432_ALERT_2_G Short Inter X...Y Contact  O51      ..C34      .       3.00 Ang.
                                            1-x,1-y,-z  =      2_665 Check
PLAT606_ALERT_4_G Solvent Accessible VOID(S) in Structure ........          ! Info
PLAT790_ALERT_4_G Centre of Gravity not Within Unit Cell: Resd.  #          4 Note
              C6 H4 F2
PLAT794_ALERT_5_G Tentative Bond Valency for Pd1       (II)      .       2.32 Info
PLAT860_ALERT_3_G Number of Least-Squares Restraints ............         963 Note
PLAT868_ALERT_4_G ALERTS Due to the Use of _smtbx_masks Suppressed          ! Info
PLAT883_ALERT_1_G Absent Datum for _atom_sites_solution_primary ..     Please Do !
PLAT910_ALERT_3_G Missing # of FCF Reflection(s) Below Theta(Min).          2 Note
                0  1  0,   0  0  1,
PLAT913_ALERT_3_G Missing # of Very Strong Reflections in FCF ....          1 Note
                2 -1  3,
PLAT933_ALERT_2_G Number of HKL-OMIT Records in Embedded .res File          6 Note
               -1 -1  5,   0 -1  1,   0  0  1,   0  1  0,   0  2  4,   1  5  1,
PLAT967_ALERT_5_G Note: Two-Theta Cutoff Value in Embedded .res ..       54.0 Degree
PLAT969_ALERT_5_G The 'Henn et al.' R-Factor-gap value ..........      5.363 Note
              Predicted wR2: Based on SigI**2  3.10 or SHELX Weight 15.44
```

```
PLAT978_ALERT_2_G Number C-C Bonds with Positive Residual Density.         0 Info
```

---

```
  0 ALERT level A = Most likely a serious problem - resolve or explain
  0 ALERT level B = A potentially serious problem, consider carefully
 10 ALERT level C = Check. Ensure it is not caused by an omission or oversight
114 ALERT level G = General information/check it is not something unexpected

  5 ALERT type 1 CIF construction/syntax error, inconsistent or missing data
 21 ALERT type 2 Indicator that the structure model may be wrong or deficient
 18 ALERT type 3 Indicator that the structure quality may be low
 77 ALERT type 4 Improvement, methodology, query or suggestion
  3 ALERT type 5 Informative message, check
```

---

It is advisable to attempt to resolve as many as possible of the alerts in all categories. Often the minor alerts point to easily fixed oversights, errors and omissions in your CIF or refinement strategy, so attention to these fine details can be worthwhile. In order to resolve some of the more serious problems it may be necessary to carry out additional measurements or structure refinements. However, the purpose of your study may justify the reported deviations and the more serious of these should normally be commented upon in the discussion or experimental section of a paper or in the "special_details" fields of the CIF. checkCIF was carefully designed to identify outliers and unusual parameters, but every test has its limitations and alerts that are not important in a particular case may appear. Conversely, the absence of alerts does not guarantee there are no aspects of the results needing attention. It is up to the individual to critically assess their own results and, if necessary, seek expert advice.

**Publication of your CIF in IUCr journals**

A basic structural check has been run on your CIF. These basic checks will be run on all CIFs submitted for publication in IUCr journals (*Acta Crystallographica*, *Journal of Applied Crystallography*, *Journal of Synchrotron Radiation*); however, if you intend to submit to *Acta Crystallographica Section C* or *E* or *IUCrData*, you should make sure that full publication checks are run on the final version of your CIF prior to submission.

**Publication of your CIF in other journals**

Please refer to the *Notes for Authors* of the relevant journal for any special instructions relating to CIF submission.

---

**PLATON version of 02/02/2025; check.def file version of 02/02/2025**

**Datablock mo_2025_fkfb04_4_0m** - ellipsoid plot

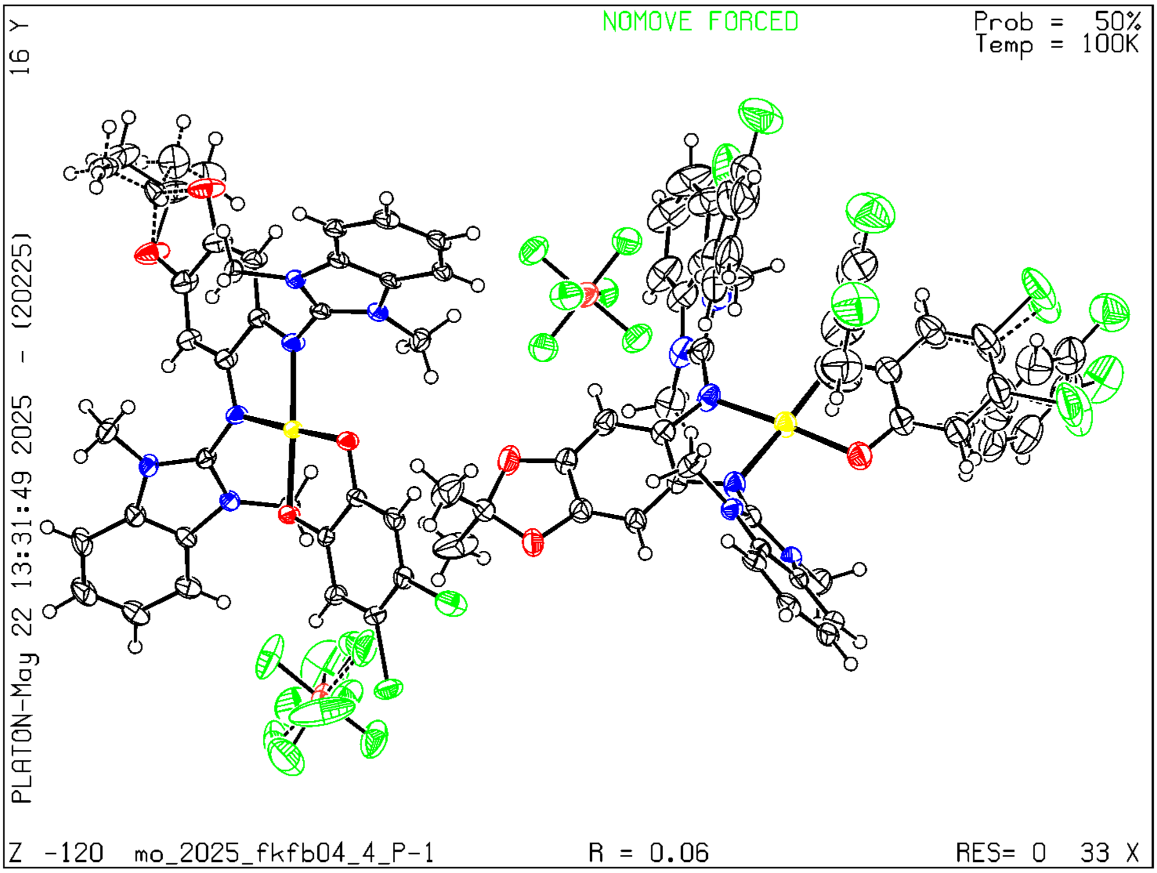

Supplement: Supplementary file 2 — Supporting Information [file CHEM-31-e03160-s002.zip › mo_2025_fkfb04_4_0m_cifreport.pdf]
