# Supplementary material for: Bistability in Palladium Complexes with Two Different Redox‐Active Ligands of Orthogonal Charge Regimes
Source: Chemistry. 2025 Nov 4;31(69):e03160. doi: 10.1002/chem.202503160 (PMC12699171; doi:10.1002/chem.202503160)

checkCIF/PLATON report

Structure factors have been supplied for datablock(s) mo_2025_fklh06_3_0ma

THIS REPORT IS FOR GUIDANCE ONLY. IF USED AS PART OF A REVIEW PROCEDURE FOR PUBLICATION, IT SHOULD NOT REPLACE THE EXPERTISE OF AN EXPERIENCED CRYSTALLOGRAPHIC REFEREE.

No syntax errors found. CIF dictionary Interpreting this report

Datablock: mo_2025_fklh06_3_0ma

Bond precision:	C-C = 0.0057 A		Wavelength=0.71073
Cell:	a=16.2857(15)	b=29.230(4)	c=11.6162(12)
	alpha=90	beta=102.606(3)	gamma=90
Temperature:	100 K		

	Calculated	Reported
Volume	5396.4(11)	5396.3(10)
Space group	C 2/c	C2/c
Hall group	-C 2yc	-C 2yc
Moiety formula	C37 H39 N6 O4 Pd, 2(F6 P) [+ solvent]	C37 H39 N6 O4 Pd, 2(F6 P), 1.4[C6H4F2]
Sum formula	C37 H39 F12 N6 O4 P2 Pd [+ solvent]	C45.40 H44.60 F14.80 N6 O4 P2 Pd
Mr	1028.08	1187.81
Dx,g cm-3	1.265	1.462
Z	4	4
Mu (mm-1)	0.481	0.500
F000	2076.0	2401.0
F000'	2074.15	
h,k,lmax	20,37,14	20,37,14
Nref	5912	5899
Tmin,Tmax	0.955,0.978	0.618,0.746
Tmin'	0.799	

Correction method= # Reported T Limits: Tmin=0.618 Tmax=0.746
AbsCorr = MULTI-SCAN

Data completeness= 0.998 Theta(max)= 27.000

```
R(reflections)= 0.0516( 4948)            wR2(reflections)= 0.1431( 5899)
S = 1.039                  Npar= 305
```

The following ALERTS were generated. Each ALERT has the format
test-name_ALERT_alert-type_alert-level.
Click on the hyperlinks for more details of the test.

Alert level B

```
PLAT220_ALERT_2_B NonSolvent   Resd 1  C   Ueq(max)/Ueq(min) Range        7.5 Ratio
```

Author Response: Due to un-resolved disorder of tert. butylgroup.

Alert level C

```
PLAT215_ALERT_3_C Disordered C20       has ADP max/min Ratio .....         3.3 Note
PLAT222_ALERT_3_C NonSolvent Resd 1  H   Uiso(max)/Uiso(min) Range        7.8 Ratio
PLAT906_ALERT_3_C Large K Value in the Analysis of Variance ......      2.846 Check
PLAT911_ALERT_3_C Missing FCF Refl Between Thmin & STh/L=    0.600           8 Report
                4  0  0,   5  1  0,   5  3  0,   0  2  1,  -3  3  1,  -1  3  1,
               -4  4  1,  -7  1  2,
```

Alert level G

```
FORMU01_ALERT_2_G  There is a discrepancy between the atom counts in the
            _chemical_formula_sum and the formula from the _atom_site* data.
            Atom count from _chemical_formula_sum:C45.4 H44.6 F14.8 N6 O4 P2 Pd1
            Atom count from the _atom_site data:  C37 H39 F12 N6 O4 P2 Pd1
CELLZ01_ALERT_1_G Difference between formula and atom_site contents detected.
CELLZ01_ALERT_1_G ALERT: Large difference may be due to a
            symmetry error - see SYMMG tests
           From the CIF: _cell_formula_units_Z    4
           From the CIF: _chemical_formula_sum  C45.40 H44.60 F14.80 N6 O4 P2 Pd
           TEST: Compare cell contents of formula and atom_site data

           atom    Z*formula  cif sites diff
           C        181.60    148.00   33.60
           H        178.40    156.00   22.40
           F         59.20     48.00   11.20
           N         24.00     24.00    0.00
           O         16.00     16.00    0.00
           P          8.00      8.00    0.00
           Pd         4.00      4.00    0.00
PLAT003_ALERT_2_G Number of Uiso or U(i,j)  Restrained non-H-Atoms           4 Report
PLAT041_ALERT_1_G Calc. and Reported SumFormula    Strings  Differ     Please Check
              Calc: C37 H39 F12 N6 O4 P2 Pd
              Rep.: C45.40 H44.60 F14.80 N6 O4 P2 Pd
PLAT042_ALERT_1_G Calc. and Reported MoietyFormula Strings  Differ     Please Check
              Calc: C37 H39 N6 O4 Pd, 2(F6 P)
              Rep.: C37 H39 N6 O4 Pd, 2(F6 P), 1.4[C6H4F2]
PLAT083_ALERT_2_G SHELXL Second Parameter in WGHT  Unusually Large      16.91 Why ?
```

```
PLAT178_ALERT_4_G The CIF-Embedded .res File Contains SIMU Records          1 Report
PLAT187_ALERT_4_G The CIF-Embedded .res File Contains RIGU Records          1 Report
PLAT244_ALERT_4_G Low    'Solvent' Ueq as Compared to Neighbors of         P1 Check
PLAT299_ALERT_4_G Atom Site Occupancy Constrained at ............         0.5 Check
              C18     C19     C20     C21     H19A    H19B    H19C    H20A
              H20B    H20C    H21A    H21B    H21C
PLAT301_ALERT_3_G Main Residue  Disorder .............(Resd    1)          8% Note
PLAT398_ALERT_2_G Deviating  C-O-C   Angle From 120 for O1       .      108.4 Degree
PLAT412_ALERT_2_G Short Intra XH3 .. XHn     H16      ..H19C     .       2.03 Ang.
                                                      x,y,z  =      1_555 Check
PLAT432_ALERT_2_G Short Inter X...Y Contact  C12      ..C12      .       3.12 Ang.
                                            3/2-x,1/2-y,2-z  =      7_657 Check
PLAT605_ALERT_4_G Largest Solvent Accessible VOID in the Structure        694 A**3
PLAT790_ALERT_4_G Centre of Gravity not Within Unit Cell: Resd.  #          2 Note
              F6 P
PLAT794_ALERT_5_G Tentative Bond Valency for Pd1       (II)      .       2.41 Info
PLAT860_ALERT_3_G Number of Least-Squares Restraints ............          36 Note
PLAT868_ALERT_4_G ALERTS Due to the Use of _smtbx_masks Suppressed          ! Info
PLAT910_ALERT_3_G Missing # of FCF Reflection(s) Below Theta(Min).          2 Note
                1  1  0,   0  2  0,
PLAT912_ALERT_4_G Missing # of FCF Reflections Above STh/L=  0.600          3 Note
PLAT933_ALERT_2_G Number of HKL-OMIT Records in Embedded .res File          8 Note
               -7  1  2,  -4  4  1,  -3  3  1,  -1  3  1,   0  2  1,   4  0  0,
                5  1  0,   5  3  0,
PLAT967_ALERT_5_G Note: Two-Theta Cutoff Value in Embedded .res ..       54.0 Degree
PLAT969_ALERT_5_G The 'Henn et al.' R-Factor-gap value ..........       4.073 Note
              Predicted wR2: Based on SigI**2  3.51 or SHELX Weight 13.76
PLAT978_ALERT_2_G Number C-C Bonds with Positive Residual Density.          1 Info
```

---

  0 **ALERT level A** = Most likely a serious problem - resolve or explain
  1 **ALERT level B** = A potentially serious problem, consider carefully
  4 **ALERT level C** = Check. Ensure it is not caused by an omission or oversight
 26 **ALERT level G** = General information/check it is not something unexpected

  4 ALERT type 1 CIF construction/syntax error, inconsistent or missing data
  9 ALERT type 2 Indicator that the structure model may be wrong or deficient
  7 ALERT type 3 Indicator that the structure quality may be low
  8 ALERT type 4 Improvement, methodology, query or suggestion
  3 ALERT type 5 Informative message, check

It is advisable to attempt to resolve as many as possible of the alerts in all categories. Often the minor alerts point to easily fixed oversights, errors and omissions in your CIF or refinement strategy, so attention to these fine details can be worthwhile. In order to resolve some of the more serious problems it may be necessary to carry out additional measurements or structure refinements. However, the purpose of your study may justify the reported deviations and the more serious of these should normally be commented upon in the discussion or experimental section of a paper or in the "special_details" fields of the CIF. checkCIF was carefully designed to identify outliers and unusual parameters, but every test has its limitations and alerts that are not important in a particular case may appear. Conversely, the absence of alerts does not guarantee there are no aspects of the results needing attention. It is up to the individual to critically assess their own results and, if necessary, seek expert advice.

Publication of your CIF in IUCr journals

A basic structural check has been run on your CIF. These basic checks will be run on all CIFs submitted for publication in IUCr journals (*Acta Crystallographica*, *Journal of Applied Crystallography*, *Journal of Synchrotron Radiation*); however, if you intend to submit to *Acta Crystallographica Section C* or *E* or *IUCrData*, you should make sure that full publication checks are run on the final version of your CIF prior to submission.

Publication of your CIF in other journals

Please refer to the *Notes for Authors* of the relevant journal for any special instructions relating to CIF submission.

PLATON version of 02/02/2025; check.def file version of 02/02/2025

Datablock mo_2025_fklh06_3_0ma - ellipsoid plot

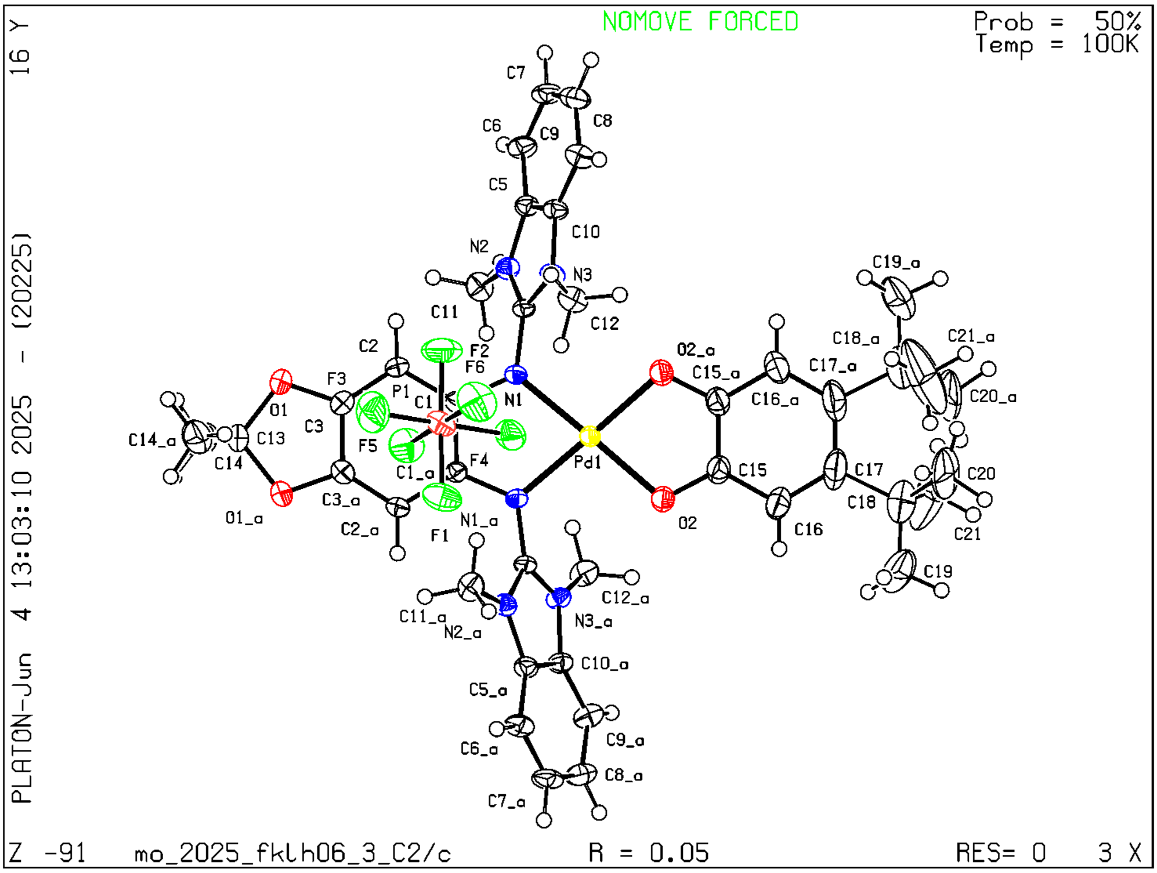

Supplement: Supplementary file 2 — Supporting Information [file CHEM-31-e03160-s002.zip › mo_2025_fklh06_3_0ma_cifreport.pdf]
