# Supplementary material for: Bistability in Palladium Complexes with Two Different Redox‐Active Ligands of Orthogonal Charge Regimes
Source: Chemistry. 2025 Nov 4;31(69):e03160. doi: 10.1002/chem.202503160 (PMC12699171; doi:10.1002/chem.202503160)
Supplement: Supplementary file 2 — Supporting Information [file CHEM-31-e03160-s002.zip › checkCIF_PLATON page 2.pdf]

checkCIF (basic structural check) running

Checking for embedded fcf data in CIF ...
Found embedded fcf data in CIF. Extracting fcf data from uploaded CIF, please wait . . . .

checkCIF/PLATON (basic structural check)

Structure factors have been supplied for datablock(s) mo_2025_fkpb19_1_0m

THIS REPORT IS FOR GUIDANCE ONLY. IF USED AS PART OF A REVIEW PROCEDURE FOR PUBLICATION, IT SHOULD NOT REPLACE THE EXPERTISE OF AN EXPERIENCED CRYSTALLOGRAPHIC REFEREE.

No syntax errors found. CIF dictionary
Please wait while processing .... Interpreting this report

Structure factor report

Datablock: mo_2025_fkpb19_1_0m

Bond precision:	C-C = 0.0035 A	Wavelength=0.71073
Cell:	a=14.9318(7) b=16.6707(8) c=19.7730(7)	
	alpha=73.984(2) beta=86.035(2) gamma=64.032(2)	
Temperature:	100 K	
	Calculated	Reported
Volume	4245.7(3)	4245.7(3)
Space group	P -1	P-1
Hall group	-P 1	-P 1
Moiety formula	2(C33 H30 Cl2 N6 O4 Pd), 2(C6 H4 Cl2 O2), C H2 Cl2 [+ solvent]	2(C33 H30 Cl2 N6 O4 Pd), 2(C6 H4 Cl2 O2), C H2 Cl2, 1[CH2CL2]
Sum formula	C79 H70 Cl10 N12 O12 Pd2 [+ solvent]	C80 H72 Cl12 N12 O12 Pd2
Mr	1946.77	2031.69
Dx,g cm-3	1.523	1.589
Z	2	2
Mu (mm-1)	0.804	0.869
F000	1972.0	2056.0
F000'	1971.65	
h,k,lmax	19,21,25	19,21,25
Nref	19503	19490
Tmin,Tmax	0.890,0.928	0.695,0.746
Tmin'	0.682	

Correction method= # Reported T Limits: Tmin=0.695 Tmax=0.746 AbsCorr = MULTI-SCAN

Data completeness= 0.999 Theta(max)= 27.500

R(reflections)= 0.0298(16853) wR2(reflections)= 0.0727(19490)

S = 1.029 Npar= 1052

The following ALERTS were generated. Each ALERT has the format
test-name_ALERT_alert-type_alert-level.
Click on the hyperlinks for more details of the test.

Alert level C

PLAT213_ALERT_2_C Atom C20 has ADP max/min Ratio ..... 3.3 prolat
PLAT230_ALERT_2_C Hirshfeld Test Diff for C19 --C20 . 5.5 s.u.
PLAT411_ALERT_2_C Short Inter H...H Contact H36 ..H73 . 2.02 Ang.
x,y,1+z = 1_556 Check
PLAT910_ALERT_3_C Missing # of FCF Reflection(s) Below Theta(Min). 8 Note
1 0 0, 0 1 0, 1 1 0, -1 0 1, 0 0 1, 1 0 1,
0 1 1, 1 1 1,
PLAT911_ALERT_3_C Missing FCF Refl Between Thmin & STh/L= 0.600 4 Report
1 1 2, -3 -1 3, 0 1 4, 0 2 6,

Alert level G

FORMU01_ALERT_2_G There is a discrepancy between the atom counts in the

_chemical_formula_sum and the formula from the _atom_site* data.
Atom count from _chemical_formula_sum:C80 H72 Cl12 N12 O12 Pd2
Atom count from the _atom_site data: C79 H70 Cl10 N12 O12 Pd2
CELLZ01_ALERT_1_G Difference between formula and atom_site contents detected.
CELLZ01_ALERT_1_G ALERT: Large difference may be due to a
symmetry error - see SYMMG tests
From the CIF: _cell_formula_units_Z 2
From the CIF: _chemical_formula_sum C80 H72 Cl12 N12 O12 Pd2
TEST: Compare cell contents of formula and atom_site data

atom	Z*formula	cif sites	diff
C	160.00	158.00	2.00
H	144.00	140.00	4.00
Cl	24.00	20.00	4.00
N	24.00	24.00	0.00
O	24.00	24.00	0.00
Pd	4.00	4.00	0.00

PLAT007_ALERT_5_G Number of Unrefined Donor-H Atoms .... 4 Report
H5 H6A H45 H46A
PLAT041_ALERT_1_G Calc. and Reported SumFormula Strings Differ Please Check
Calc: C79 H70 Cl10 N12 O12 Pd2
Rep.: C80 H72 Cl12 N12 O12 Pd2
PLAT042_ALERT_1_G Calc. and Reported MoietyFormula Strings Differ Please Check
Calc: 2(C33 H30 Cl2 N6 O4 Pd), 2(C6 H4 Cl2 O2), C H2
Cl2
Rep.: 2(C33 H30 Cl2 N6 O4 Pd), 2(C6 H4 Cl2 O2), C H2
Cl2, 1[CH2CL2]
PLAT051_ALERT_1_G Mu(calc) and Mu(cif) Ratio Differs from 1.0 by . 7.43 %
PLAT154_ALERT_1_G The s.u.'s on the Cell Angles are Equal ..(Note) 0.002 Degree
PLAT398_ALERT_2_G Deviating C-O-C Angle From 120 for O1 . 105.5 Degree
And 3 other PLAT398 Alerts
More ...
PLAT432_ALERT_2_G Short Inter X...Y Contact Cl41 ..C17 . 3.19 Ang.
-x,1-y,1-z = 2_566 Check
PLAT432_ALERT_2_G Short Inter X...Y Contact C35 ..C53 . 3.17 Ang.
x,y,1+z = 1_556 Check
PLAT605_ALERT_4_G Largest Solvent Accessible VOID in the Structure 185 A**3
PLAT790_ALERT_4_G Centre of Gravity not Within Unit Cell: Resd. # 5 Note
C H2 Cl2
PLAT794_ALERT_5_G Tentative Bond Valency for Pd1 (II) . 2.20 Info
PLAT794_ALERT_5_G Tentative Bond Valency for Pd2 (II) . 2.21 Info
PLAT868_ALERT_4_G ALERTS Due to the Use of _smtbx_masks Suppressed ! Info
PLAT883_ALERT_1_G Absent Datum for _atom_sites_solution_primary .. Please Do !
PLAT933_ALERT_2_G Number of HKL-OMIT Records in Embedded .res File 1 Note
1 1 2,
PLAT967_ALERT_5_G Note: Two-Theta Cutoff Value in Embedded .res .. 55.0 Degree
PLAT969_ALERT_5_G The 'Henn et al.' R-Factor-gap value .... 1.904 Note
Predicted wR2: Based on SigI**2 3.82 or SHELX Weight 7.07
PLAT978_ALERT_2_G Number C-C Bonds with Positive Residual Density. 8 Info

---

0 **ALERT level A** = Most likely a serious problem - resolve or explain
0 **ALERT level B** = A potentially serious problem, consider carefully
5 **ALERT level C** = Check. Ensure it is not caused by an omission or oversight
24 **ALERT level G** = General information/check it is not something unexpected

7 ALERT type 1 CIF construction/syntax error, inconsistent or missing data
12 ALERT type 2 Indicator that the structure model may be wrong or deficient
2 ALERT type 3 Indicator that the structure quality may be low
3 ALERT type 4 Improvement, methodology, query or suggestion
5 ALERT type 5 Informative message, check

Validation response form

Please find below a validation response form (VRF) that can be filled in and pasted into your CIF.

```
# start Validation Reply Form
_vrf_PLAT213_mo_2025_fkpb19_1_0m
;
PROBLEM: Atom C20            has ADP max/min Ratio .....       3.3 prolat
RESPONSE: ...
;
_vrf_PLAT230_mo_2025_fkpb19_1_0m
;
PROBLEM: Hirshfeld Test Diff for    C19      --C20      .      5.5 s.u.
RESPONSE: ...
;
```

```
_vrf_PLAT411_mo_2025_fkpb19_1_0m
;
PROBLEM: Short Inter H...H Contact  H36      ..H73      .       2.02 Ang.
RESPONSE: ...
;
_vrf_PLAT910_mo_2025_fkpb19_1_0m
;
PROBLEM: Missing # of FCF Reflection(s) Below Theta(Min).          8 Note
RESPONSE: ...
;
_vrf_PLAT911_mo_2025_fkpb19_1_0m
;
PROBLEM: Missing FCF Refl Between Thmin & STh/L=    0.600          4 Report
RESPONSE: ...
;
# end Validation Reply Form
```

It is advisable to attempt to resolve as many as possible of the alerts in all categories. Often the minor alerts point to easily fixed oversights, errors and omissions in your CIF or refinement strategy, so attention to these fine details can be worthwhile. In order to resolve some of the more serious problems it may be necessary to carry out additional measurements or structure refinements. However, the purpose of your study may justify the reported deviations and the more serious of these should normally be commented upon in the discussion or experimental section of a paper or in the "special_details" fields of the CIF. checkCIF was carefully designed to identify outliers and unusual parameters, but every test has its limitations and alerts that are not important in a particular case may appear. Conversely, the absence of alerts does not guarantee there are no aspects of the results needing attention. It is up to the individual to critically assess their own results and, if necessary, seek expert advice.

Publication of your CIF in IUCr journals

A basic structural check has been run on your CIF. These basic checks will be run on all CIFs submitted for publication in IUCr journals (*Acta Crystallographica*, *Journal of Applied Crystallography*, *Journal of Synchrotron Radiation*); however, if you intend to submit to *Acta Crystallographica Section C* or *E* or *IUCrData*, you should make sure that full publication checks are run on the final version of your CIF prior to submission.

Publication of your CIF in other journals

Please refer to the *Notes for Authors* of the relevant journal for any special instructions relating to CIF submission.

PLATON version of 02/02/2025; check.def file version of 02/02/2025

Datablock mo_2025_fkpb19_1_0m - ellipsoid plot

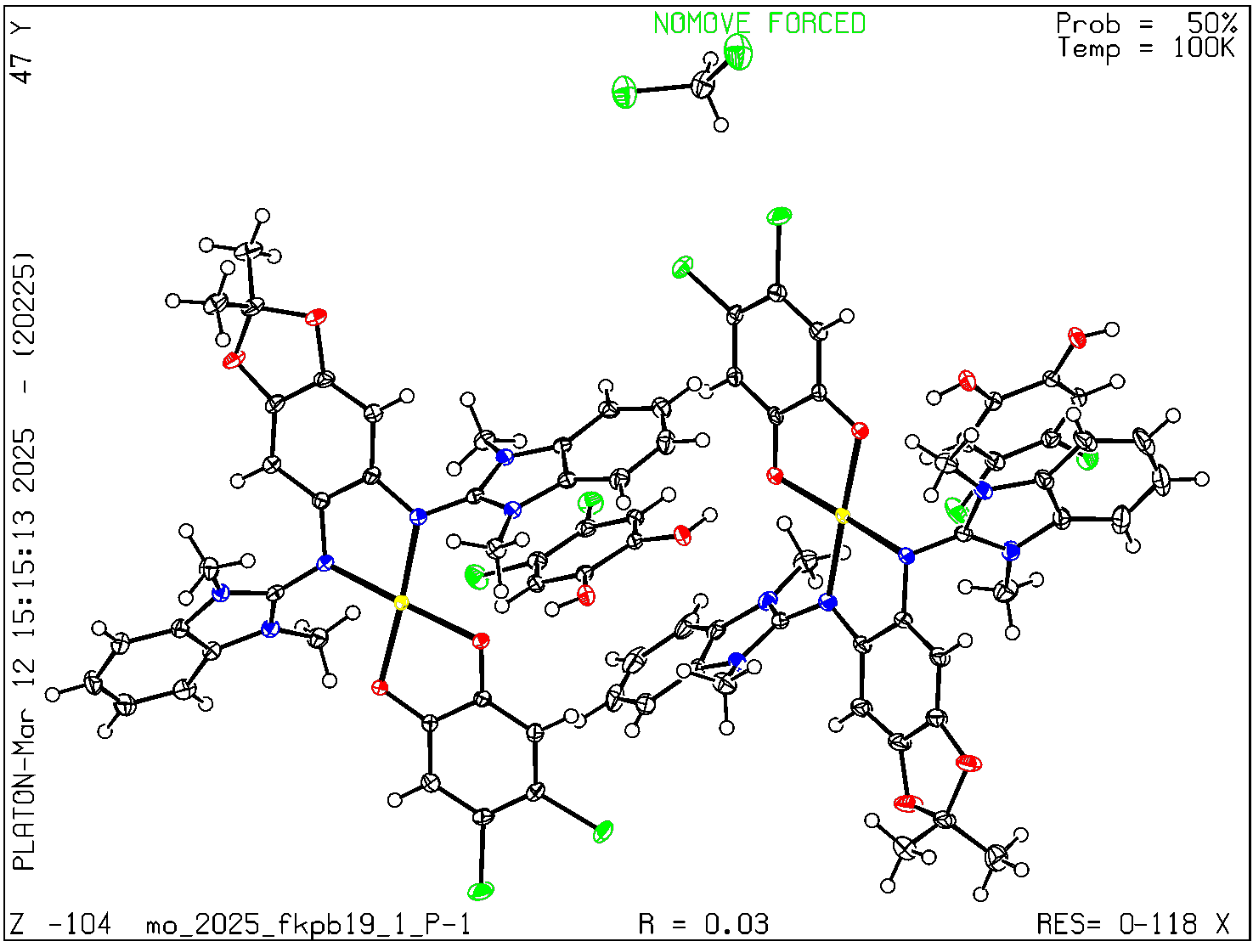

Download CIF editor (publCIF) from the IUCr
Download CIF editor (enCIFer) from the CCDC
Test a new CIF entry
